# Tunable Photoluminescent and Photothermal Properties of Organic Cocrystals Containing Hydrogen‐Bonded Interlocked Planar Molecules

**DOI:** 10.1002/advs.202515054

**Published:** 2025-09-29

**Authors:** Xinmeng Chen, Ling Zhu, Shan He, Bin Liu, Qiang Lv, Xuedong Wang, Lin Xu, Yu Wang, Ryan T. K. Kwok, Jacky W. Y. Lam, Lianrui Hu, Wenping Hu, Ben Zhong Tang

**Affiliations:** ^1^ Department of Chemistry and the Hong Kong Branch of Chinese National Engineering Research Center for Tissue Restoration and Reconstruction The Hong Kong University of Science and Technology Clear Water Bay Kowloon Hong Kong 999077 China; ^2^ Shanghai Key Laboratory of Green Chemistry and Chemical Processes Shanghai Frontiers Science Center of Molecule Intelligent Syntheses School of Chemistry and Molecular Engineering East China Normal University Shanghai 200062 China; ^3^ School of Chemistry Sun Yat‐Sen University Guangzhou 510006 China; ^4^ Institute of Functional Nano & Soft Material (FUNSOM) Jiangsu Key Laboratory for Carbon‐Based Functional Materials & Devices Soochow University Suzhou Jiangsu 215123 China; ^5^ Key Laboratory of Organic Integrated Circuit Ministry of Education & Tianjin Key Laboratory of Molecular Optoelectronic Sciences Department of Chemistry School of Science Tianjin University Tianjin 300072 China; ^6^ Guangdong Basic Research Center of Excellence for Aggregate Science School of Science and Engineering The Chinese University of Hong Kong Longgang Shenzhen Guangdong 518172 China

**Keywords:** charge transfer, cocrystal, luminescence, molecules, photothermal conversion

## Abstract

Expanding the structural diversity of precursor molecules can inject greater vitality into the development of cocrystal engineering. Here, two hydrogen‐bond interlocked planar molecules, HNAO and HPAO is reported, which coassemble with 1,2,4,5‐tetracyanobenzene (TCB) into charge‐transfer (CT) cocrystals NTC and PTC. The resulting NTC inherits two‐photon adsorption properties from HNAO, with the bathochromic shift enhanced by synergistic ESIPT and CT interactions, ultimately achieving near‐infrared emission. Remarkably, the intramolecular hydrogen bonds lock the molecule into a planar and ordered conformation, enabling regular face‐to‐face packing in NTC organic microwires and yielding the lowest optical‐loss coefficient (0.021 dB/µm) of organic cocrystal to date, thereby demonstrating great potential for applications in optical computing systems. In contrast, the extremely low energy gap between the HOMO of HPAO and the LUMO of TCB in PTC drives a dramatically higher degree of CT in its excited states, predominantly facilitating non‐radiative transitions and resulting in non‐emissive behavior. Nevertheless, this trade‐off enables efficient NIR‐I photothermal conversion (η = 47.7%) and excellent photostability, making PTC highly effective for rapid photothermal imaging and breakthrough time‐dependent information encryption applications. This work enriches the library of cocrystals and provides a novel strategy for tailoring the properties of cocrystal materials.

## Graphical abstract

Engineering intramolecular hydrogen‐bonded planar architectures provides a versatile strategy for modulating the photoluminescent and photothermal behaviors of organic cocrystals. This work not only overcomes the limitations of selecting conjugated (hetero)aromatic compounds to meet the requirements of planarity and rigidity for cocrystal precursors, but also bridges the gap between monomer and their cocrystals.

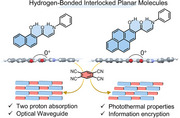

## 1 Introduction

**Figure 1:**
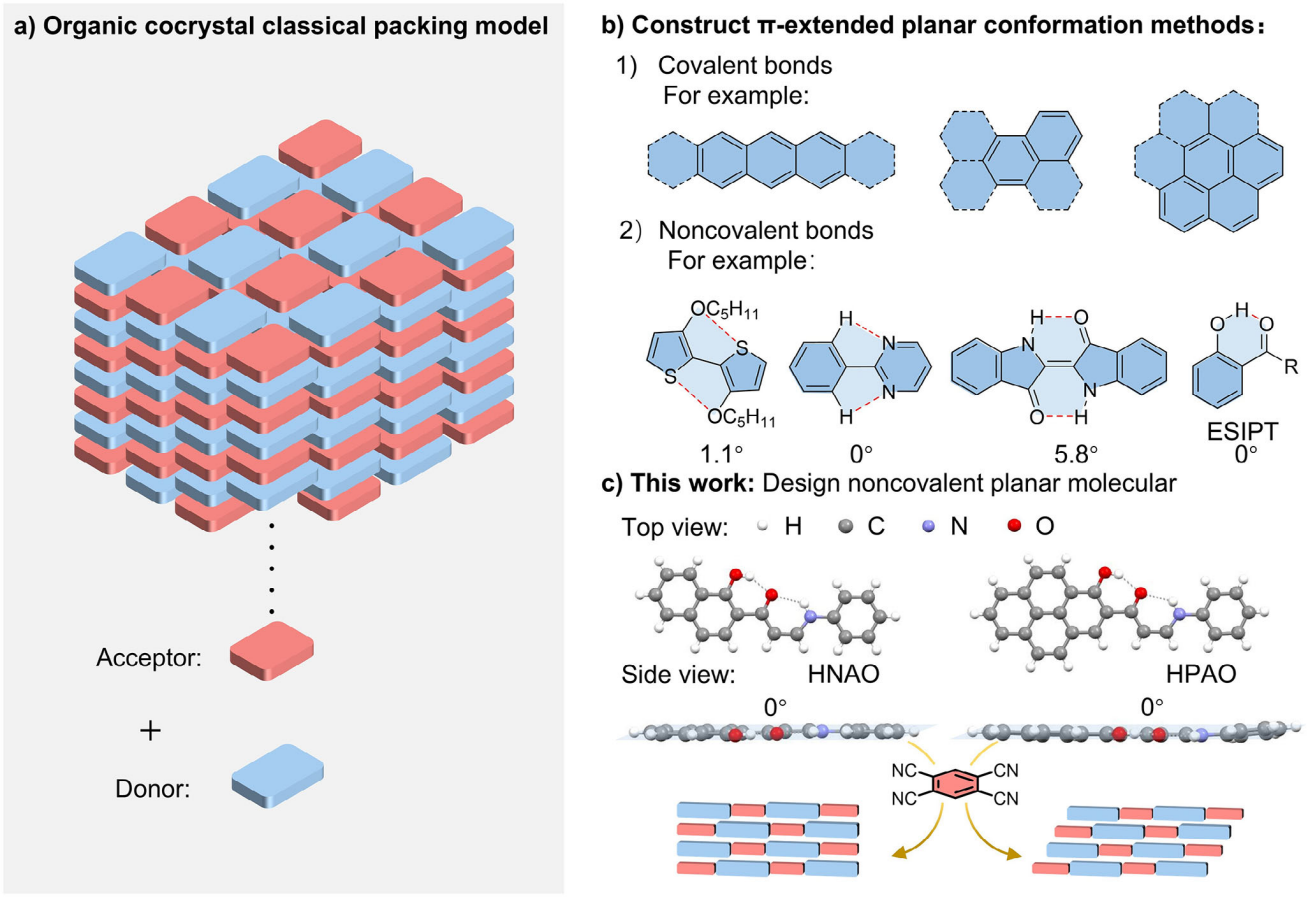
a) Diagram of a classical packing model for an organic cocrystal. b) Overview of the methods for constructing π‐extended planar conformations. c) This work focuses on the design of noncovalent planar molecules for cocrystal engineering.

Organic cocrystals, celebrated for their ease of preparation and unique photophysical and optoelectronic properties, have attracted significant attention in the fields of materials chemistry, bioengineering, and aggregate science.^[^ ^1^, ^2^, ^3^, ^4^, ^5^ ^]^ These materials exhibit a range of functionalities that can be tailored for various applications, such as multi‐stimuli responsive materials,^[^ ^6^, ^9^ ^]^ nonlinear optical materials,^[^ ^10^, ^11^ ^]^ electronic devices,^[^ ^12^, ^13^, ^14^ ^]^ and cell imaging,^[^ ^15^, ^16^ ^]^ making them a focal point in materials science. Typically, organic cocrystals are assembled through various non‐covalent interactions, including CT, *π–π* stacking, halogen‐bonding, and hydrogen‐bonding, of which CT interactions were most common and were widely regarded as the primary driving force behind cocrystal formation.^[^ ^2^, ^3^, ^4^, ^5^ ^]^ To enhance these CT interactions and thereby increase the likelihood of cocrystal formation, π‐conjugated (hetero)aromatic compounds with high planarity and rigidity were often employed as donors or acceptors (**Figure** **1**a).^[^ ^2^, ^3^, ^4^, ^5^, ^17^ ^]^ However, the limited variety of raw materials impedes the advancement of cocrystal engineering.

In contrast to traditional large π‐conjugated systems, non‐conjugated planar molecules locked by weak interactions offer several advantages, including broader chemical diversity, simpler synthetic routes, and greater tunability in cocrystal engineering (Figure 1b).^[^ ^18^ ^]^ Conventional approaches to constructing π‐extended planar conformations often rely on covalently linking adjacent aromatic rings or introducing substituents into conjugated aromatic systems, but these strategies remain relatively limited in scope. Recently, conformation‐locking methods that utilize weak interactions such as intramolecular hydrogen‐bonding or S···X interactions have been widely adopted to produce planar, rigid molecules with enhanced properties in organic photovoltaic materials.^[^ ^19^, ^20^ ^]^ Particularly, these rigid planar structures effectively suppress nonradiative decay, leading to higher fluorescence efficiency and improved photothermal stability. In addition, they lower the reorganization energy and promote orderly molecular packing, which leads to red‐shifted absorption. This approach not only expands the range of available raw materials but also enables precise tailoring of molecular attributes in a broader chemical space, opening new avenues for advanced applications in optoelectronics and photonics.

Among various noncovalent conformation lock strategies, electrostatic hydrogen‐bonding is particularly crucial in defining molecular conformation and photophysical characteristics.^[^ ^21^ ^]^ When an intramolecular hydrogen bond is formed within five‐ or six‐membered rings between a hydrogen bond donor (─OH and ─NH_2_) and a hydrogen bond acceptor (═N─ and C═O), the molecule adopts a relatively planar conformation and undergoes excited state intramolecular proton transfer (ESIPT) progress upon photoexcitation.^[^ ^22^, ^23^ ^]^ This ESIPT progress involves enol/keto phototautomerization, leading to a narrow emission band and possessing a large Stokes shift, which limits reabsorption processes and subsequent inner filter effects, thereby enabling the successful application of ESIPT molecular materials in fluorescent probes, optoelectronic devices, and laser dyes.^[^ ^24^, ^25^, ^26^ ^]^ Typically, ESIPT induces a redistribution of π‐bonds, accompanied by a weakening of C═C double bonds in the excited state. In solution, this process often leads to *cis*–*trans* isomerization and non‐radiative deactivation following proton transfer, as observed in N–H–based ESIPT systems. However, in the solid state, the restriction of large‐scale atomic motion significantly restricts *cis*–*trans* isomerization and non‐radiative deactivation and preserving the ESIPT and two‐photon absorption (TPA) properties of the monomers, and enhances fluorescence quantum efficiency. Furthermore, ESIPT molecules are often equipped with highly conjugated π–bond structures, making them suitable for designing TPA materials.^[^ ^27^ ^]^ By using intermolecular hydrogen bonds to lock and stabilize the critical planar configuration of the donor molecules,^[^ ^28^ ^]^ CT cocrystal can effectively preserve the monomers’ ESIPT and TPA properties, provided these structural features remain unaltered during co‐assembly. This strategy motivates us to incorporate ESIPT‐derived planar conjugated scaffolds into cocrystal assemblies, thereby broadening the library of precursor molecules for cocrystal formation.

Herein, we developed two hydrogen‐bonded interlocked planar molecules,^[^ ^29^, ^30^ ^]^ (*Z*)‐1‐(1‐hydroxynaphthalen‐2‐yl)‐3‐(phenylamino)prop‐2‐en‐1‐one (HNAO) and (*Z*)‐1‐(1‐hydroxypyren‐2‐yl)‐3‐(phenylamino)prop‐2‐en‐1‐one (HPAO), which were successfully combined with acceptor 1,2,4,5‐tetracyanobenzene (TCB) to assembly two CT cocrystals: NTC and PTC, respectively (Figure 1c). X‐ray diffraction (XRD) confirmed that both HNAO and HPAO exhibited excellent planarity in their single crystals and the corresponding cocrystals. Their photophysical properties were further investigated; temperature‐dependent photoluminescence, transient absorption (TA) spectra, and density functional theory (DFT) calculations were employed. For HNAO, the ESIPT process leads to the formation of a keto form with a bathochromic shift, while also endowing HNAO with TPA properties. The NTC cocrystal preserves these features, with the bathochromic shift further enhanced by CT excited states, ultimately achieving near‐infrared (NIR) emission. Additionally, the NTC organic microwires exhibited exceptional crystallinity and an impressively low optical‐loss coefficient of 0.021 dB/µm, making them ideal for optical waveguidance applications. In contrast, HPAO lacks ESIPT behavior in its pure form or the PTC system. Instead, the minimal energy gap between the HOMO of HPAO and the LUMO of TCB in PTC promotes a significantly higher degree of CT in its excited states, which predominantly facilitates non‐radiative transitions, leading to non‐emissive properties. This trade‐off enhances the photothermal performance of PTC, achieving highly efficient photothermal conversion (η = 47.7%) and remarkable photostability. These attributes make PTC particularly effective for applications such as rapid photothermal imaging and time‐dependent information encryption. This work introduces planar molecules constructed through intramolecular hydrogen‐bonding interactions as cocrystal precursors, moving beyond the conventional use of large π‐conjugated aromatic compounds and expanding the cocrystal materials library. Furthermore, the optical, physical, and photothermal properties of these planar molecules can be precisely tailored for the creation of cocrystals with specialized characteristics.

## 2 Results and Discussion

### 2.1 Synthesis and Characterization of HNAO and HPAO

The synthetic routes toward HNAO and HPAO were shown in the Supporting Information, and both compounds were fully characterized by using ^1^H and ^13^C NMR. HNAO was synthesized starting with the reaction of 1‐(1‐hydroxynaphthalen‐2‐yl)ethan‐1‐one with N, N‐dimethylformamide dimethylacetal to obtain an enamine intermediate. This enamine then underwent dehydration condensation and tautomerization with aniline, yielding HNAO in an eco‐friendly aqueous solution. Similarly, 1‐(1‐hydroxy‐3*a*1,5*a*1‐dihydropyren‐2‐yl)ethan‐1‐one was used as the starting material to prepare enaminone HPAO via the same method (experiment details in Supporting Information).

To investigate the impact of intramolecular hydrogen bonds on molecular conformation, single‐crystal XRD analysis was performed on both HNAO and HPAO (Figures S1 and S2 and Table S1, Supporting Information). In their crystalline states, both compounds exhibit a herringbone packing model with two types of intramolecular hydrogen bonds contributing to enhanced molecular planarity and rigidity, resulting in a dihedral angle of 0°. The first type involves the hydroxyl group and the carbonyl oxygen atom (C = O···H−O), with bond distances of 1.754 Å for HNAO and 1.765 Å for HPAO. The second type involves the amino hydrogen group and the carbonyl oxygen atom (C = O···H−N), with bond distances of 2.015 Å for HNAO and 2.012 Å for HPAO. Structurally, HNAO adopts a monoclinic structure with unit cell parameters of *a* = 13.989 Å, *b* = 5.427 Å, *c* = 18.416 Å, *α* = 90°, *β* = 93.8°, *γ* = 90°, and belongs to the space group *P*2_1_/*n*. HPAO crystallizes in a triclinic structure with parameters *a* = 8.064 Å, *b* = 13.857 Å, *c* = 15.768 Å, *α* = 78.4°, *β* = 83.2°, *γ* = 89.7°, and belongs to the space group *P*‐1. Moreover, the crystal structure of HNAO showed a slip‐stacking arrangement, indicating that *π–π* interactions play a relatively minor role in its overall crystal structure. In contrast, HPAO exhibits a head‐to‐tail stacking arrangement dominated by strong *π–π* interactions. These differences highlight the distinct packing and interaction modes of the two molecules.

To further understand their photophysical properties, the absorption and emission spectra were measured in both solution and solid states (Figures S3 and S4, Supporting Information). The maximum emission peak of HNAO in the solid state is 545 nm, while HPAO exhibits non‐emission behavior. Notably, HNAO demonstrated significant aggregation‐induced emission (AIE) behavior, with a 3.2‐fold enhancement in fluorescence intensity observed in a 99% water fraction (Figure S5, Supporting Information). Moreover, the emission band experienced a slight redshift as the water fraction increased up to 70%, which can be attributed to the formation of J‐aggregates that induce red‐shifted emission.^[^ ^31^ ^]^ Conversely, HPAO displayed aggregation‐induced quenching, likely due to the strong molecular *π–π* stacking during H‐aggregate (Figure S6, Supporting Information).^[^ ^32^, ^33^ ^]^

### 2.2 Cocrystal Design and Structure Analysis

**Figure 2:**
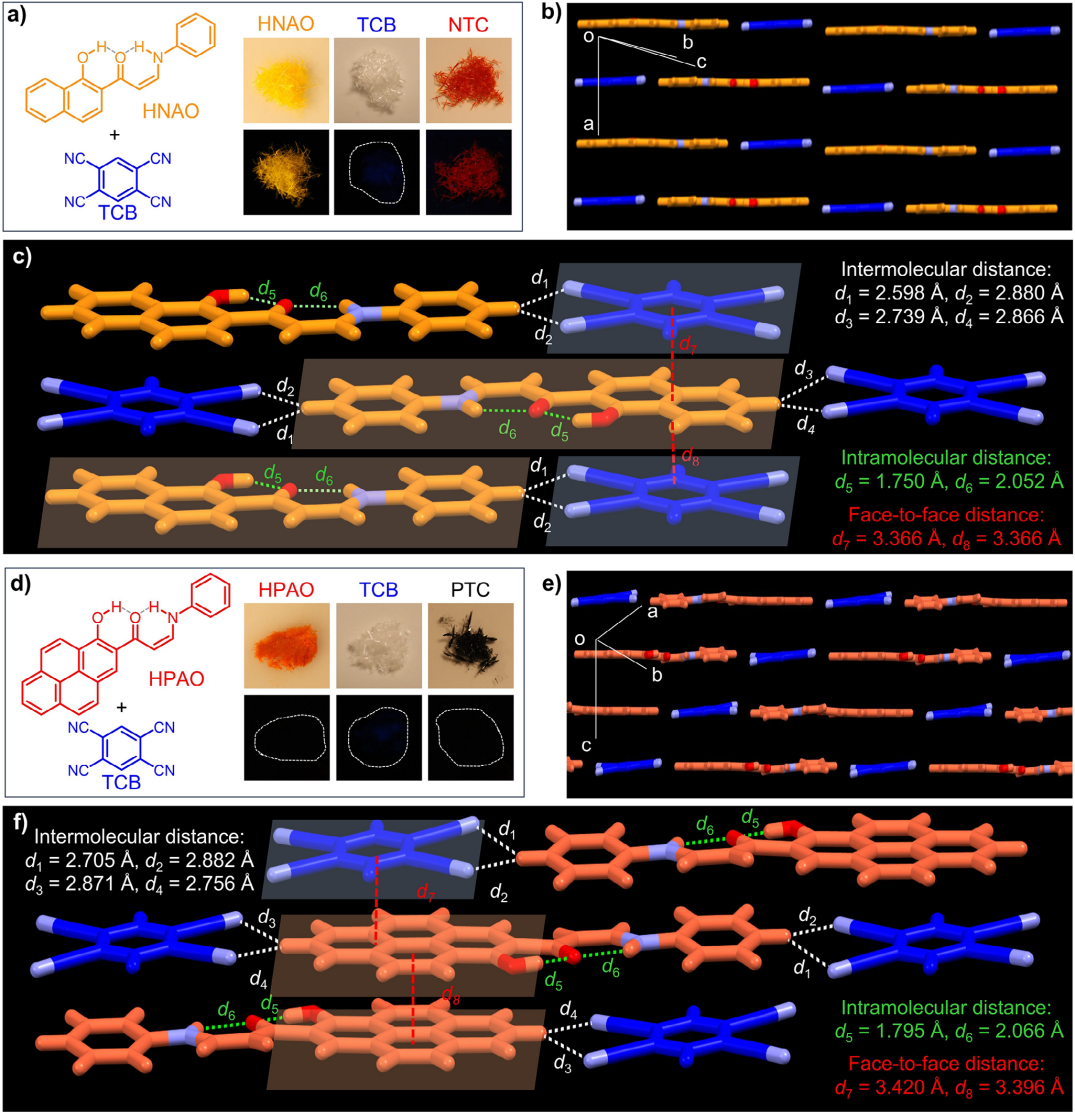
a) Molecular structures of the NTC cocrystal, along with photographs of HNAO, TCB, and the NTC cocrystal under room light (up) and a 365 nm UV lamp (down); b) Crystal structure and c) Noncovalent interactions in NTC. d) Molecular structures of the PTC cocrystal, with photographs of HPAO, TCB, and the PTC cocrystal under room light (up) and a 365 nm UV lamp (down). e) Crystal structure and f) Noncovalent interactions in PTC.

To demonstrate the potential of extended π‐electron molecules constructed through non‐covalent bonds as ideal candidates for cocrystal assembly due to their perfect planarity and favorable rigidity, the inherent electron‐rich HNAO and HPAO were selected as donor components to assemble with the electron acceptor TCB for the construction of CT cocrystals. Fortunately, the HNAO/TCB cocrystal (NTC) was obtained with a 1:1 molar ratio by slow vapor diffusion of hexane into a THF solution of HNAO and TCB (Figure 2a). Similarly, HPAO/TCB cocrystal (PTC) with a molar ratio of 1:1 was obtained using the same method (Figure 2d). As shown in Figure 2b,e, both NTC and PTC cocrystals exhibit a mixed‐stack structure, in which the donors and acceptors maintain excellent planarity, allowing for face‐to‐face stacking along the *a*‐axis and *c*‐axis, respectively. Specifically, HNAO and TCB form head‐to‐tail A–D–A columns along the *a*‐axis with measurable donor–acceptor overlap in NTC, whereas HPAO and TCB exhibit a less continuous A–D alignment along the *c*‐axis with intermittent D–D adjacency segments and reduced effective D–A overlap in PTC. Notably, the individual HNAO and HPAO components retain their excellent planarity within the cocrystal framework, demonstrating that the construction of non‐covalent molecular planarity for cocrystal engineering is both reasonable and achievable. This advancement extends beyond the previous limitation of exclusively selecting conjugated aromatic/heteroaromatic hydrocarbons imposed by planarity constraints, opening new possibilities for cocrystal engineering. XRD analysis of NTC revealed that intramolecular hydrogen‐bonding interactions (C═O···H─O and C═O···H─N) in monomer HNAO are consistent with those in the HNAO single crystal and play a crucial role in maintaining its planar configuration. The monomer molecules HNAO and TCB in NTC also exhibit face‐to‐face stacking, stabilized by intermolecular hydrogen‐bonding interactions (CH···N, 2.598–2.880 Å) and *π–π* interactions (3.366 Å) between the donor and acceptor (Figure 2c; Table S2, Supporting Information). This strong intermolecular hydrogen‐bonding and *π–π* interactions between HNAO and TCB facilitated the effective CT interaction. Meanwhile, multiple non‐covalent hydrogen bonds, including intramolecular hydrogen‐bonding interactions (C═O···H−O, 1.795 Å and C═O···H−N, 2.066 Å), as well as intermolecular hydrogen‐bonding interactions (CH···N, 2.705–2.882 Å), were observed in PTC, which significantly contribute to a tightly packed CT complex. Moreover, the intermolecular D−A distance (3.420 Å) indicates a strong intermolecular CT interaction (Figure 2f). By comparing NTC and PTC, it is evident that both cocrystals share a common structural stabilization mechanism, wherein intramolecular hydrogen bonds lock the planarity of HNAO and HPAO, and intermolecular hydrogen‐bonding and *π–π* interactions further restrict molecular motion and promote CT interactions.

### 2.3 Photophysical Properties of Cocrystal

**Figure 3:**
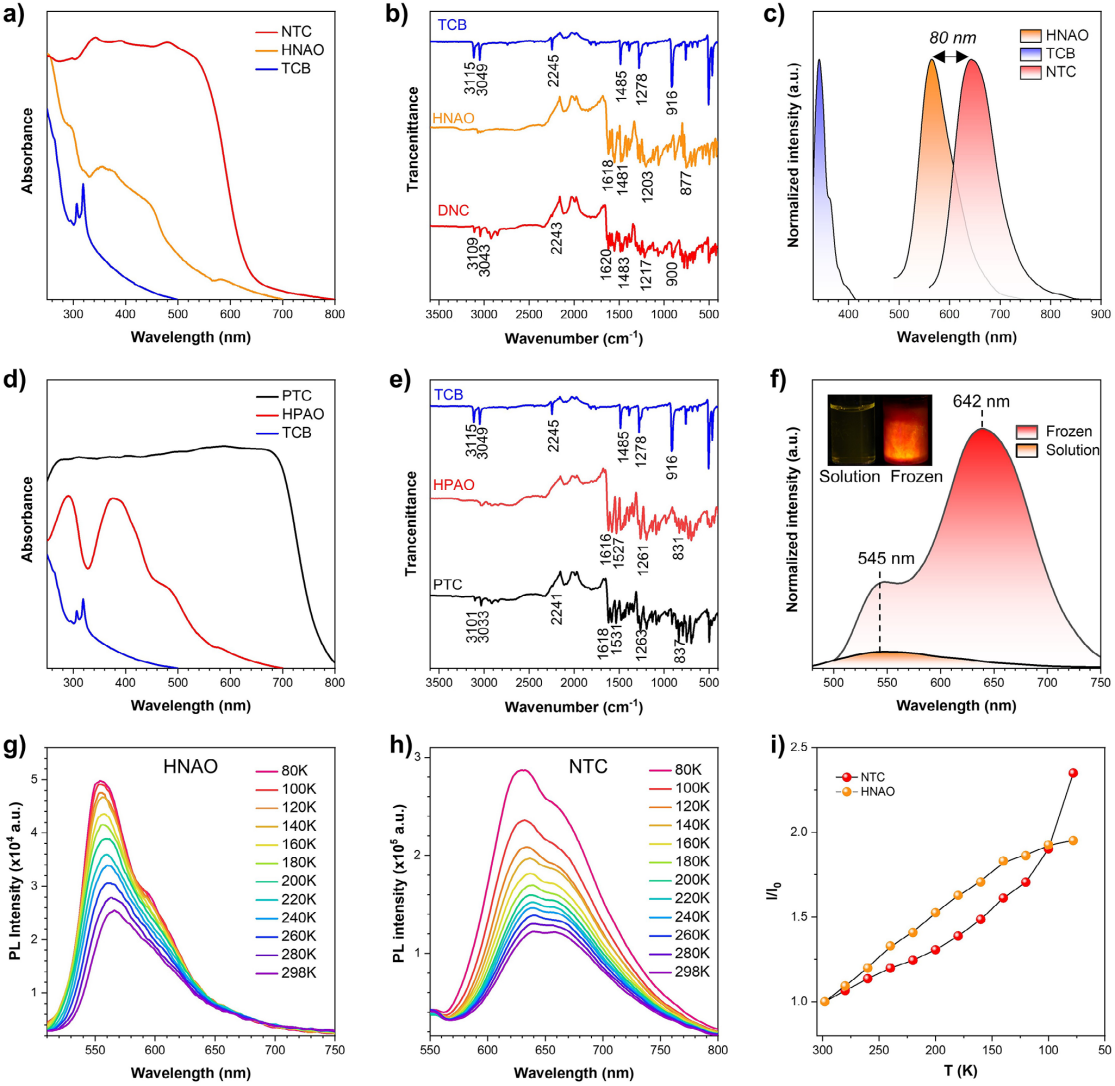
Noncovalent interactions in the single‐crystal structure of DNTC. Absorption spectra of a) NTC, HNAO, and TCB, and d) PTC, HPAO, and TCB. FTIR spectra of b) TCB, HNAO, and NTC, and e) TCB, HPAO, and PTC. c) PL spectra of HNAO, TCB, and NTC in the solid state. f) PL spectra of NTC in solution and frozen state in acetonitrile. Inset: fluorescence images of NTC in acetonitrile at room temperature and 77 K in a frozen state. c = 1 × 10^−5^ M, *λ*_ex_ = 405 nm. Temperature‐dependent PL spectra of g) HNAO and h) NTC during heating from 80 to 298 K with excitation wavelengths at 380 and 405 nm, respectively. i) Plots of relative PL intensity (*I*/*I*_0_) of temperature‐dependent PL spectra. *I*/*I*_0_: ratio between the maximum emission intensity at different temperatures and the maximum emission intensity at room temperature.

The NTC and PTC cocrystals showed a broad red‐shifted absorption compared to their components, indicating the formation of a strong ground‐state CT complex (Figure 3a,d). When comparing the Fourier transform infrared spectroscopy (FTIR) of the cocrystals with those of the individual compounds, a noticeable shift in stretching frequencies was observed. Specifically, the C─H stretching bands at 3115 and 3049 cm^−1^ in the TCB single crystal shifted to 3109 and 3043 cm^−1^ in the cocrystal, while the C═C stretching band shifted from 1485 to 1483 cm^−1^, indicating an increase in electron density within the benzene ring of the TCB component and confirming the formation of CT complexes (Figure 3b).^[^ ^34^ ^]^ A similar shift was observed in the FTIR spectra of PTC (Figure 3e), reinforcing the presence of strong intermolecular CT interactions. Moreover, upon cocrystal formation, the C≡N stretching band (2245 cm^−1^) shifts to lower wavenumbers (2243 cm^−1^ in NTC, 2241 cm^−1^ in PTC). This shift is consistent with intermolecular hydrogen bonding (C─H···N≡C) to the nitrile nitrogen and possible partial charge transfer in the donor–acceptor pairs, which weakens and slightly lengthens the C≡N bond, thereby lowering its bond order.

To further explore their optical properties, photoluminescence (PL) spectroscopy was employed. In their single‐component crystalline forms, TCB and HNAO exhibited blue and yellow fluorescence, with maximum emission peaks at 342 and 565 nm, respectively. Upon cocrystallization, NTC cocrystal exhibited an 80 nm red shift compared to HNAO, resulting in deep‐red emission with a maximum at 645 nm and a tail extending to 750 nm, thereby reaching into the NIR‐I region (650–950 nm)^[^ ^35^ ^]^ (Figure 3c) and a fluorescence quantum yield (*Ф*_QY_) of 2.1%. To investigate the emission behavior in the aggregate state, NTC was dissolved in acetonitrile and then rapidly frozen at 77 K using liquid nitrogen. Under UV irradiation, a strong red emission, accompanied by a weaker yellow emission at specific spots, was observed, corresponding to the cocrystal (642 nm) and HNAO (545 nm), respectively (Figure 3f). Upon rapid cooling, the acetonitrile solution partially solidifies. The drop in solubility drives co‐assembly of HNAO (D) and TCB (A) into a cocrystal. The red emission originates from this CT cocrystal, whereas the yellow emission arises from the HNAO molecule (Figure S7, Supporting Information). These findings confirm crystallization‐induced emission enhancement behavior, demonstrating the AIE properties of the NTC cocrystal,^[^ ^14^, ^36^ ^]^ which is consistent with HNAO. Moreover, PTC exhibited non‐emission behavior under UV irradiation (Figure S8, Supporting Information), which is in line with the non‐emissive behavior of HPAO. This observation suggests that the luminescent behavior of the cocrystal aligns with that of its donor components, the hydrogen‐bonded interlocked planar conformation molecules.

To further elucidate the luminescence mechanism of HNAO and NTC, temperature‐dependent photoluminescence behavior was investigated by cooling the samples from 298 to 80 K in 10 K intervals. For typical ESIPT molecules, as the temperature decreases, molecular motion becomes restricted, suppressing nonradiative relaxation processes. However, when the temperature drops below a critical level, proton transfer is inhibited, leading to a decrease in emission intensity.^[^ ^37^ ^]^ For HNAO, the emission intensity increased steadily from 298 to 140 K, followed by a gradual plateau from 140 to 80 K, which can be attributed to the slowing of the proton transfer process as the temperature drops below a certain threshold. In contrast, NTC exhibited a continuous increase in PL intensity as the temperature decreased, with a particularly notable enhancement at ultra‐low temperatures (120 to 80 K), in stark contrast to HNAO. These results suggest that while the luminescence of HNAO is predominantly governed by ESIPT dynamics, the pronounced increase in emission intensity of NTC upon decreasing temperature implies that its luminescent mechanism is not entirely governed by ESIPT, warranting further investigation.

### 2.4 TA Spectra and ESIPT Process of Monomer and Cocrystal

**Figure 4:**
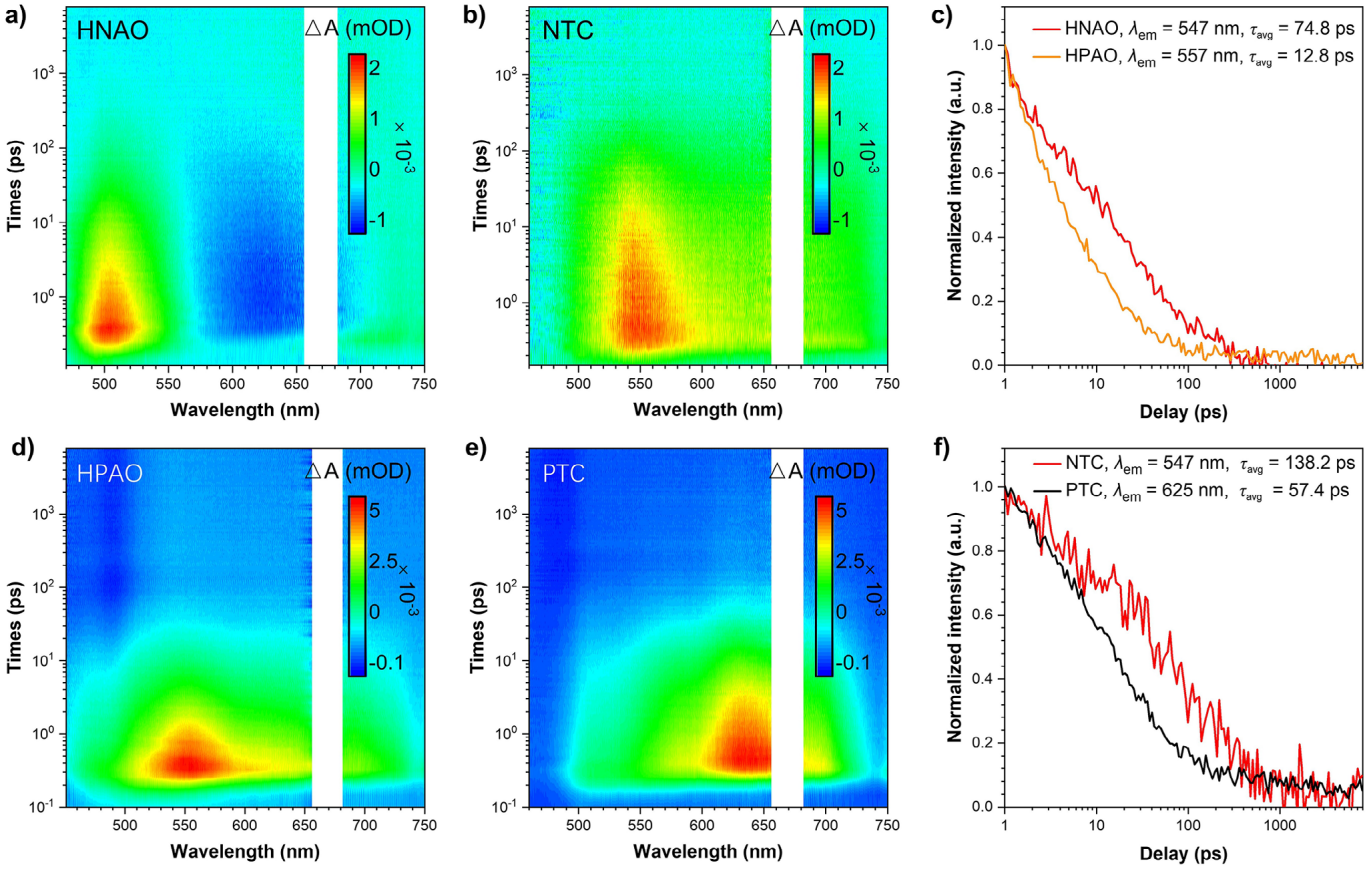
Color plot of the TA spectra of a) HNAO, b) NTC, d) HPAO, and e) PTC. The single wavelength kinetics of the excited state: c) HNAO at 547 nm and HPAO at 557 nm, f) NTC at 547 nm and PTC at 625 nm as a function of time.

To further understand the excited‐state kinetics, TA measurements were conducted. The TA spectrum of HNAO exhibits an excited‐state absorption (ESA) signal at 450–550 nm, along with a stimulated emission (SE) at 550–700 nm (Figure 4a), which closely aligns with the emission wavelength range of its steady‐state fluorescence spectrum. Due to its large Stokes‐shifted emission (200 nm) (Figure S3, Supporting Information), which indicates that HNAO undergoes the ESIPT process upon photoexcitation. Moreover, DFT calculation at CAM‐B3LYP/6‐31G(d,p) level further confirmed this behavior, revealing that the proton transfer process (Enol^*^→Keto^*^) occurs without an energy barrier (Δ*G* = ‐7.5 kcal/mol), suggesting a smooth ESIPT process (Figure S9, Supporting Information). However, the TA spectrum of the NTC cocrystal displays a broader ESA band spanning 450–750 nm (Figure 4b), which differs significantly from that of HNAO. DFT calculations indicate that the ESIPT process (Enol^*^→Keto^*^) remains barrierless in NTC (Δ*G* = −13.7 kcal/mol), confirming that HNAO readily undergoes ESIPT, converts to the keto form upon excitation, and subsequently forms a CT complex with TCB. Furthermore, the TA feature of HPAO exhibits a broad ESA feature at 450–750 nm with a peak at 557 nm, and no SE features, which is consistent with its steady‐state PL spectrum (Figure 4d; Figure S4, Supporting Information). The substantial difference in the TA features between HPAO and HNAO strongly suggests that HPAO does not undergo ESIPT in its monomeric crystal form. Similarly, its cocrystal, PTC, formed with TCB, shows an ESA feature from 500 to 750 nm, peaking at 625 nm, also demonstrating no ESIPT progress (Figure 4e). These TA features also exhibit significant differences between individuals and cocrystal suggests that there are large differences in the electronic energy levels between the individuals and their cocrystals, and the electronic energy levels of the cocrystals are mainly attributed to the CT states induced by the D‐A complex.

To gain deeper insights into the excited‐state dynamics, the kinetic profiles were extracted. The excited‐state lifetimes (τ_avg_) of HNAO and HPAO were measured to be 74.8 and 12.8 ps, while NTC and PTC were 138.2 and 57.4 ps, respectively (Figure 4c,f; Table S3, Supporting Information). Given that the non‐emission behavior and *τ*_avg_ of HPAO and PTC are much shorter than those of HNAO and NTC, which undergo the ESIPT process, the non‐radiative decay constants (*k*_nr_) for HNAO and NTC were calculated using the formula: *k*_nr_ = (1‐*Ф*_QY_)/*τ*_avg_ (*Ф*_QY_ = 0), yielding values of 10.4 and 7.1 ns^−1^, respectively. In contrast, the *k*_nr_ of HPAO and PTC are 78.1 and 17.4 ns^−1^, respectively, much larger than those of HNAO and NTC (Table S4, Supporting Information). These results further indicated that HPAO and PTC undergo faster non‐radiative decay, leading to non‐emissive properties.^[^ ^38^, ^39^ ^]^

### 2.5 Theoretical Calculation on the Mechanism of Cocrystal Formation

**Figure 5:**
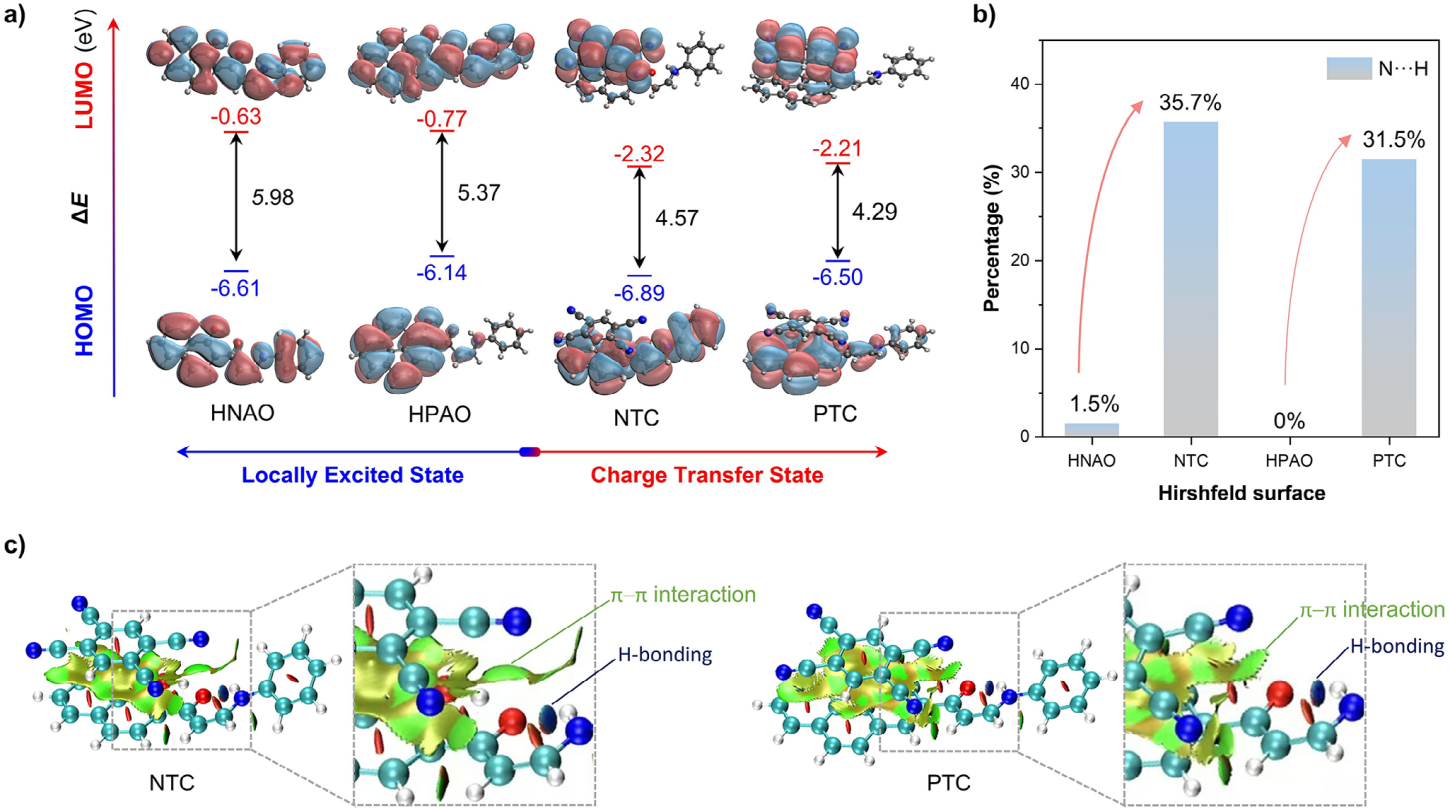
a) Frontier molecular orbitals are calculated from the optimized ground state via the time‐dependent density functional theory method at the CAM‐B3LYP/6‐31G(d,p) level. b) Hirshfeld surface analysis plots (mapped over *d*_norm_) of N···H. c) NCI surfaces of the NTC and PTC.

To gain a deeper understanding of the luminescent phenomena and mechanisms, theoretical calculations were employed on both the monomer and the cocrystal. Frontier molecular orbital calculations first revealed that the highest occupied orbitals (HOMO) of NTC (−6.14 eV) and PTC (−6.50 eV) originated from the donor molecule HNAO (−6.14 eV) and HPAO (−6.89 eV), respectively. Meanwhile, the lowest unoccupied molecular orbitals (LUMO) of NTC (−2.32 eV) and PTC (−2.21 eV) were predominantly localized on the acceptor TCB (−3.26 eV) molecule. These results demonstrated that a transition from LE to CT excitation occurs during the transition from the individual molecule to the cocrystal. Moreover, according to the calculated electrostatic potential distribution of the monomers and two cocrystals, the TCB part, appearing bluer, indicates a lower electronic density and a tendency to attract electrons, whereas the HNAO or HPAO part appear redder, signifying higher electron density and a tendency to repel electrons, thereby facilitating intermolecular CT interactions (Figure S10, Supporting Information). Additionally, the intermolecular CT excitations of NTC (99.6%) and PTC (99.2%) were confirmed through natural transition orbital analysis (Figure S11, Supporting Information). Hirshfeld surface analysis is crucial for understanding packing interactions and quantifying molecular contributions and has become an important means for exploring intermolecular interactions. The proportion of N···H interactions dramatically increased, rising from 1.5% to 35.7% between HNAO and NTC and from 0.0% to 31.5% between HPAO and PTC, as summarized from 2D fingerprint plots for both monomeric and cocrystalline systems (Figure 5b; Figures S12–S16, Supporting Information). This increase in N···H interactions is attributed to the addition of TCB molecules, where N atoms from the cyano groups of TCB form intermolecular N···H interactions with H atoms from donor molecules, effectively restricting molecular motion and locking the adjacent donor and acceptor molecules in the same plane.

To further investigate the nature and efficiency of intermolecular interactions, a reduced density gradient analysis based on the noncovalent interaction index (NCI) was performed. The NCI surfaces represent different types of interactions: van der Waals interactions (green), attractive force (blue), and steric repulsion (red). The presence of green regions between the donor and acceptor parts in NTC and PTC indicates weak *π–π* interactions. Interestingly, these van der Waals interactions suggest π‐extension beyond the aromatic rings, extending to the ketone moieties, and even appearing in the noncovalent bond planar regions. Meanwhile, the blue regions correspond to hydrogen bonds between C═O···H─N in both NTC and PTC, confirming strong intramolecular hydrogen bonds in their corresponding donor molecules (Figure 5c). Thus, these results further support that intramolecular hydrogen bonds stabilize the planarity of HNAO and HPAO in both NTC and PTC cocrystals, while intermolecular *π–π* interactions restrict molecular motion and promote CT interactions.

### 2.6 TPA Properties of HNAO and NTC

**Figure 6:**
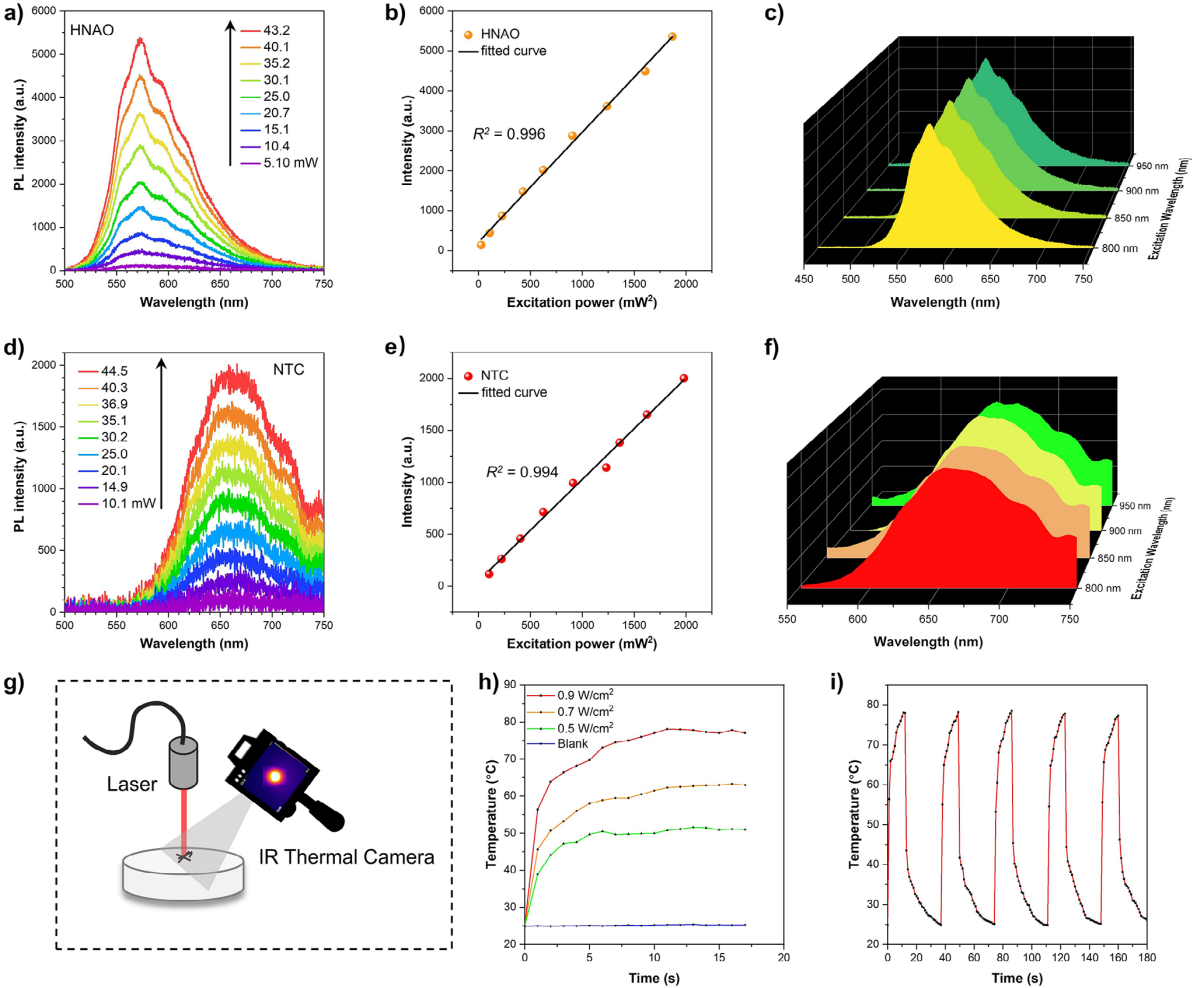
The PL spectra of a) HNAO and d) NTC under laser excitation, λ_ex_ = 800 nm. b) HNAO's and e) NTC's corresponding linear relation between the PL intensity and excitation power. The PL spectra of c) HNAO and f) NTC under different excitation laser wavelengths. All the excitation sources are femtosecond lasers. g) Diagram of the photothermal conversion measurement. h) Photothermal conversion curves of cocrystals under 660 nm laser irradiation of different power densities. i) Temperature evolutions of PTC before and after 5 heating/cooling cycles under 660 nm laser irradiation. laser power = 0.9 W/cm^2^.

In previous studies, solid‐state ESIPT molecules have been explored for designing TPA materials, which represent a third‐order nonlinear optical process.^[^ ^5^, ^7^, ^8^ ^]^ To investigate the TPA properties of HNAO, long‐wavelength excited fluorescence measurements were performed. Upon excitation with an 800 nm femtosecond laser, the upconversion PL spectra of HNAO crystals exhibited the same spectral shape across different laser power levels in the steady‐state measurement (Figure 6a), demonstrating that the emission processes from both one‐ and two‐photon excited states to the ground state are identical. As the excitation power increased, the two‐photon excited fluorescence intensity showed a quadratic dependence on the incident energy, confirming the TPA process (Figure 6b). Moreover, HNAO exhibited a broad two‐photon excitation window ranging from 800 to 1000 nm (Figure 6c).

Such exciting results encouraged us to investigate the TPA properties of NTC to explore strategies for predicting cocrystal properties by maintaining monomeric functional configuration^.[^ ^7^, ^10^, ^11^ ^]^ Remarkably, NTC exhibited two‐photon excited fluorescence with a maximum emission band range from 655 to 680 nm, which closely matches its one‐photon excited fluorescence (Figure 6d). Moreover, NTC exhibits a linear relationship between fluorescence intensity and the square of incident laser power (Figure 6e) and a broad TPA spectrum extending from 800 to 1000 nm (Figure 6f). Furthermore, two‐photon images of HNAO and NTC were collected using a state‐of‐the‐art confocal laser scanning microscope. As the laser wavelength increased from 800 to 1000 nm in 50 nm increments, stronger laser power was required to maintain luminescence in both HNAO and NTC systems. With the laser power fixed, fluorescence intensity visibly decreased and eventually disappeared as the excitation wavelength increased from 800 to 1000 nm in both HNAO and NTC systems (Figure S17, Supporting Information). Additionally, when the excitation wavelength was fixed at 800 nm, the fluorescence intensity of both HNAO and NTC displays a quadratic dependence on the laser power. Therefore, the TPA properties of NTC can be effectively preserved by retaining the functional configuration of monomeric HNAO (Figure S18, Supporting Information). Furthermore, the intermolecular CT interactions between electron donors and acceptors in the cocrystal system contribute to the redshift of the TPA spectrum compared to the individual monomer.

### 2.7 Photothermal Conversion Properties of HPAO and PTC

Photothermal conversion based on CT organic cocrystals, which can absorb light and convert it into heat, has gained significant attention due to strong CT interactions, which narrow the energy bandgap and enhance light‐harvesting properties compared to their constituent components.^[^ ^34^, ^40^ ^]^ However, the development of photothermal cocrystal materials has been challenged by the difficulty of selecting D‐A components that match electron distribution and structure, the unpredictability of crystallization self‐assembly, and uncertainties regarding their photothermal conversion properties. The non‐emissive behavior of HPAO molecules led us to investigate whether the absorbed light energy was primarily converted into heat. Indeed, the temperature of HPAO powders increased rapidly, reaching 54 °C within 40s under 405 nm laser irradiation, as monitored by an IR thermal camera (Figure 6g; Figure S19, Supporting Information). In contrast, the temperature in the blank experiment increased by only 1 °C. Moreover, the photothermal stability of HPAO was evaluated by comparing its absorption spectra and powder X‐ray diffraction (PXRD) before and after multiple heating and cooling cycles. While the absorption spectra remain consistent, indicating the photochemical stability of the HPAO complex, inconsistencies in the PXRD patterns suggest structural collapse of the crystals (Figure S20, Supporting Information). Encouraged by the excellent photothermal results of HPAO, the photothermal properties of their corresponding cocrystal, PTC, were further examined. Given that PTC exhibits an efficient absorption region spanning 300–700 nm, a 660 nm laser was selected to evaluate its NIR photothermal conversion performance. Notably, the temperature of PTC solid powder, as recorded by an IR thermal camera, exhibited a substantial increase, confirming the strong photothermal properties of the cocrystal. As shown in Figure 6h, the temperature increment of PTC exhibits a power density‐dependent photothermal effect under 660 nm laser irradiation. Additionally, the temperature rise follows a consistent trend: a rapid increase within the initial 5 s, a gradual rise until ≈ 10 s, and then stabilization at a constant temperature. After five cycles of heating and cooling, the PXRD and absorption spectra of PTC remained consistent with their original states, indicating excellent photothermal stability and superior structural stability compared to the HPAO single crystal (Figure 6i; Figure S21, Supporting Information). By decreasing the HPAO: TCB ratio from 1:1 to 1:10, the photothermal effect under 660 nm excitation decreased markedly, suggesting that the 660 nm–induced photothermal effect is unique to PTC (Figure S22, Supporting Information). Furthermore, the photothermal conversion efficiency of PTC was calculated to be 47.7% based on the cooling curve (for details, see Supporting Information), which is above the average value reported for photothermal materials (Figures S23 and S24, Supporting Information).^[^ ^39^ ^]^

To gain deeper insights into the correlation between photothermal properties and CT interactions, the degree of charge transfer (DCT) was calculated to quantify the extent of charge redistribution between donor and acceptor molecules in the cocrystal system.^[^ ^39^ ^]^ The DCT can be estimated by analyzing bond length variations in single‐crystal XRD or wavenumber shifts in IR spectroscopy.^[^ ^41^, ^42^ ^]^ From a bond‐length perspective, the C─H bond in TCB is shorter in the cocrystal than in pure TCB, but longer than in the TCB anion. Meanwhile, the C≡N bond in TCB is elongated in the cocrystal compared to pure TCB, but shorter than in the TCB anion, which is consistent with previous work^[^ ^43^, ^44^ ^]^ (Table S5, Supporting Information). These findings indicate that the electron density on TCB in the cocrystal lies between that of its neutral and anion forms, confirming partial CT from donor to acceptor.

From an IR spectroscopy perspective, the DCT is estimated using the following equation:^[^ ^44^, ^45^ ^]^

(1) $$\mathrm{DCT}=2\Delta \;\nu /\nu_{0}{\left(1-{\nu_{1}}^{2}/{\nu_{0}}^{2}\right)}^{-1}$$

where Δ*v* = *v*_0_ − *v*_CT_, and *v*_0_, *v*_CT_, and *v*_1_ represent the stretching modes of the pure acceptor, CT complex, and acceptor anion, respectively. The DCT calculated from the wavenumber variation of the C≡N vibration is presented in Table 1, showing that PTC (0.0572) has a higher DCT than NTC (0.0278). This suggests that PTC experiences stronger electronic delocalization, making it more difficult to overcome the forbidden electronic transitions from the CT state, thereby favoring nonradiative decay.^[^ ^40^, ^43^ ^]^ Additionally, the *k*_nr_ of PTC is relatively high and promotes nonradiative inactivation upon photoexcitation. Combining these results with the Jablonski diagram provides a clearer understanding of the photothermal process (Figure S25, Supporting Information).^[^ ^34^, ^40^ ^]^ Upon photoexcitation, electronic transitions populate the excited CT states, which subsequently return to the ground state via nonradiative decay mechanisms, including internal conversion and charge dissociation. These nonradiative pathways efficiently convert absorbed light energy into heat, enhancing the photothermal conversion process.

**Table 1:** *ν*_0_, *ν*_CT_, *ν*_1,_ and calculated DCT of NTC and PTC at room temperature.

	*ν*_0_ [cm^−1^]^[^ ^44^ ^]^	*ν*_CT_ [cm^−1^]	*ν*_1_ [cm^−1^]^[^ ^44^ ^]^	Δ*ν* [cm^−1^]	DCT
TCB in NTC	2245	2243	2170	2	0.0278
TCB in PTC	2245	2241	2170	4	0.0572

### 2.8 Optical Waveguide Applications

**Figure 7:**
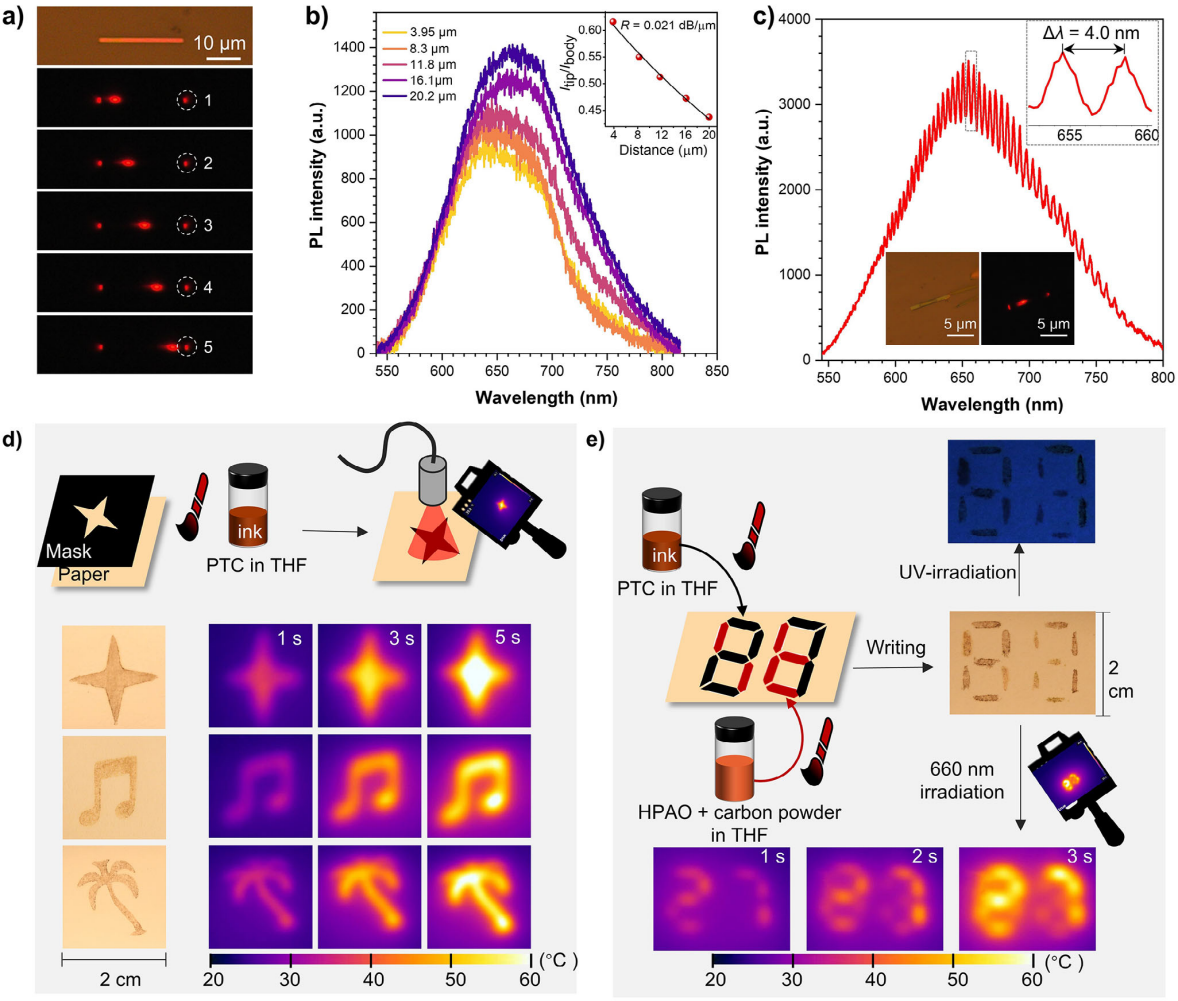
a) Bright‐field microscopy image and PL microscopy images of NTC microwire excited with a focused laser beam at different positions, λ_ex_ = 375 nm. b) Spatially resolved PL spectra from the tip of nanowires for different separation distances between the excitation spot and the tip of the nanowires. Right inset: the curve of the ratio between the PL intensity at the tip and the excitation point versus distance (*I*_body_, the PL intensity at the exciting spots; *I*_tip_, the PL intensity at the left tip). c) PL spectrum of a 25µm‐long microwire. Inset: The bright optical image and fluorescence microscopy images of NTC. d) Diagram of the photothermal imaging process. The PTC was solved in THF solution as the ink for printing. c = 0.5 m, λ_ex_ = 660 nm, laser power = 0.7 W/cm^2^. e) The schematic diagram of the application for information encryption. The PTC was solved in THF solution as the ink for writing the correct information “27”, and the HPAO and carbon powder were solved in THF solution as the ink for writing the other part of the template, the image on the right from top the bottom is under UV irradiation, room light, and thermal camera. c = 0.5 m, λ_ex_ = 660 nm, laser power = 0.7 W/cm^2^.

The 1D morphology NTC cocrystals were fabricated by a microspacing in‐air sublimation method (Figure S26, Supporting Information; the details of fabrication are in the Supporting Information). The hydrogen‐bond interlocked planar rigid molecule HNAO, when co‐assembled with TCB, promotes the formation of high‐quality cocrystal microwires with smooth surfaces, which meet the essential requirements for optical waveguide applications. The resulting cocrystal simultaneously serves as a highly emissive material, making it well‐suited for optical waveguide studies. Subsequently, optical waveguide measurements were performed on NTC microwires with a length of 25 µm, using a fixed laser exciting source (λ_ex_ = 375 nm) at different points along the length of the rod. The sample stage was moved to capture the bright red emission propagating toward the tip using a homemade micro‐area PL spectroscope (Figure S27, Supporting Information). As shown in Figure 7a, the corresponding PL signals, with a peak at 655 nm, exhibit a reduction in intensity as the photon propagation distance (*d*) increases. The calculated ratio of *I*_tip_/*I*_body_ versus the separation distance between the excitation spot and the left tip shows a single exponential decay and can be fitted by the equation:

(2) $$I_{\mathrm{tip}}/I_{\mathrm{body}}=\mathrm{Aexp}\left(-\alpha d\right)$$

The optical loss coefficient *α* for the corresponding nanowire is as low as 0.021 dB/µm, indicating excellent optical confinement, which is essential for integrated photonic applications (Figure 7b). To the best of our knowledge, the optical loss coefficient of NTC is lower than that of previously reported organic cocrystal optical waveguides in both the visible and NIR regions (Figure S28, Supporting Information). The reason might be that the intramolecular hydrogen bonds lock the molecule into a planar and ordered conformation, which in turn promotes regular and stable face‐to‐face molecular packing during the assembly of the CT cocrystal. This structural organization is believed to contribute to the high crystallinity and smooth surface morphology observed in the CT cocrystal, thereby providing a valuable design principle for the development of cocrystals with ultralow optical‐loss coefficients. Furthermore, a series of sharp fluorescence resonance peaks can be observed in the PL spectra of (25 µm in length) at the tip position. As shown in Figure 7c, the resonance peaks, with a peak spacing (Δ*λ*) of 4.0  at 655 nm, demonstrate the microwire's potential for optical signal propagation.^[^ ^45^ ^]^ As compared to traditional inorganic microstructures, organic cocrystal microstructures can be fabricated through a facile self‐assembly solution method at room temperature and exhibit several advantages, such as high luminous efficiency, low cost, and high flexibility in molecular design, wavelength adjustment, and so on.^[^ ^4^, ^5^ ^]^ Consequently, this NIR‐I region emissive NTC microwire, with its smooth surface and high crystallinity, holds great potential as a new building block for photon signal propagation in optical circuits, enabling efficient information communication and storage.

### 2.9 Photothermal Image and Time‐Dependent Information Encryption

Compared with their inorganic metal counterparts, organic semiconductors have made significant progress in photothermal applications, especially organic photothermal cocrystal materials, which benefit from their tailor‐made molecular structures, adjustable optical/electronic properties, and low‐cost, scalable fabrication.^[^ ^46^, ^47^ ^]^ Thanks to its high photothermal conversion efficiency and good stability, the potential applications of PTC were further explored. PTC was dissolved in THF (0.5 M) to create patterns such as a four‐pointed star, musical note, and coconut tree using a 2 cm square hollow template. These patterns were used to demonstrate photothermal imaging under 660 nm (0.7 W cm^−2^). As the irradiation time increased, the patterns became clearer and brighter, reaching the maximum temperature within 5 s (Figure 7d), which is advantageous for rapid reading and information extraction. Subsequently, the hollow number “8” template was used as information storage and delivery, where the “27” information was written using PTC ink (0.5 M in THF), and the other part of the template was filled with HPAO + carbon powder ink (0.5 m in THF). The information can be read under room light and UV light as “88”; however, the “27” information can only be read under an IR camera with 660 nm irradiation in 1 s (Figure 7e), demonstrating time‐dependent properties. This good photothermal stability and rapid temperature response enable encryption in two dimensions: information content and reading time, while also allowing for repeated use. Distinct from previous studies on photothermal materials, the PTC cocrystal demonstrates a breakthrough application in information encryption using photothermal effects.^[^ ^40^ ^]^

## 3 Conclusion

In summary, this study introduces a novel approach for constructing donor molecules with planar conformation through intramolecular hydrogen‐bonding interactions, which serve as effective precursors for cocrystals. This approach not only expands the available raw materials for cocrystals but also generates assemblies with emergent properties superior to those of the monomer. Two hydrogen‐bonded interlocked planar conformation molecules, HNAO and HPAO, were successfully assembled with TCB to form two CT cocrystals, NTC and PTC. The NTC cocrystal not only inherits the ESIPT and TPA characteristics from HNAO but also achieves NIR‐I emission facilitated by intermolecular CT interactions in cocrystal form. Its organic microwires exhibit high crystallinity and an ultralow optical loss coefficient of 0.021 dB/µm, which is the lowest reported to date among organic cocrystals, demonstrating significant potential for optical waveguide applications. In contrast, the PTC cocrystal, due to the small energy gap between the HOMO of HPAO and the LUMO of TCB, exhibits a significantly higher DCT (0.0572) in its excited states, which predominantly undergoes non‐radiative transitions, resulting in non‐emissive behavior and efficient photothermal conversion (η = 47.7%). Particularly, the PTC inherits excellent photochemical stability from HPAO, however, PTC presents superior structural stability compared to HPAO and maintains its crystalline structure after at least five cycles of heating and cooling, promotes fast photothermal imaging, and breakthrough time‐dependent information encryption applications. Organic photothermal cocrystal materials, which benefit from their high photothermal efficiency, tunable absorption range, and superior photostability, offer greater competitiveness in photothermal applications compared to traditional polymer‐based materials, liquid crystals, and organic glass. This work not only overcomes the limitations of selecting conjugated (hetero)aromatic compounds to meet the requirements of planarity and rigidity for cocrystal precursors, but also bridges the gap between monomer and their cocrystals.

## Conflict of Interest

The authors declare no conflict of interest.

## Supporting information



Supporting Information

Supporting Information

## Data Availability

The data that support the findings of this study are available in the supplementary material of this article.
