# Supplementary material for: Tunable Photoluminescent and Photothermal Properties of Organic Cocrystals Containing Hydrogen‐Bonded Interlocked Planar Molecules
Source: Adv Sci (Weinh). 2025 Sep 29;12(48):e15054. doi: 10.1002/advs.202515054 (PMC12752575; doi:10.1002/advs.202515054)
Supplement: Supplementary file 1 — Supporting Information [file ADVS-12-e15054-s001.docx]

**Supporting Information**

**Tunable Photoluminescent and Photothermal Properties of Organic Cocrystals Containing Hydrogen-Bonded Interlocked Planar Molecules**

Xinmeng Chen,^1,#^ Ling Zhu,^2,#^ Shan He,^1,#^ Bin Liu,^3^ Qiang Lv,^4^ Xuedong Wang,^4^ Lin Xu^2^, Yu Wang,^5,*^ Ryan T. K. Kwok,^1^ Jacky W. Y. Lam,^1,*^ Lianrui Hu,^2,*^ Wenping Hu^5^ and Ben Zhong Tang^1,6,*^

^1^Department of Chemistry, and the Hong Kong Branch of Chinese National Engineering Research Center for Tissue Restoration and Reconstruction, The Hong Kong University of Science and Technology, Clear Water Bay, Kowloon, Hong Kong, 999077, China

^2^Shanghai Key Laboratory of Green Chemistry and Chemical Processes, Shanghai Frontiers Science Center of Molecule Intelligent Syntheses, School of Chemistry and Molecular Engineering, East China Normal University, Shanghai 200062, China

^3^School of Chemistry, Sun Yat-Sen University, Guangzhou 510006, China

^4^Institute of Functional Nano & Soft Material (FUNSOM), Jiangsu Key Laboratory for Carbon-Based Functional Materials & Devices, Soochow University, Suzhou, Jiangsu 215123, China

^5^Key Laboratory of Organic Integrated Circuit, Ministry of Education & Tianjin Key Laboratory of Molecular Optoelectronic Sciences, Department of Chemistry, School of Science, Tianjin University, Tianjin 300072, China E-mail: yuwangchem@tju.edu.cn

^6^School of Science and Engineering, Shenzhen Institute of Aggregate Science and Technology, The Chinese University of Hong Kong, Shenzhen (CUHK-Shenzhen), Guangdong 518172, China.

^#^These authors contributed equally.

**Table of contents**

[1. Experiment procedure S3](#_Toc208951142)

[1.1 Materials and Chemicals S3](#_Toc208951143)

[1.2 Synthesis of Starting Materials S3](#_Toc208951144)

[1.3 crystal growth condition S10](#_Toc208951145)

[1.4 Theoretical Calculation S10](#_Toc208951146)

[1.5 Details of the transient absorption experiments S11](#_Toc208951147)

[1.6 Fabrication of NTC microwire S11](#_Toc208951148)

[1.7 Calculation of the photothermal conversion efficiency S11](#_Toc208951149)

[1.8 Kinetic model S12](#_Toc208951150)

[2. Figures and Tables S13](#_Toc208951151)

[Table S1 S13](#_Toc208951152)

[Figure S1 S14](#_Toc208951153)

[Figure S2 S14](#_Toc208951154)

[Figure S3 S15](#_Toc208951155)

[Figure S4 S15](#_Toc208951156)

[Figure S5 S15](#_Toc208951157)

[Figure S6 S16](#_Toc208951158)

[Figure S8 S17](#_Toc208951159)

[Table S2 S17](#_Toc208951160)

[Table S3 S18](#_Toc208951161)

[Table S4 S18](#_Toc208951162)

[Figure S9 S18](#_Toc208951163)

[Figure S10 S18](#_Toc208951164)

[Figure S11 S19](#_Toc208951165)

[Figure S12 S19](#_Toc208951166)

[Figure S13 S20](#_Toc208951167)

[Figure S14 S20](#_Toc208951168)

[Figure S15 S21](#_Toc208951169)

[Figure S16 S21](#_Toc208951170)

[Figure S17 S22](#_Toc208951171)

[Figure S18 S23](#_Toc208951172)

[Figure S19 S24](#_Toc208951173)

[Figure S20 S24](#_Toc208951174)

[Figure S21 S25](#_Toc208951175)

[Figure S23 S26](#_Toc208951176)

[Figure S25 S27](#_Toc208951177)

[Figure S26 S28](#_Toc208951178)

[Figure S27 S28](#_Toc208951179)

[Figure S28. S28](#_Toc208951180)

[Figure S28. S29](#_Toc208951181)

[Figure S29 S29](#_Toc208951182)

[Figure S30 S29](#_Toc208951183)

[3. Reference S30](#_Toc208951184)

# 1. Experiment procedure

## 1.1 Materials and Chemicals

Nuclear magnetic resonance (NMR) spectra of ^1^H and ^13^C were tested on a Bruker AVIII 400 MHz NMR spectrometer equipped with a Dual Probe. Chemical shifts (δ) are given in ppm relative to TMS. The residual solvent signals were used as references and the chemical shifts were converted to the TMS scale (CDCl_3_: δ H = 7.26 ppm, δ C = 77.16 ppm). UV/vis absorption spectra were recorded on Shimadzu UV-Visible Spectrophotometer UV-2600i. The photoluminescence (PL) spectra were recorded on a Horiba Fluorolog-3 spectrofluorometer. The absolute fluorescence quantum yield was measured by a calibrated integrating sphere (Labsphere). High-resolution mass spectra (HRMS) were estimated on a GCT premier CAB048 mass spectrometer. Absolute photoluminescence quantum yields were measured using a Hamamatsu absolute PL quantum yield spectrometer C11347 Quantaurus_QY. Single-crystal X-ray diffraction (SXRD) data were collected on a SuperNova, Dual, Cu at home/near, Atlas diffractometer operating at T = 100 K, and crystal structures were solved with Olex2. CCDC 2414733, 2414735, 2414737 and 2414739 contain the supplementary crystallographic data for cocrystal HNAO, HPAO, NTC and PTC, respectively. X-ray diffraction (XRD) pattern was collected on an X’per Pro (PANalytical) at 25 °C (scan range: 3-50°). The upconversion emission and TPA-related measurements were performed on a LSM 880 confocal microscope (Carl Zeiss, Göttingen, Germany). DSC measurements were performed on a TA Instruments Q1000. Temperature was measured with an IR thermal camera (FLIR E76 Thermal Imaging Camera). All digital photos of crystals and amorphous compounds were recorded on a Canon EOS 60D camera.

All chemicals were purchased from J&K Chemistry, Sigma-Aldrich, and TCI, and used directly without further purification. All the solvents were purchased from VWR Chemicals Corp. Anhydrous acetonitrile was used for fluorescence property investigation.

## 1.2 Synthesis of Starting Materials

**Synthesis of (Z)-1-(1-hydroxynaphthalen-2-yl)-3-(phenylamino)prop-2-en-1-one (HNAO)**^S1^

**HNAO** were prepared according to the literature procedure^[S1]^. A mixture of acetonaphenone 1 (1.86 g, 10 mmol) and DMF-DMA (7.1 mL, 20 mmol) in Toluene (20 mL) was heated to 110 °C. After stirring for 24 h at 110 °C, the reaction mixture was filtered and dried under a high vacuum to obtain compound **S1**. Then, The **S1** was dissolved in CH_3_CN (20 mL) and treated with aniline (1.1 mL, 12 mmol) and FeCl_3_ (0.32 g, 2 mmol), The flask was then sealed and stirred at room temperature for 24 h, then was diluted with EA (20 mL), washed with H_2_O (10 mL), HCl 1M (10 mL) and with saturated NaHCO_3_ solution (10 mL). The organic phase was dried with Na_2_SO_4_, filtered, and concentrated under reduced pressure. The residue was purified by flash chromatography to afford **HNAO** (1.3 g, 45% yield) as a yellow solid.

yellow solid, 55% yield, ^1^H NMR (400 MHz, CDCl_3_) δ 14.93 (s, 1H), 11.80 (d, *J* = 12.7 Hz, 1H), 8.49 (m 1H), 7.77 (m, 1H), 7.69 (d, *J* = 8.9 Hz, 1H), 7.64 – 7.49 (m, 3H), 7.45 – 7.36 (m, 2H), 7.28 (d, *J* = 0.9 Hz, 1H), 7.20 – 7.11 (m, 3H), 6.11 (d, *J* = 8.0 Hz, 1H); ^13^C NMR (101 MHz, CDCl_3_) δ 193.9, 162.2, 145.3, 140.0, 136.7, 129.8, 129.1, 127.3, 125.8, 125.5, 124.1, 124.0, 123.7, 117.8, 116.6, 113.2, 92.7; HRMS (ESI) calcd. for C_19_H_15_NO_2_Na [M + Na]^+^ 312.0995, found: 312.0999.

^1^H NMR spectrum of **HNAO**:


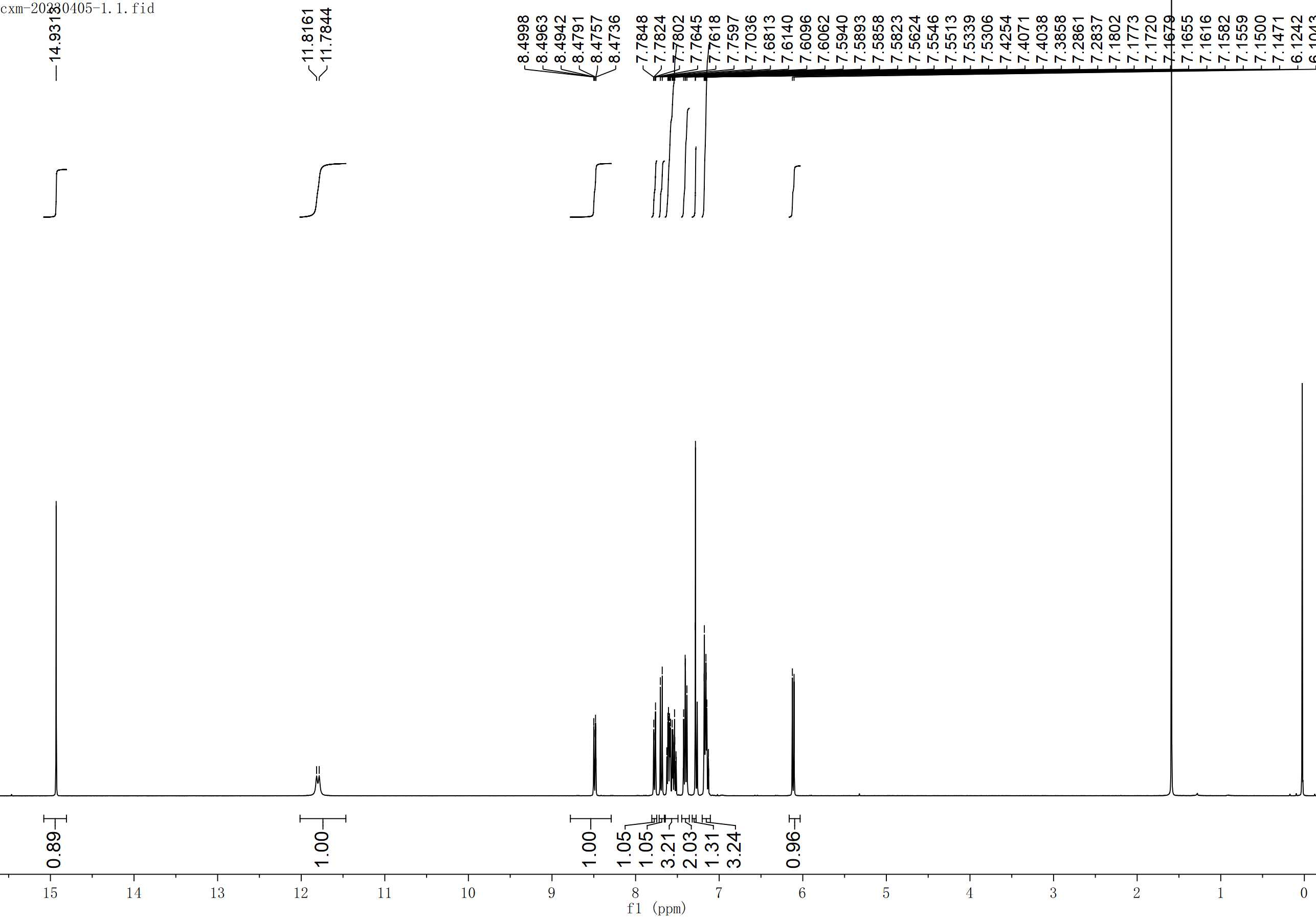


^13^C NMR spectrum of **HNAO**:


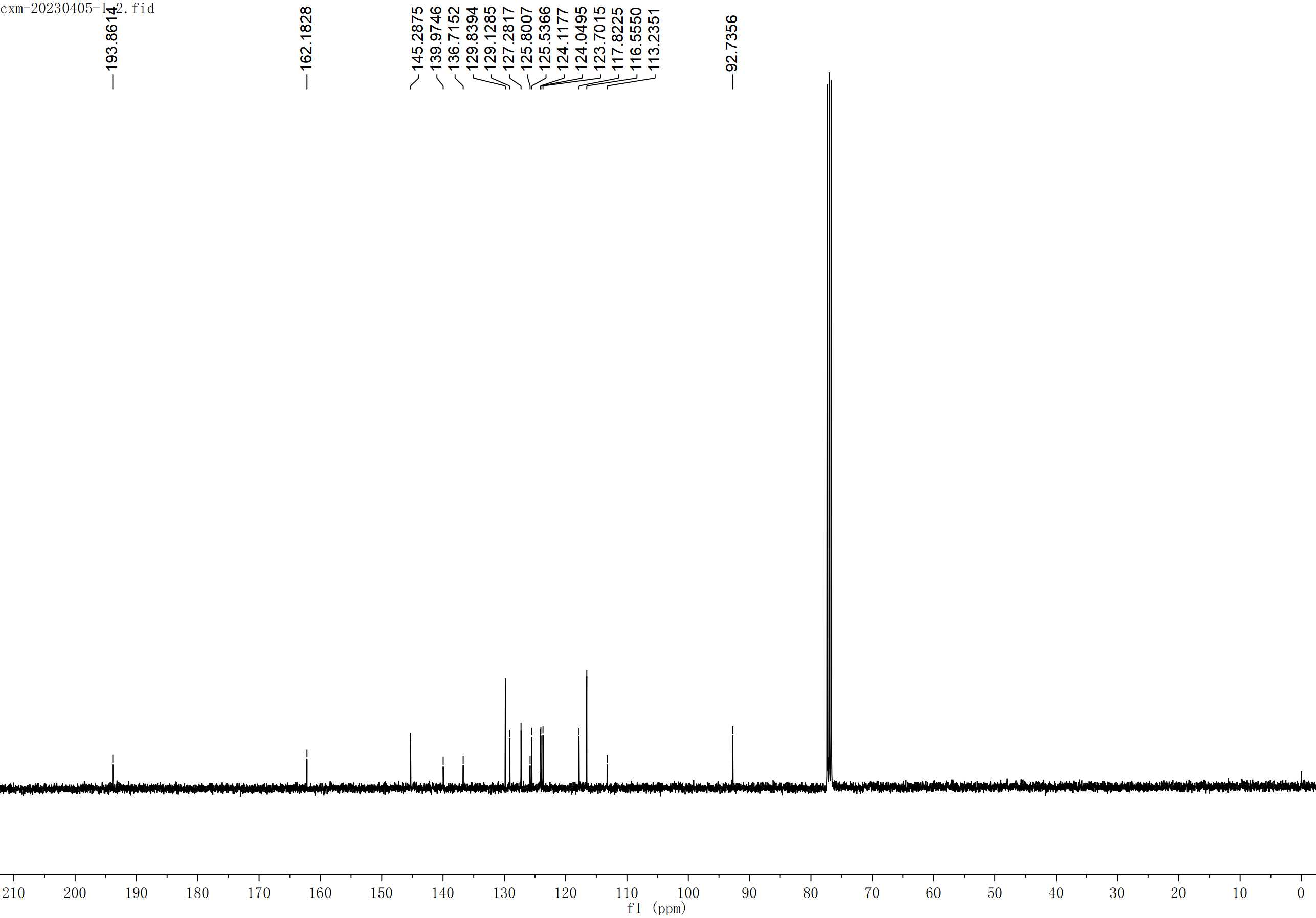


**Synthesis of (Z)-1-(1-hydroxypyren-2-yl)-3-(phenylamino)prop-2-en-1-one (HPAO)**


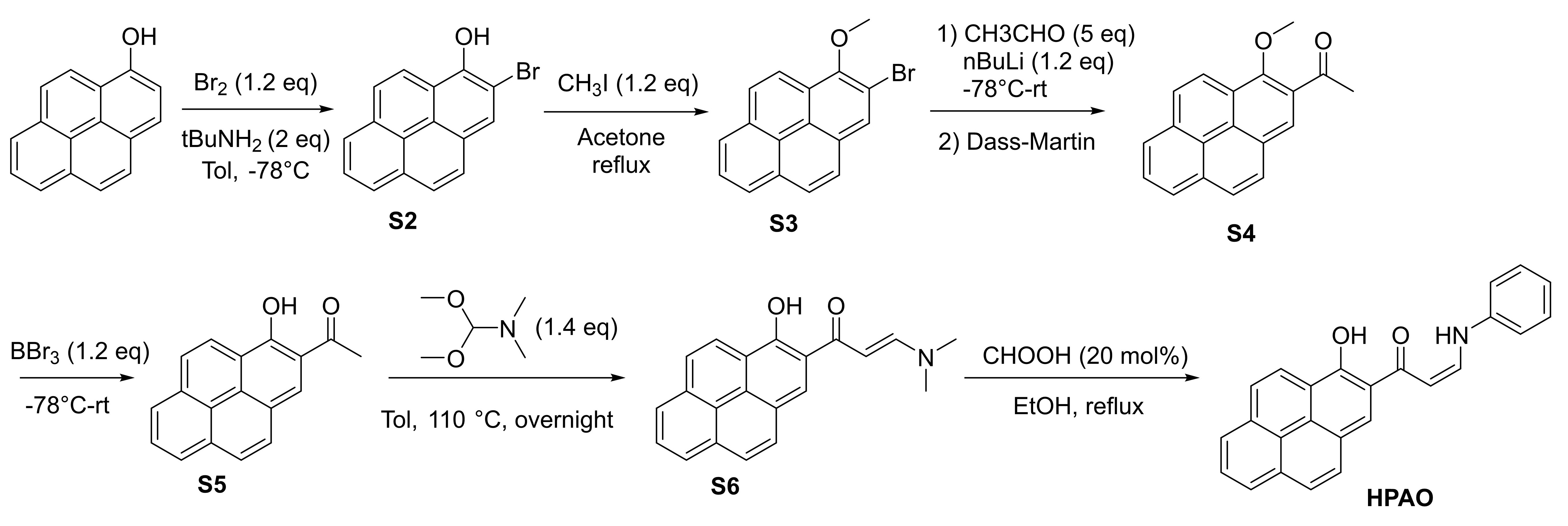


**S2**, **S3** were prepared according to the literature procedure^[S2]^.

**S3** (1.2 g, 4 mmol) in THF (40 mL) was treated at –78 °C dropwise with n-BuLi (2.5 M in n-hexane, 2.0 mL, 5.0 mmol) over 1 min. After stirring for 1 min, anhydrous acetaldehyde in THF solution (5.0 M, 4.0 mL, 20 mmol) was added quickly, and then the reaction mixture was warmed to room temperature overnight. The reaction mixture was carefully quenched with H_2_O (40 mL) and then acidified to pH = 1. Afterwards, the resulting solution was extracted with EA (3 × 20 mL), dried with anhydrous Na_2_SO_4_, filtered, and concentrated under reduced pressure. The crude product was subjected to silica gel flash chromatography (PE: EA = 10:1), and the concentration of the appropriate fractions in vacuo afforded the alcohol compound as a pale-yellow solid. Then, the alcohol compound was dissolved in DCM (20 mL) and treated with Dess–Martin periodinane (1.7 g, 4.0 mmol) and stirred for 2 h. Afterward, saturated aq. NaHCO_3_ (10 mL) and aq. Na_2_S_2_O_3_ (1.0 M, 10 mL) was added simultaneously, extracted with EA (3 × 20 mL), dried with anhydrous Na_2_SO_4_, filtered, and concentrated under reduced pressure. The crude product was subjected to silica gel flash chromatography (PE: EA = 10:1), and the concentration of the appropriate fractions in vacuo afforded **S4** (0.48 g, 41% yield) as a pale-yellow solid.

**1-(1-methoxypyren-2-yl)ethan-1-one (S4)**

^^Pale-yellow solid; 77% yield; ^1^H NMR (400 MHz, CDCl_3_) δ 8.49 – 8.36 (m, 2H), 8.28 – 8.14 (m, 3H), 8.14 – 7.97 (m, 3H), 4.14 (s, 3H), 2.94 (s, 3H); ^13^C NMR (101 MHz, CDCl_3_) δ 201.2, 153.4, 131.7, 131.7, 130.4, 128.1, 127.9, 127.6, 127.6, 127.2, 127.2, 125.5, 125.4, 125.2, 124.7, 124.5, 121.5, 64.5, 31.0; HRMS (ESI) calcd. for C_19_H_14_O_2_Na [M + Na]^+^ 297.0886, found: 297.0888.

^1^H NMR spectrum of **S4**:

^^
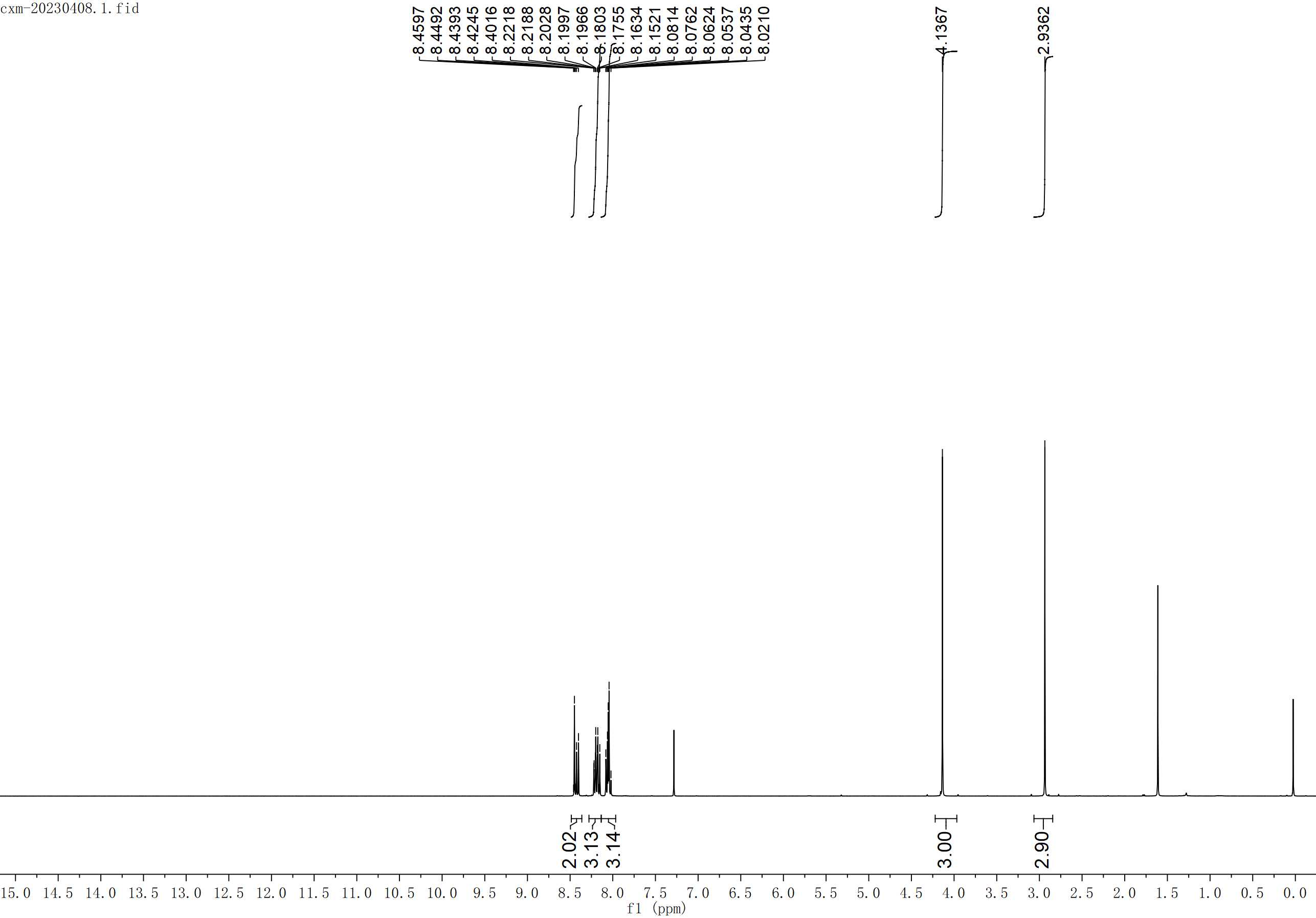


^13^C NMR spectrum of **HNAO**:

^^
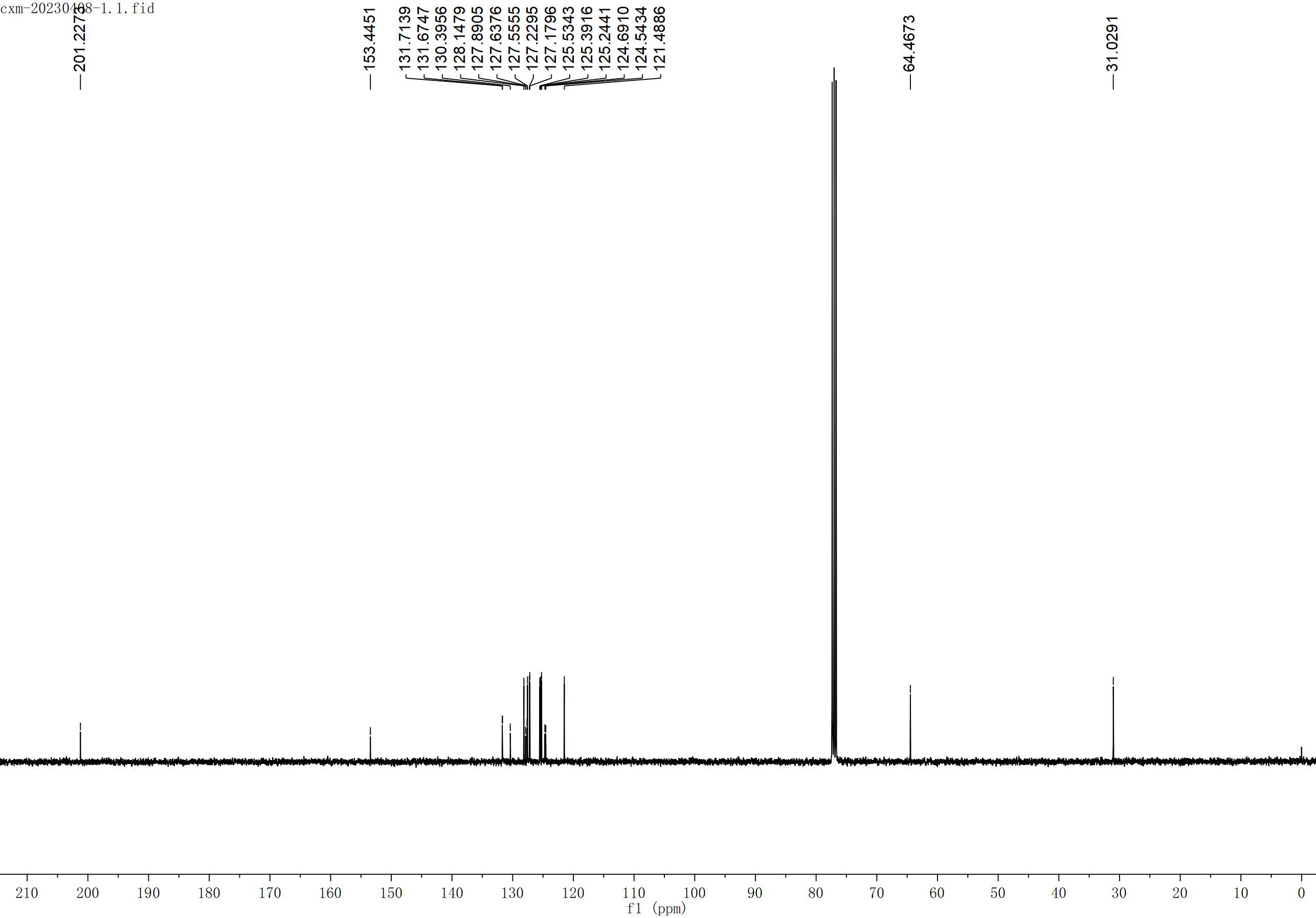


A solution of **S4** (0.42 g, 2.0 mmol) in DCM (20 mL) was treated at –78 °C dropwise with BBr_3_ (1.0 M in DCM, 2.4 mL, 2.4 mmol) over 10 min and stirred overnight at rt. The reaction mixture was quenched with saturated aq. NaHCO_3_ (20 mL). Then it was extracted with EA (3 × 20 mL), dried with anhydrous Na_2_SO_4_, filtered, and concentrated under reduced pressure. The crude product was subjected to silica gel flash chromatography (PE: EA = 10:1), and the concentration of the appropriate fractions in vacuo afforded **S5** (0.49 g, 95% yield) as a pale-yellow solid.

**1-(1-hydroxypyren-2-yl)ethan-1-one (S5)**

Pale-yellow solid; 95% yield; ^1^H NMR (400 MHz, CDCl_3_) δ 13.28 (s, 1H), 8.52 (d, *J* = 9.2 Hz, 1H), 8.34 (s, 1H), 8.10 (m, 1H), 8.08 – 7.97 (m, 3H), 7.81 (d, *J* = 2.2 Hz, 2H), 2.91 (s, 3H); ^13^C NMR (101 MHz, CDCl_3_) δ 205.4, 157.1, 133.0, 132.6, 129.4, 127.8, 127.5, 126.8, 125.9, 125.6, 124.7, 124.6, 124.5, 123.3, 121.8, 120.2, 116.1, 27.2. HRMS (ESI) calcd. for C_18_H_12_O_2_Na [M + Na]^+^ 283.0730, found: 283.0733.

^1^H NMR spectrum of **S5**


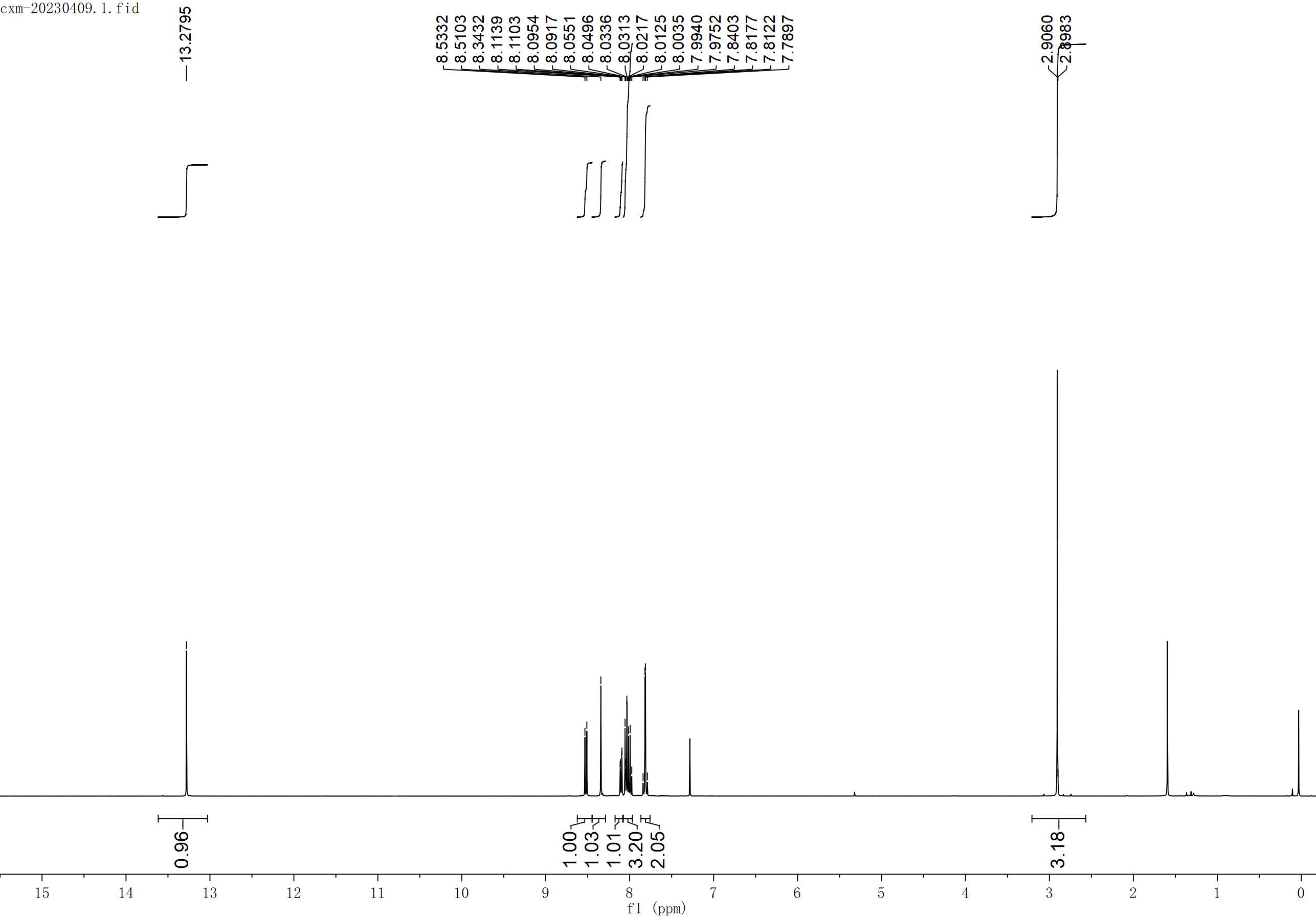


^13^C NMR spectrum of **S5**


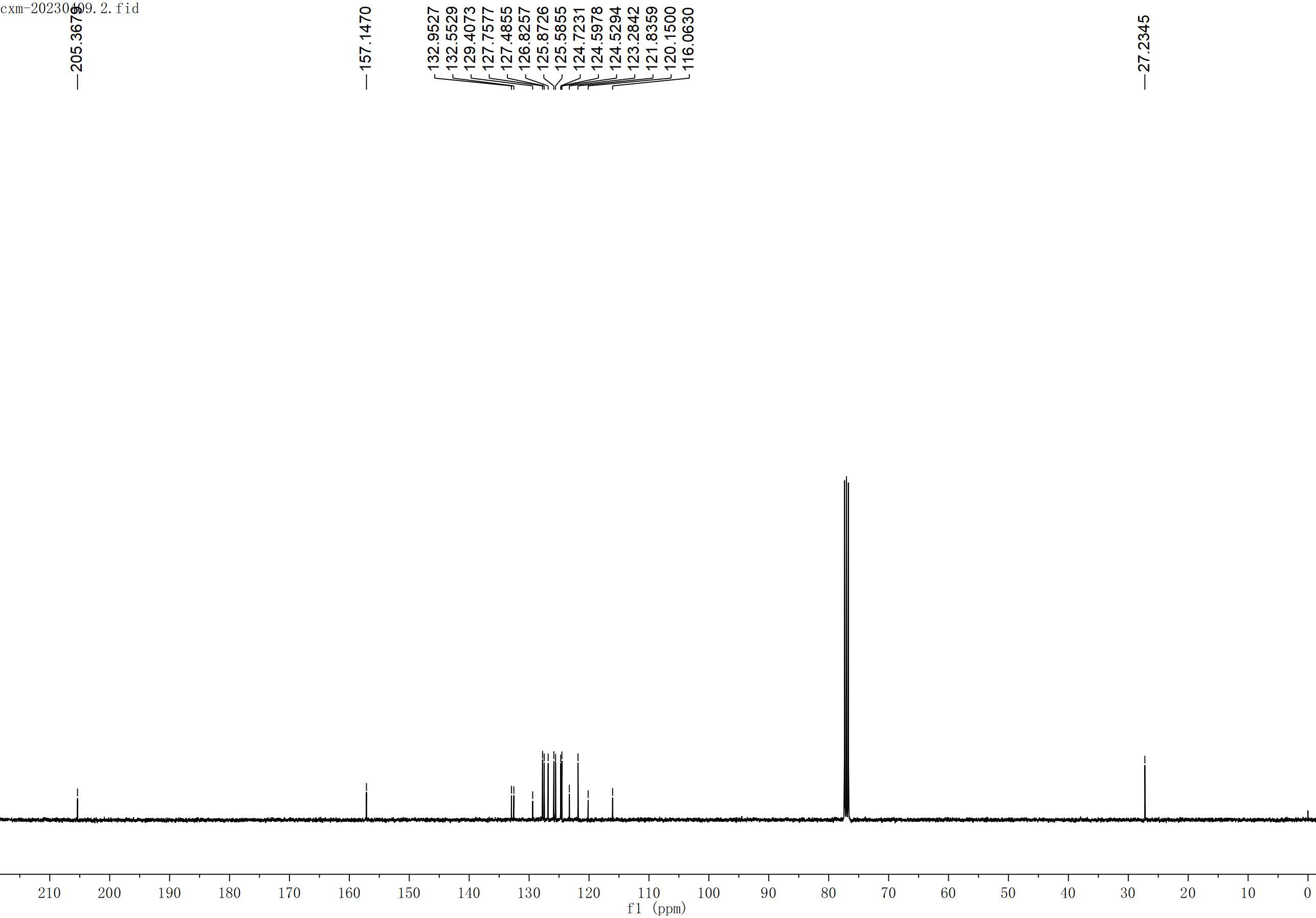


A mixture of **S5** (0.49g, 1.9 mmol) and DMF-DMA (1.4 mL, 4 mmol) in Toluene (20 mL) was heated to 110 °C. After stirring for 24 h at 110 °C, the reaction mixture was filtered and dried under a high vacuum. The crude product was subjected to silica gel flash chromatography (PE: EA = 3:1), and the concentration of the appropriate fractions in vacuo afforded **S6** (0.33 g, 55% yield) as a yellow solid. Next, **S6 (**0.32 mg, 1.0 mmol) and aniline (1.1 mL, 12 mmol) and CH_3_COOH (2 mL) were then refluxed for 24 h, then diluted with EA (20 mL), washed with H_2_O (10 mL), HCl 1M (10 mL), and with saturated NaHCO_3_ solution (10 mL). The organic phase was dried with Na_2_SO_4_, filtered, and concentrated under reduced pressure. The residue was purified by flash chromatography (PE: EA = 5:1) to afford HPAO (36 mg, 10% yield) as an orange-red solid.

**(Z)-1-(1-hydroxypyren-2-yl)-3-(phenylamino)prop-2-en-1-one (HPAO)**

^^Orange red solid; 10% yield; ^1^H NMR (400 MHz, CDCl_3_) δ 14.36 (s, 1H), 11.93 (d, *J* = 12.7 Hz, 1H), 8.60 (d, *J* = 9.1 Hz, 1H), 8.44 (s, 1H), 8.16 – 8.01 (m, 3H), 7.98 (m, 1H), 7.91 (d, *J* = 9.0 Hz, 1H), 7.83 (d, *J* = 9.0 Hz, 1H), 7.68 (m, 1H), 7.48 – 7.39 (m, 2H), 7.24 – 7.13 (m, 3H), 6.41 (d, *J* = 8.0 Hz, 1H); ^13^C NMR (101 MHz, CDCl_3_) δ 194.5, 157.7, 145.9, 139.8, 132.8, 132.5, 129.9, 128.7, 128.7, 127.8, 127.3, 126.4, 125.1, 124.8, 124.5, 124.4, 124.2, 124.0, 123.1, 122.0, 120.4, 116.7, 93.0. HRMS (ESI) calcd. for C_25_H_17_NO_2_Na [M + Na]^+^ 386.1151, found: 386.1150.

^1^H NMR spectrum of **HPAO**


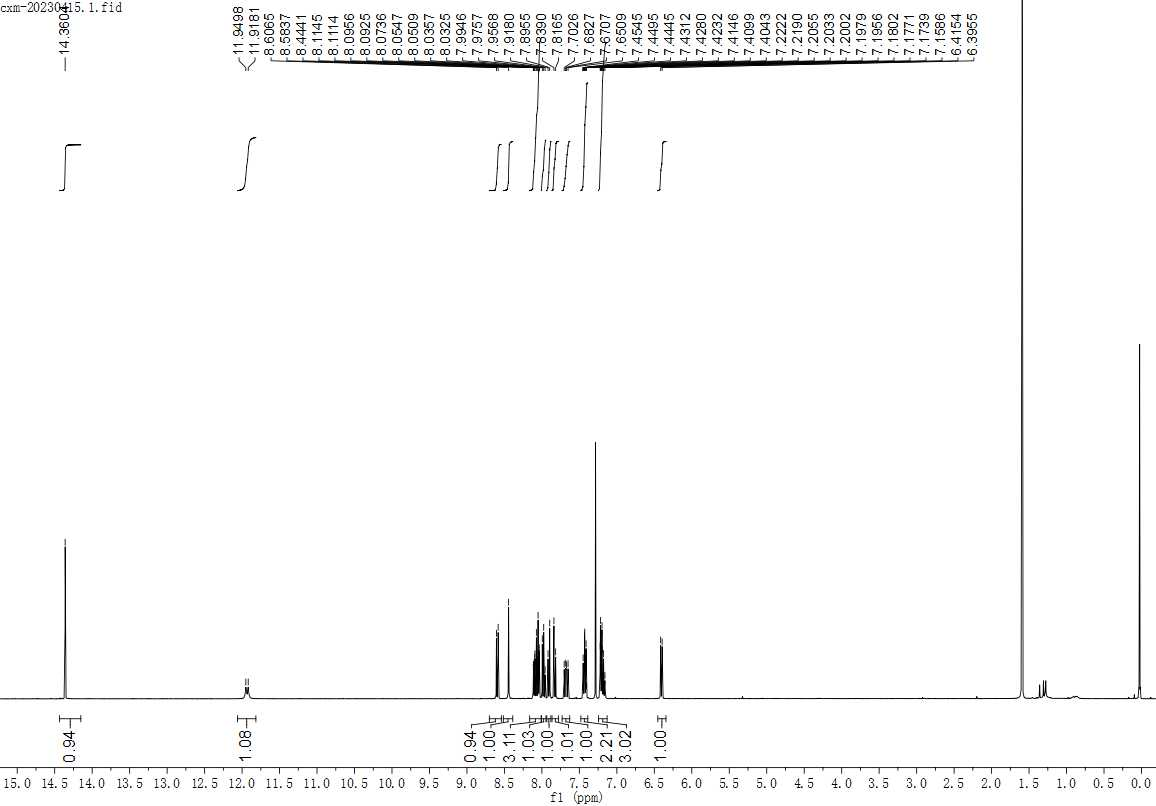


^13^C NMR spectrum of **HPAO**

^^
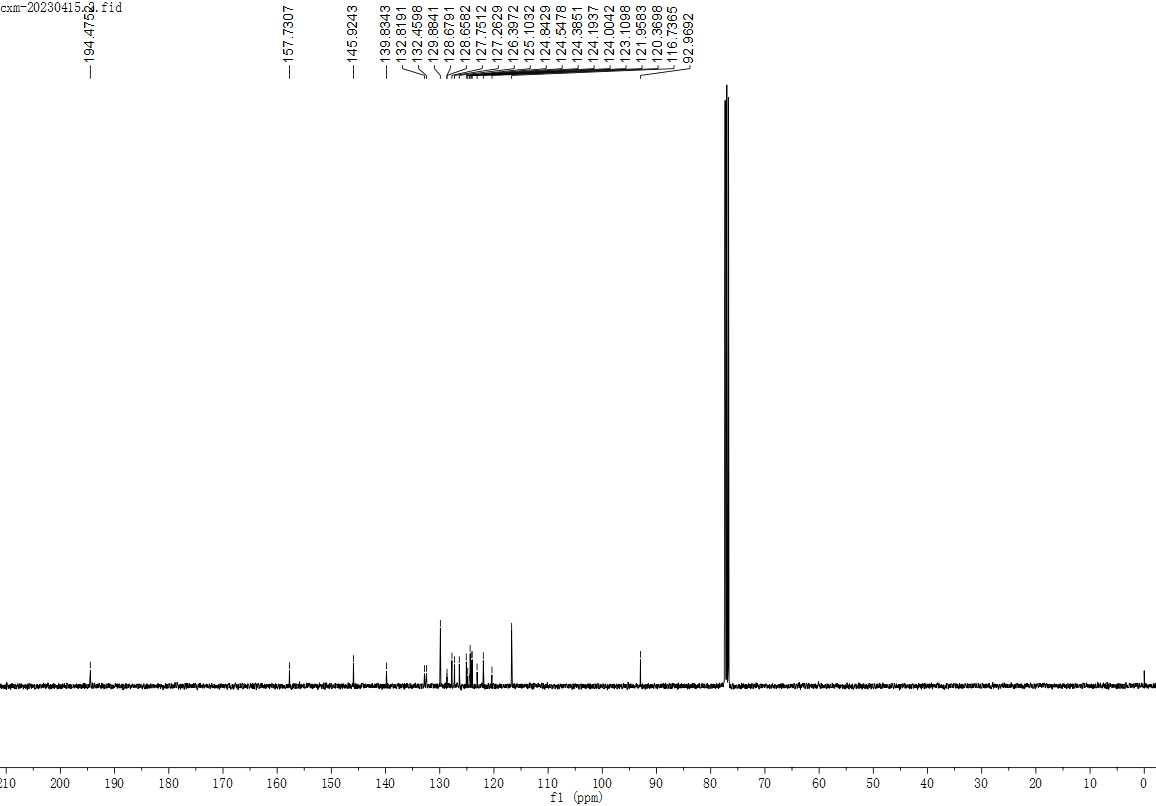


## 1.3 Crystal Growth Conditions

HNAO was prepared by slow solvent evaporation in THF/hexane.

HPAO was prepared by slow solvent evaporation in DCM/hexane.

For cocrystal NTC: HNAO and TCB mixed with the ratio of 1:1 by mol in THF and placed in a hexane atmosphere, slow vapor diffusion of hexane into the THF solution.

PTC was prepared the same way.

## 1.4 Theoretical Calculation

All computations were conducted using Gaussian 16^S3^, employing density functional theory (DFT). The compounds underwent full optimization with the CAM-B3LYP^S4^ functional and a 6-31G(d,p) basis set. To account for long-range inter/intramolecular interactions, Grimme’s DFT-D3 correction was applied to consider London-dispersion effects^S5,S6^. Analytical frequency calculations were performed at the same level of theory to determine whether the calculated structure was intermediate (no virtual frequency) or the transition state (only one virtual frequency). Time-dependent density functional theory (TD-DFT)^S7^ was employed at that level to optimize the excited state geometries and energy levels. Crystal phase geometries were calculated utilizing a combined quantum mechanics and molecular mechanics (QM/MM) model based on crystal packing structures. The central molecule was treated as the QM part at (TD) CAM-B3LYP/6-31G(d,p) level, while the surrounding molecules acted as the MM part with the universal force field (UFF). Free energies were calculated at 298.15 K, 1 atm (unit: kcal/mol). Frontier molecular orbitals (FMO) were visualized using the IQmol molecular viewer package. Hirshfeld surface analysis through Crystal Explorer was employed to analyze intermolecular interactions in crystals. Additionally, UV-Visible absorption, emission spectra, Natural transition orbitals (NTOs) and Noncovalent interactions (NCI) analyses based on optimized structures were computed using Multiwfn 3.8^S8^ and displayed with Visual Molecular Dynamics (VMD)^S9^.

## 1.5 Details of the transient absorption experiments

The femtosecond pump-probe TA measurements are briefly described here. A regenerative amplified Ti:sapphire laser system (Coherent; 800 nm, 70 fs, 6 mJ/pulse, and 1 kHz repetition rate) serves as the laser source. The 800 nm laser pulse is then split into two parts using a beam splitter. The higher-power portion is used to pump an optical parametric amplifier (OPA) to generate a wavelength-tunable excitation laser beam, while the lower-power portion excites a crystal to produce a white light continuum (WLC) as the probe beam. The delay time between the excitation and probe pulses is controlled by a motorized delay stage.

The sample preparation method for TA measurements involves directly placing the powder crystal on a quartz substrate, followed by grinding to form a solid thin film, which is then used for TA measurements.

## 1.6 Fabrication of NTC microwire

The NTC microwires were prepared by microspacing in-air sublimation method. Typically, 0.05 mmol (14.5 mg) HNAO and 0.05 mmol (8.9 mg) TCNB were dissolved in 2 mL DCM. And then the 0.1 ml above solution was injected into 0.2 ml of ethanol, and the mixed solution was directly dropped onto the silicon wafer, and the NTC complex was observed after the solvent evaporated completely. Next, the silicon wafer containing the NTC complex was directly put on a heated stage, and another silicon wafer was placed directly above the substrate's silicon wafer, separated by only 260 μm using small glass spacers, for collecting the NTC under heating at 150°C for 5 minutes. The NTC cocrystals exhibited a 1D morphology, with sizes in the micrometer scale and thickness in the nanometer scale, formed on the underside of the upper substrate.

## 1.7 Calculation of the photothermal conversion efficiency

The photothermal conversion efficiency of the cocrystal was determined according to the previous method. Details are as follows:

Based on the total energy balance for this system:

$$\sum_{i} m_{i}C_{p,i}\frac{dT}{dt}=Q_{s}-Q_{loss}$$

Where *m_i_* (0.022 g) and *Cp*,*i* (1.618 J/(g.°C) ) are the mass and heat capacity of system components (cocrystal samples and substrate), respectively. *Q_s_* is the photothermal heat energy input by irradiating NIR laser to cocrystal samples, and *Q_loss_* is thermal energy lost to the surroundings. When the temperature is maximum, the system is in balance

$$Q_{s}=Q_{loss}=hS\Delta T_{max}$$

Where *h* is heating transfer coefficient, *S* is the surface area of the container, *ΔT_max_* is the maximum temperature change.

The photothermal conversion efficiency $\eta$ is calculated from the following equation:

$$\eta=\frac{hS\Delta T_{max}}{I(1-{10}^{-A_{808}})}$$

Where *I* is the laser power (0.9 W/cm^2^) and *A*_808_ is the absorbance of the samples at the wavelength of 808 nm (0.251).

In order to get the *hS* , a dimensionless driving force temperature, *θ* is introduced as follows:

$$\theta=\frac{T-T_{surr}}{T_{max}-T_{surr}}$$

Where *T* is the temperature of cocrystal, *T_max_* is the maximum system temperature (78 °C), and *T_surr_* is the initial temperature (25 °C).

And a sample system time constant *τ_s_*

$$\tau_{s}=\frac{\sum_{i} m_{i}C_{p,i}}{hS}$$

Thus, $\frac{d\theta}{dt}=\frac{1}{\tau_{s}}\frac{Q_{s}}{hS\Delta T_{max}}-\frac{\theta}{\tau_{s}}$

When the laser is off,$Q_{s}=0$, therefore, $\frac{d\theta}{dt}=\frac{\theta}{\tau_{s}}$, and $t= -\tau_{s}\ln\theta$

So *hS* could be calculated from the slope of cooling time vs *In* *θ*. Therefore, *τ_s_* is 9.8 s (Figure S20b). And the photothermal conversion efficiency $\eta$ is 47.7%.

Additionally, in HPAO system, *m_i_* (0.022 g) and *Cp*,*i* (1.201 J/(g.°C) ), *I* (2.5 W/cm^2^), *A*_405_ (0.840), *T_max_*(54 °C), Therefore, *τ_s_* is 0.89 s (Figure S20d), $\eta$ is 40.3%.

## 1.8 Kinetic model

The S_1_ population has three relaxation channels; radiation and non-radiation. The fitted rates are:

$$k_{f}=k_{r}+k_{nr}=\frac{1}{\tau_{avg}}$$

The PLQY and the triplet yield can be depicted as:

$$Ф_{\mathrm{QY}}=\frac{k_{r}}{k_{r}+k_{nr}}$$

Thereby,

$$k_{r}=k_{f}\timesФ_{\mathrm{QY}}$$

According to the PLQY result in HNAO and NTC system (Table S4),

Thus,

$$k_{nr}=k_{f}\times(1-Ф_{\mathrm{QY}})$$

Therefore, the *τ*_avg_ of HNAO is 74.8 ps, and the *k*_nr_ is 10.4 ns^-1^. while the *τ*_avg_ of NTC is 138.2 ps, and the *k*_nr_ is 7.1 ns^-1^.

According to the PLQY result in HPAO and PTC system (Table S4), $Ф_{\mathrm{QY}}=0$

Thus, $k_{r}=0$

The fitted rate of can be calculated from the following equation:

$$k_{f}=k_{nr}=\frac{1}{\tau_{avg}}$$

Therefore, the $\tau_{avg}$ of HPAO is 12.8 ps, and the $k_{nr}$ is 78.1 ns^-1^. while the $\tau_{avg}$ of PTC is 57.4 ps, and the $k_{nr}$ is 17.4 ns^-1^.

# 2. Figures and Tables

Table S1. Crystallographic Data for HNAO and HPAO.

| **compound** | **HNAO** | **HPAO** |
| --- | --- | --- |
| CCDC | 2414733 | 2414735 |
| Empirical formula | C_19_H_15_NO_2_ | C_25_H_17_NO_2_ |
| Formula weight | 289.32 | 363.39 |
| Temperature/K | 173.00(10) | 100.01(10) |
| Crystal system | monoclinic | triclinic |
| Space group | P2_1_/n | P-1 |
| a/Å | 13.9884(3) | 8.0635(2) |
| b/Å | 5.42628(11) | 13.8566(6) |
| c/Å | 18.4158(4) | 15.7679(6) |
| α/° | 90 | 78.415(4) |
| β/° | 93.787(2) | 83.166(3) |
| γ/° | 90 | 89.689(3) |
| Volume/Å^3^ | 1394.80(5) | 1713.34(12) |
| Z | 4 | 4 |
| ρ_calc_g/cm^3^ | 1.378 | 1.409 |
| μ/mm^‑1^ | 0.717 | 0.710 |
| F(000) | 608.0 | 760.0 |
| Goodness-of-fit on F^2^ | 1.020 | 1.029 |
| Final R indexes [I>=2σ (I)] | R_1_ = 0.0387, wR_2_ = 0.1008 | R_1_ = 0.0672, wR_2_ = 0.1894 |
| Final R indexes [all data] | R_1_ = 0.0513, wR_2_ = 0.1101 | R_1_ = 0.0832, wR_2_ = 0.2103 |


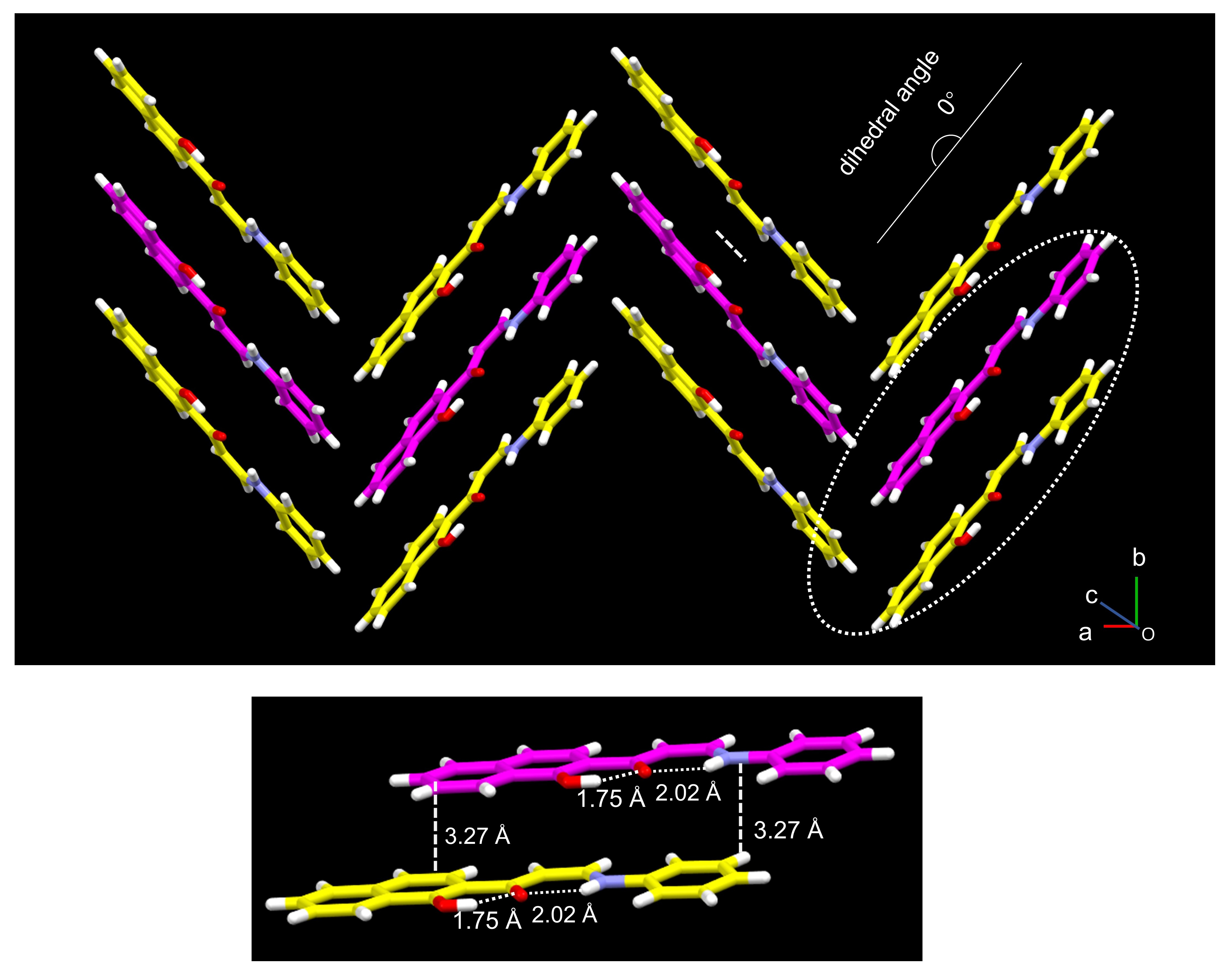


Figure S1. Intermolecular distances and molecular packing structures of HNAO measured by XRD analysis.


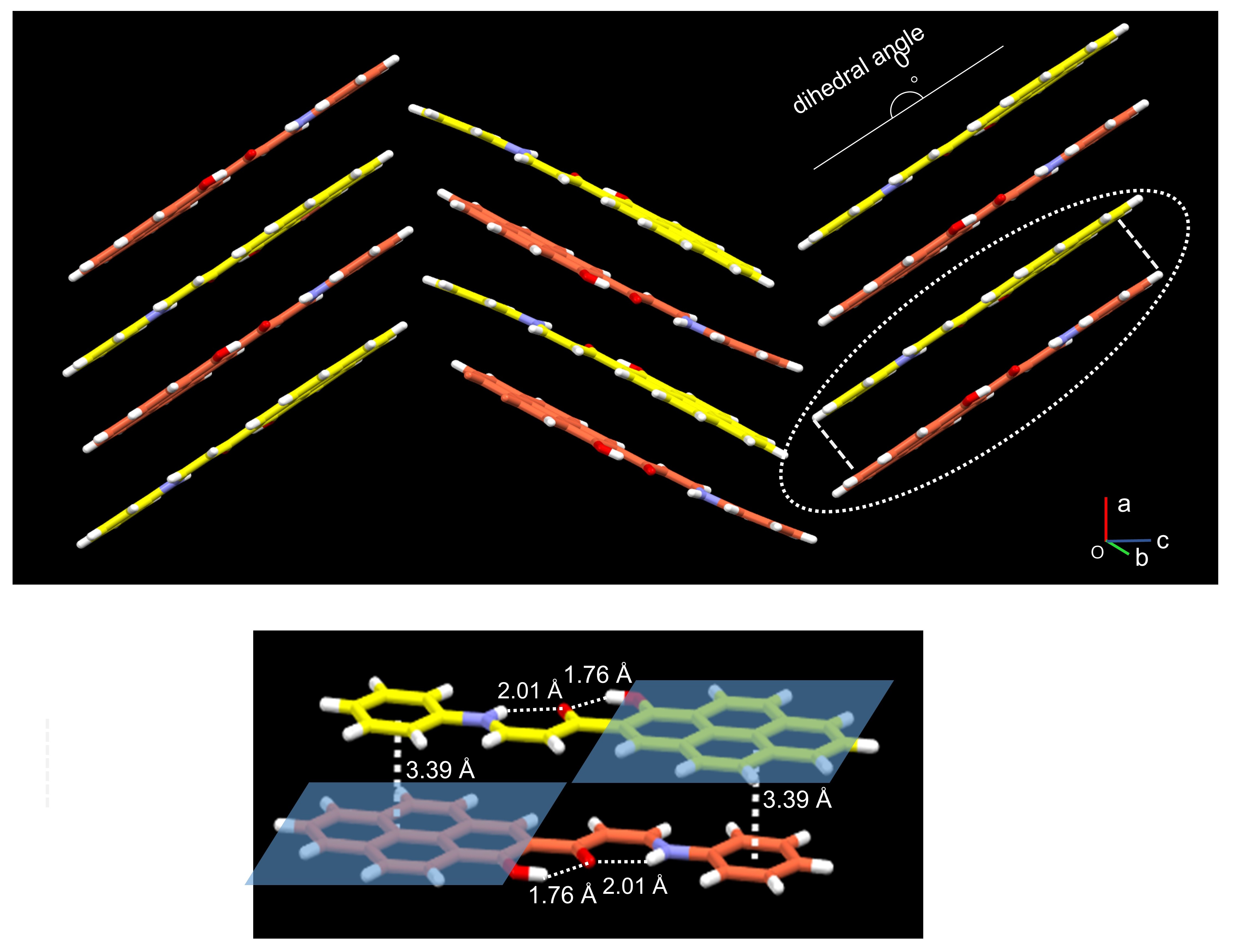


Figure S2. Intermolecular distances and molecular packing structures of HPAO measured by XRD analysis.


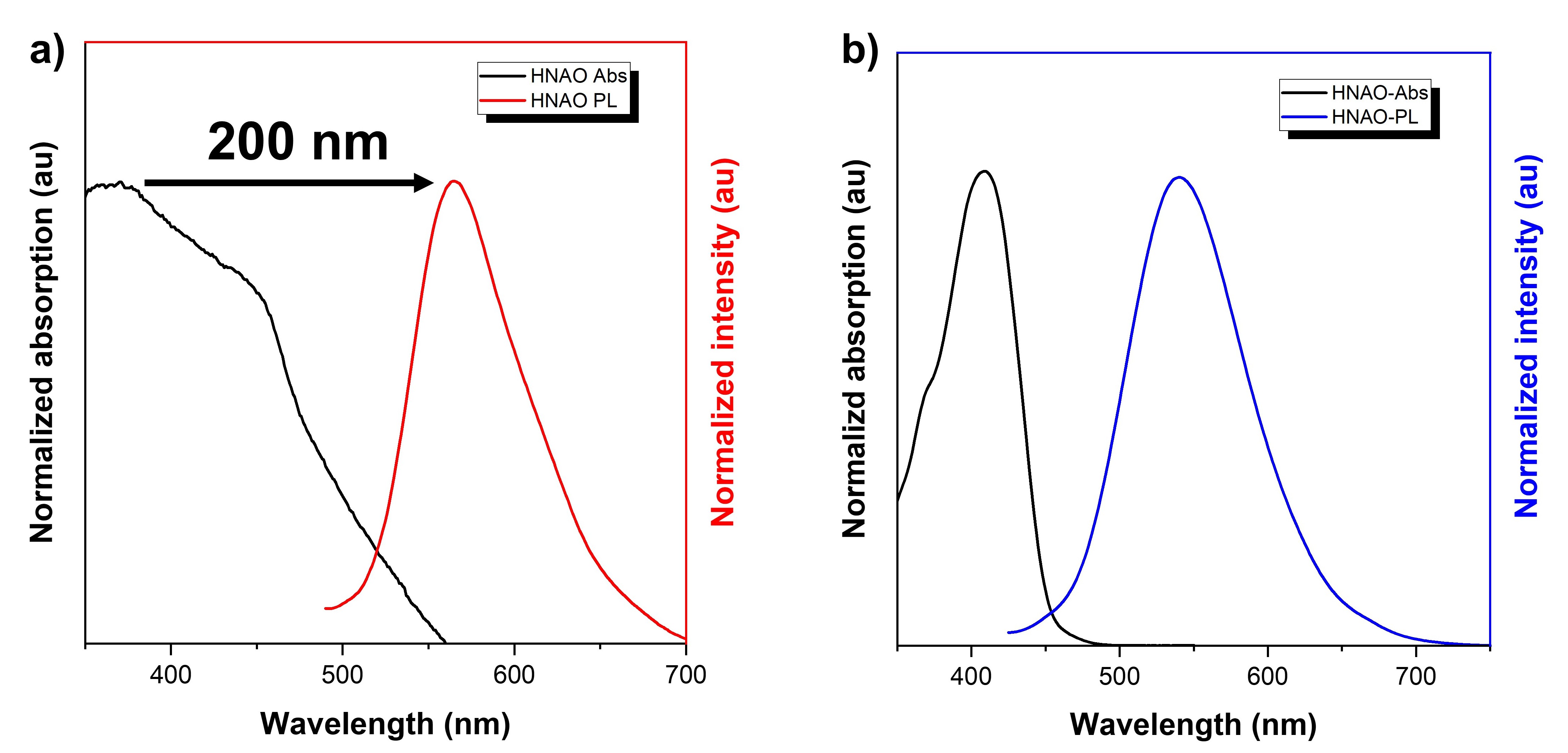


Figure S3. a) Absorption and PL spectra of HNAO in the solid state. *λ*_ex_ = 380 nm. b) Absorption and PL spectra of HNAO in the solution state in THF. *c* = 10^-5^ M, *λ*_ex_ = 380 nm.


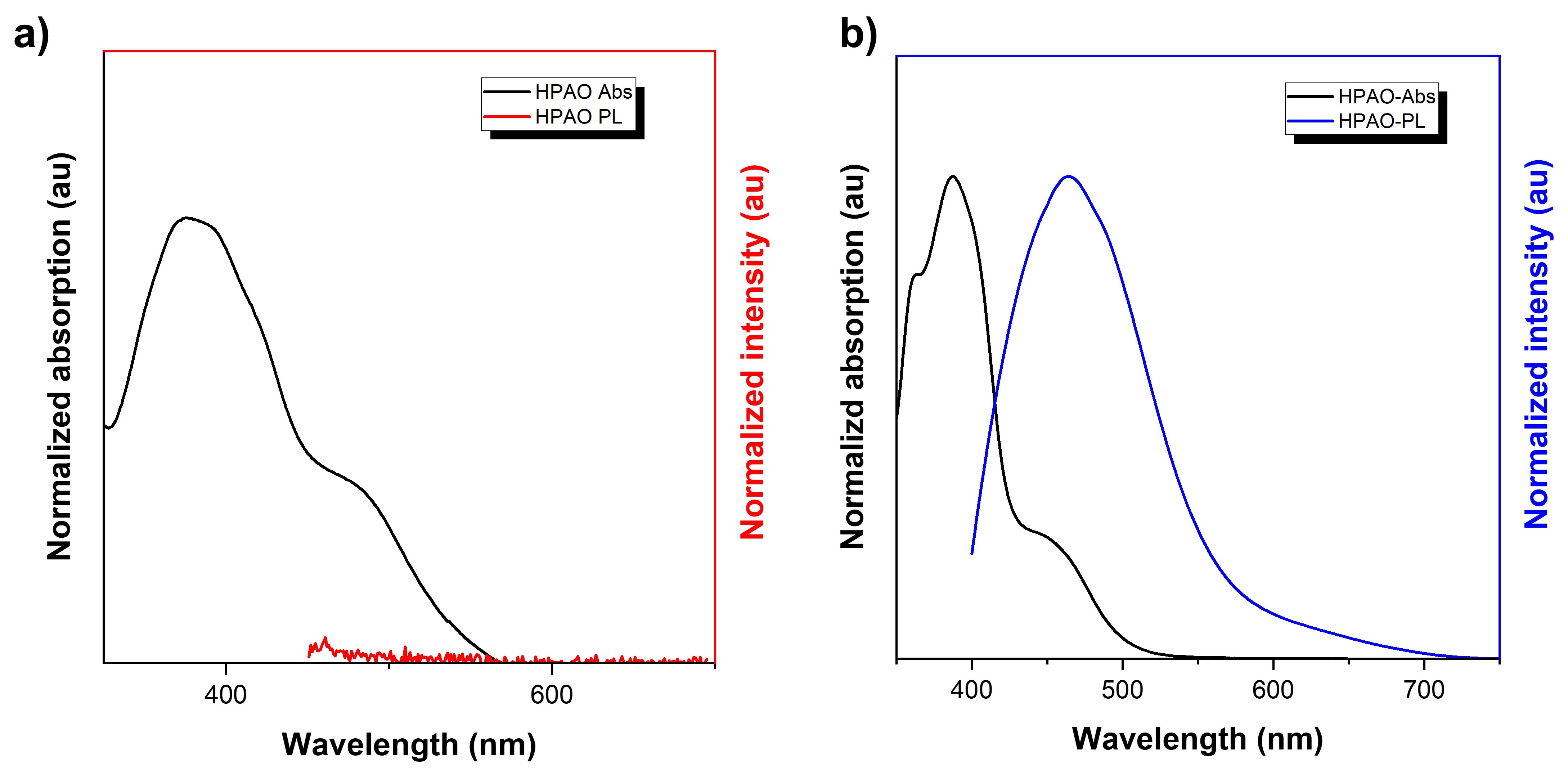


Figure S4. a) Absorption and PL spectra of HPAO in the solid state. *λ*_ex_ = 380 nm. b) Absorption and PL spectra of HPAO in the solution state in THF. *c* = 10^-5^ M, *λ*_ex_ = 380 nm.


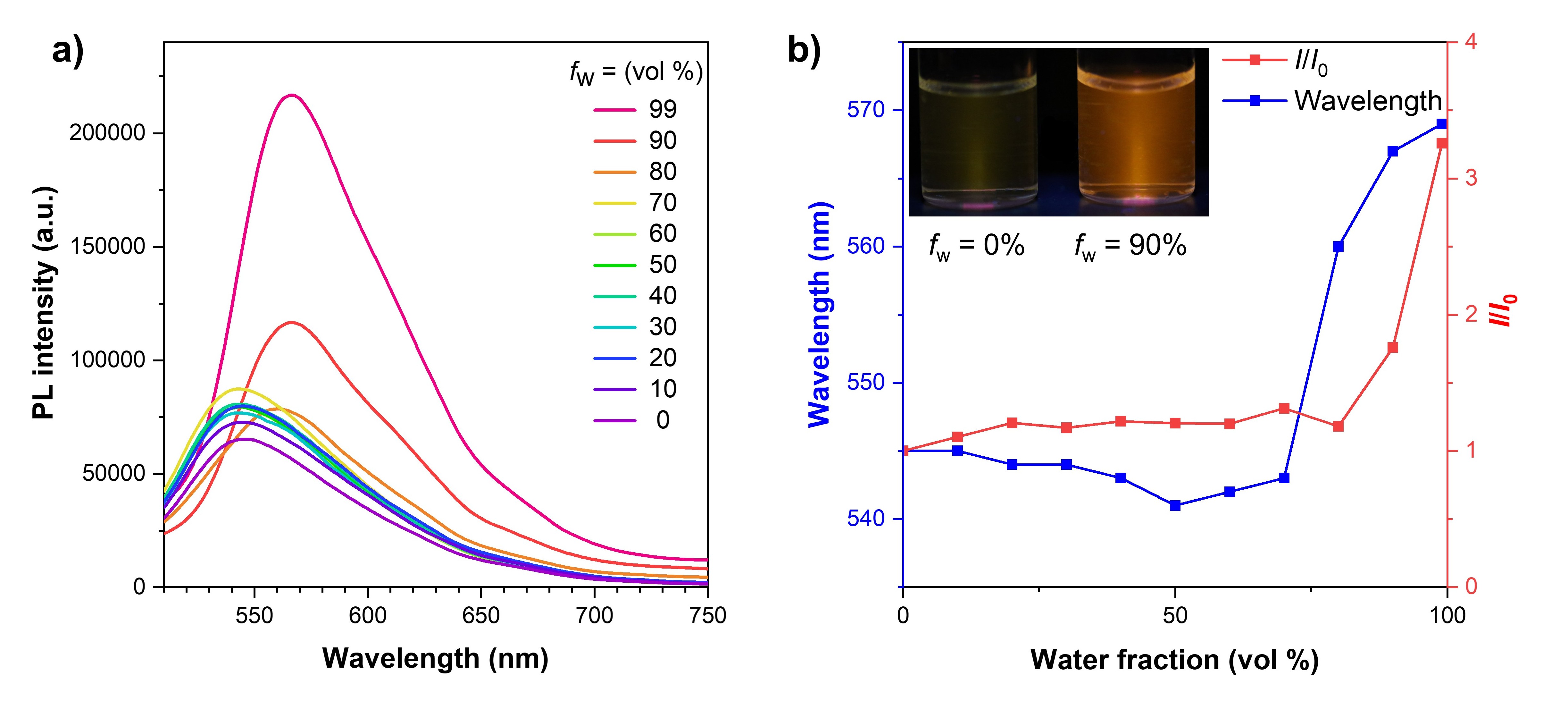


Figure S5. a) PL spectra of HNAO in THF/water mixtures with different water fractions (*f*_w_). *c* = 10^−5^ M, *λ*_ex_ = 380 nm. b) Plots of relative PL intensity (*I*/*I*_0_) versus *f*_w_ at different emission wavelengths. *I*_0_ = PL intensity at *f*_w_ = 0%.


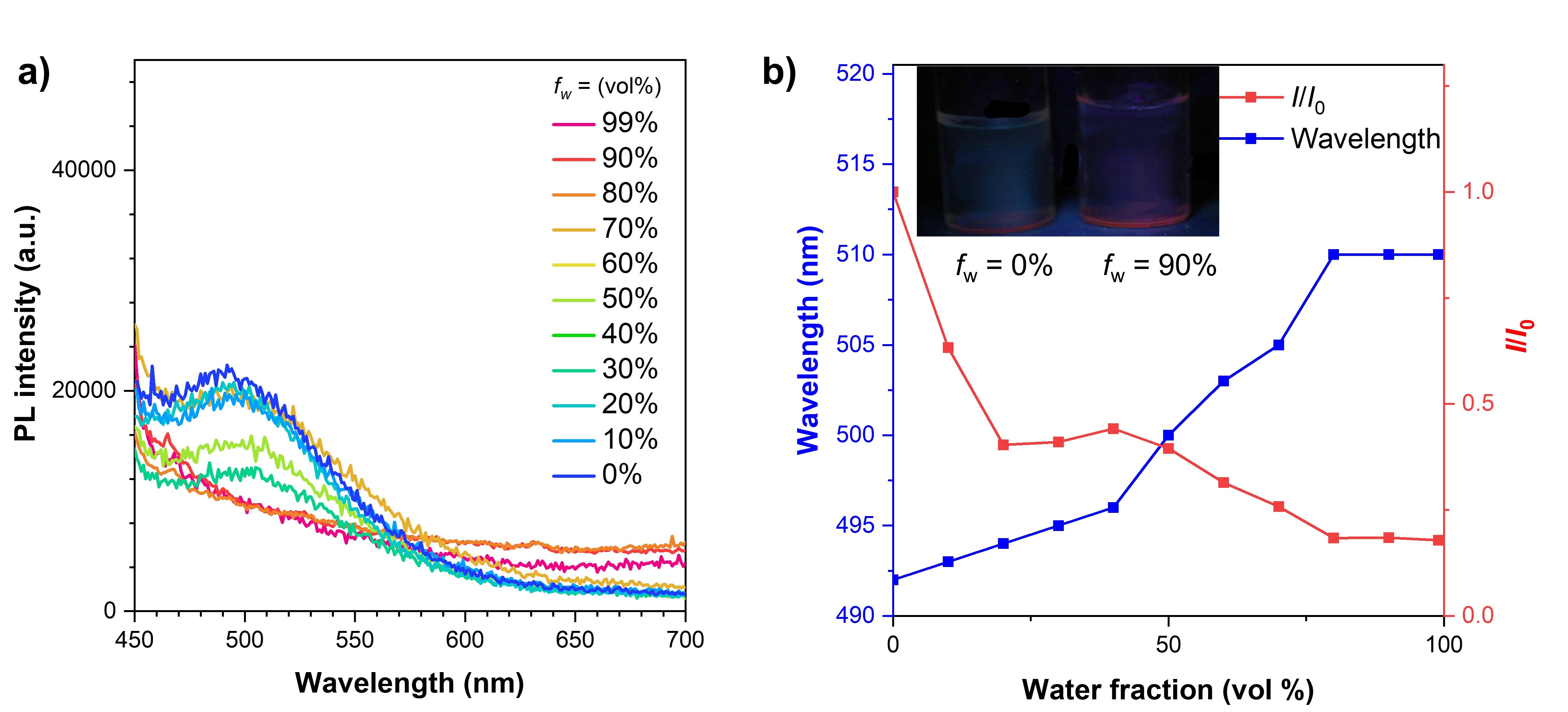


Figure S6. a) PL spectra of HPAO in THF/water mixtures with different water fractions (*f*_w_). *c* = 10^−5^ M, *λ*_ex_ = 380 nm. b) Plots of relative PL intensity (*I*/*I*_0_) versus *f*_w_ at different emission wavelengths. *I*_0_ = PL intensity at *f*_w_ = 0%.


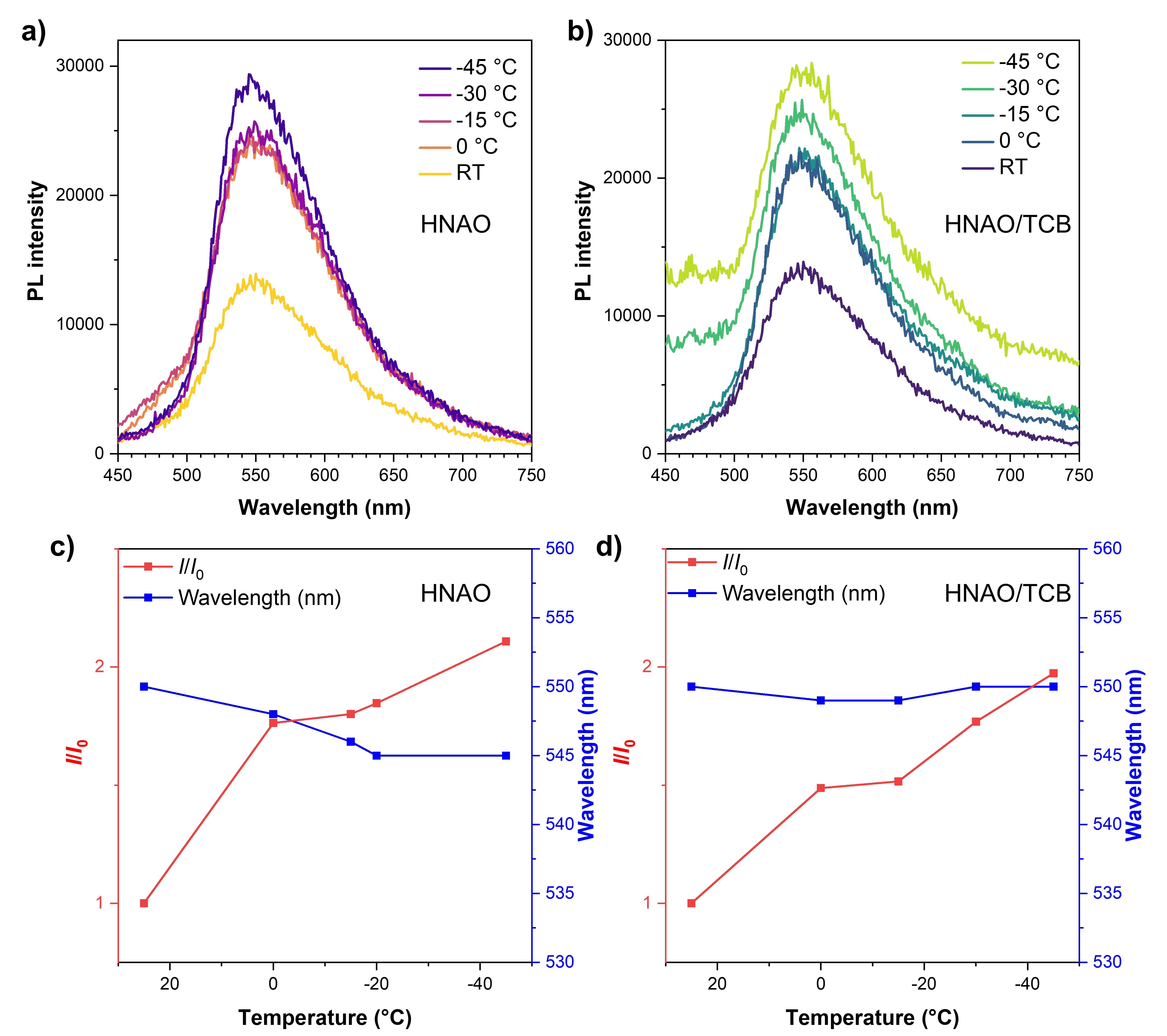


Figure S7. Temperature-dependent PL spectra of a) HNAO and b) HNAO/TCB during cooling from room temperature to -45 °C with excitation wavelengths at 405 nm. Plots of relative PL intensity (*I*/*I*_0_) versus temperature at different emission wavelengths of c) HNAO and d) HNAO/TCB. *I*_0_ = PL intensity at room temperature.


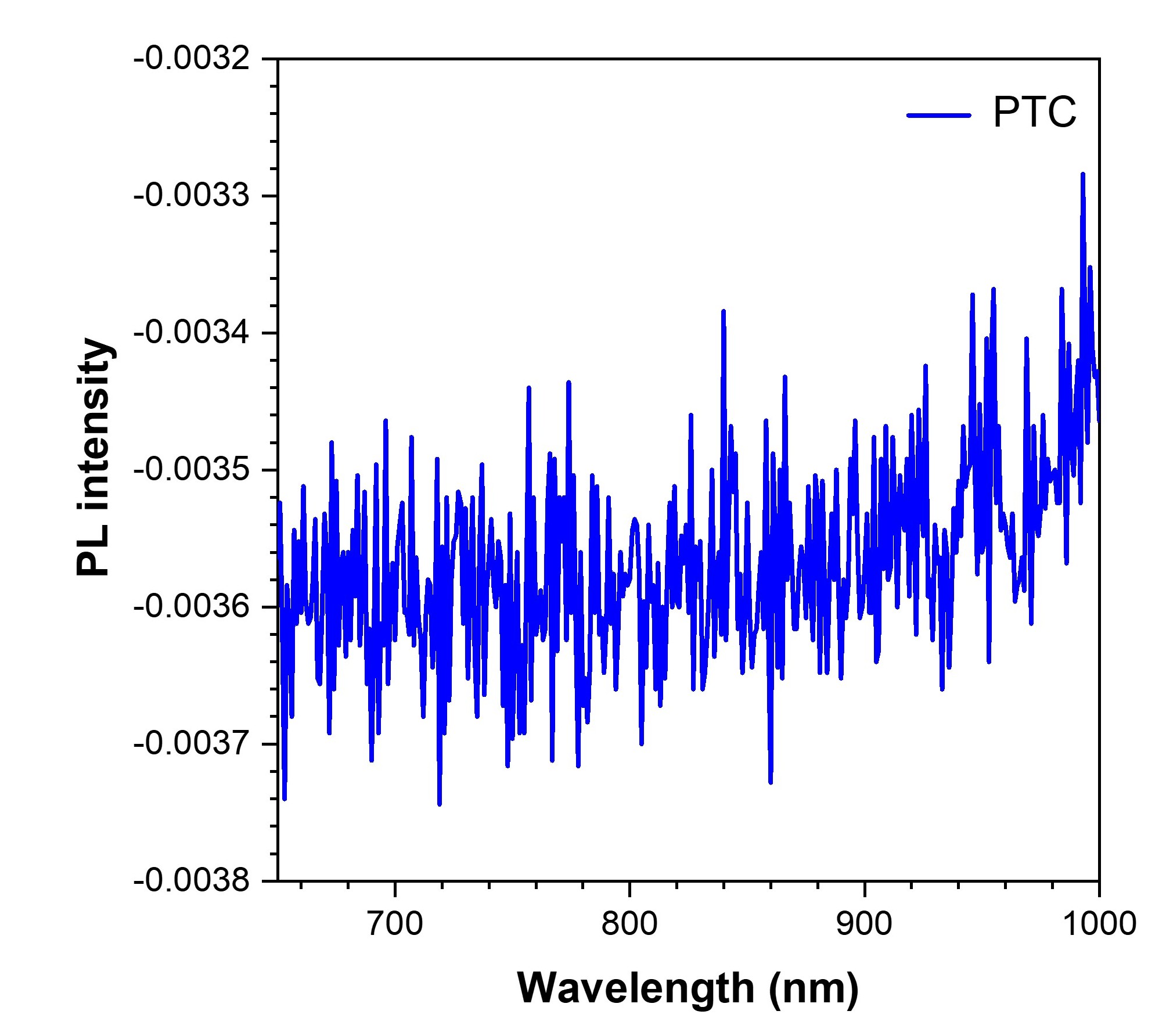


Figure S8 a) PL spectra of PTC in the solid state. *λ*_ex_ = 600 nm.

Table S2. Crystallographic Data for NTC and PTC.

| **compound** | **NTC** | **PTC** |
| --- | --- | --- |
| CCDC | 2414737 | 2414739 |
| Empirical formula | C_29_H_17_N_5_O_2_ | C_35_H_19_N_5_O_2_ |
| Formula weight | 467.47 | 541.55 |
| Temperature/K | 100.01(10) | 100.01(10) |
| Crystal system | triclinic | triclinic |
| Space group | P-1 | P-1 |
| a/Å | 6.7652(5) | 7.9455(5) |
| b/Å | 7.8533(5) | 11.8990(8) |
| c/Å | 21.4848(12) | 14.9787(7) |
| α/° | 87.892(5) | 107.731(5) |
| β/° | 82.088(5) | 104.054(5) |
| γ/° | 75.741(6) | 98.966(5) |
| Volume/Å^3^ | 1095.77(13) | 1267.36(13) |
| Z | 2 | 2 |
| ρ_calc_g/cm^3^ | 1.417 | 1.419 |
| μ/mm^‑1^ | 0.747 | 0.731 |
| F(000) | 484.0 | 560.0 |
| Goodness-of-fit on F^2^ | 1.035 | 1.022 |
| Final R indexes [I>=2σ (I)] | R_1_ = 0.0495, wR_2_ = 0.1283 | R_1_ = 0.0562, wR_2_ = 0.1547 |
| Final R indexes [all data] | R_1_ = 0.0666, wR_2_ = 0.1424 | R_1_ = 0.0707, wR_2_ = 0.1730 |

Table S3. Decay rate of excited state.

|  | a_1_ | *τ_1_* (ps) | a_2_ | *t_2_* (ps) | a_3_ | *t_3_* (ps) | *t_ave_* (ps)^a^ |
| --- | --- | --- | --- | --- | --- | --- | --- |
| NTC | 0.19 | 2.44 | 0.35 | 47.4 | 0.39 | 285.8 | 138.2 |
| HNAO | 0.26 | 1.02 | 0.51 | 22.3 | 0.22 | 283.5 | 74.8 |
| PTC | 0.25 | 2.7 | 0.57 | 26.3 | 0.09 | 405.9 | 57.4 |
| HPAO | 0.36 | 1.21 | 0.43 | 9.1 | 0.14 | 54.2 | 12.8 |

^a^Equation: *τ*_avg =_ a_1_*τ*_1+_ a_2_*τ*_2+_ a_3_*τ*_3_

Table S4**.** PLQY and *k*_nr_ of NHAO, HPAO, NTC and PTC

|  | HNAO | HPAO | NTC | PTC |
| --- | --- | --- | --- | --- |
| PLQY(%) | 22.1 | 0 | 2.1 | 0 |
| *k*_nr_ | 10.4 | 78.1 | 7.1 | 17.4 |


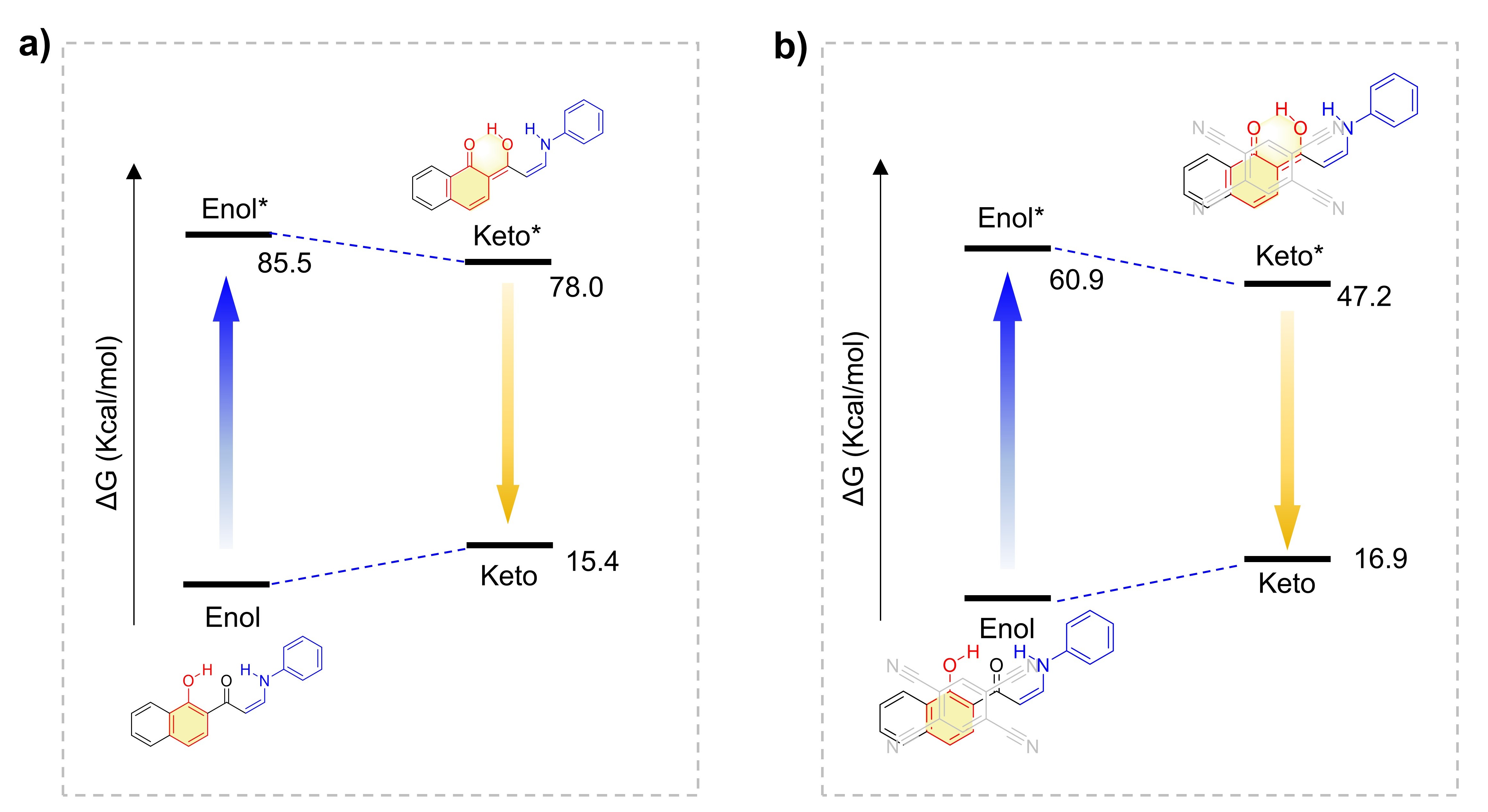


Figure S9. Calculated relative energies (kcal/mol) of the enol and keto forms of a) HNAO and b) HNAO in the cocrystal system.


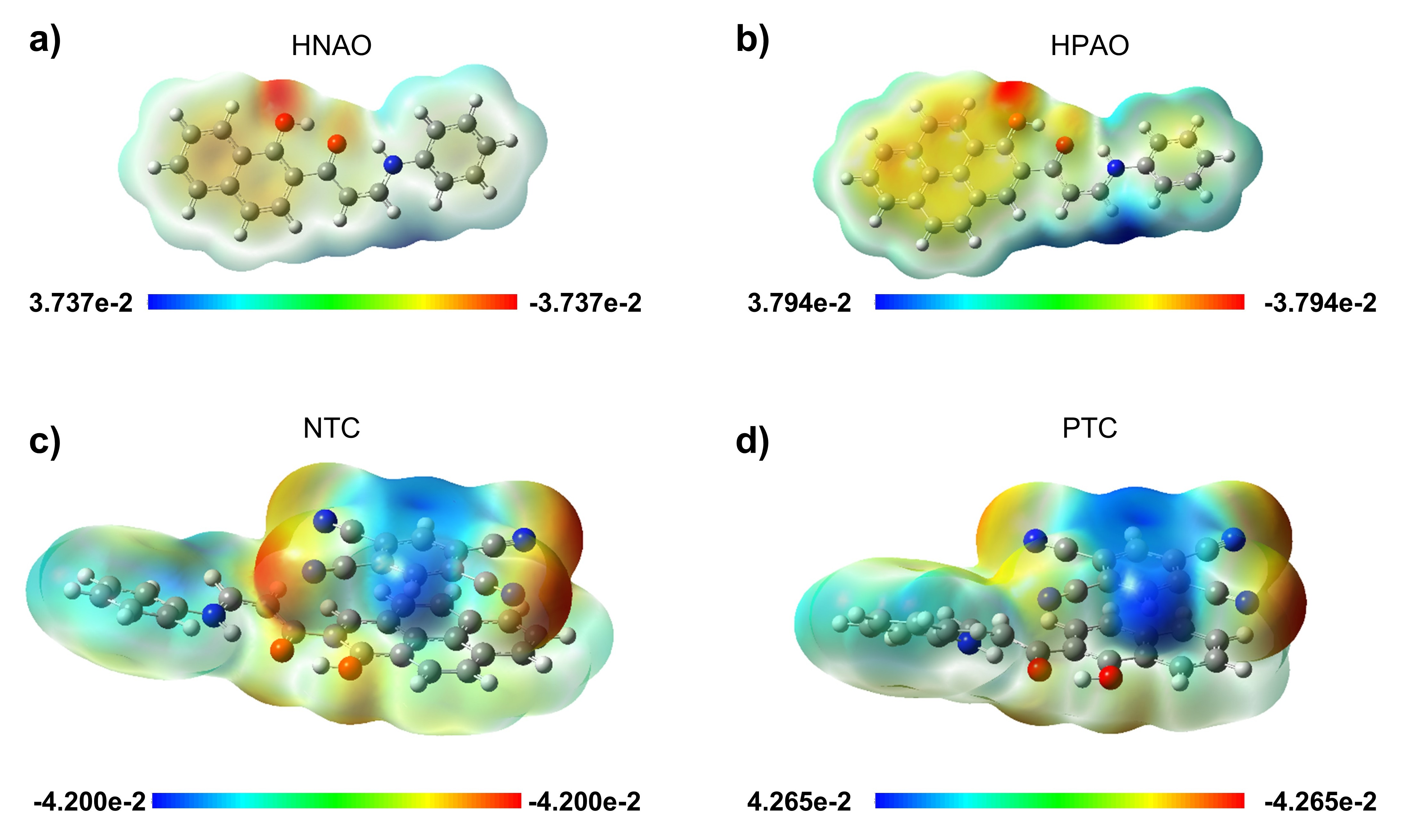


Figure S10. Electronic static potential mapped on the isosurface of electronic density, based on their optimized ground-state geometries. A negative electrostatic potential (red) represents a high electronic density, whereas a positive one (blue) corresponds to a low electronic density.


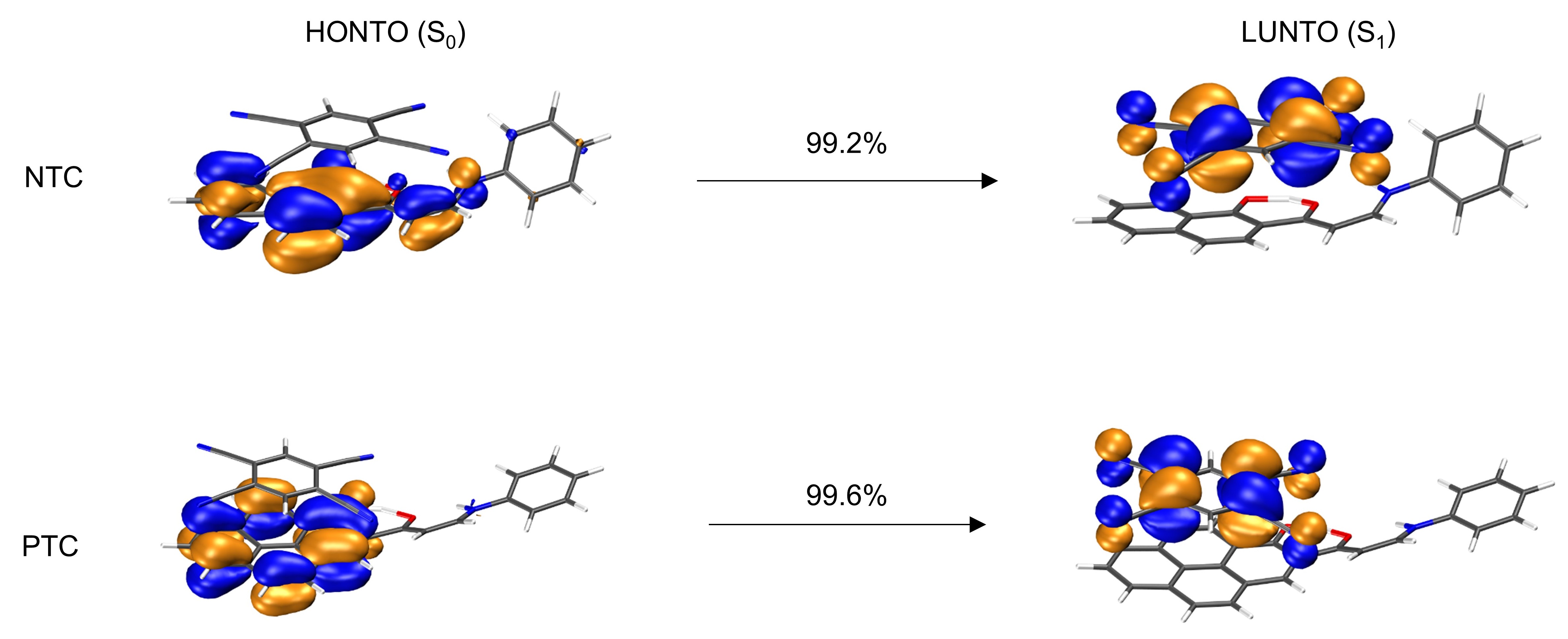


Figure S11. Natural transition orbitals (NTOs) of NTC and PTC


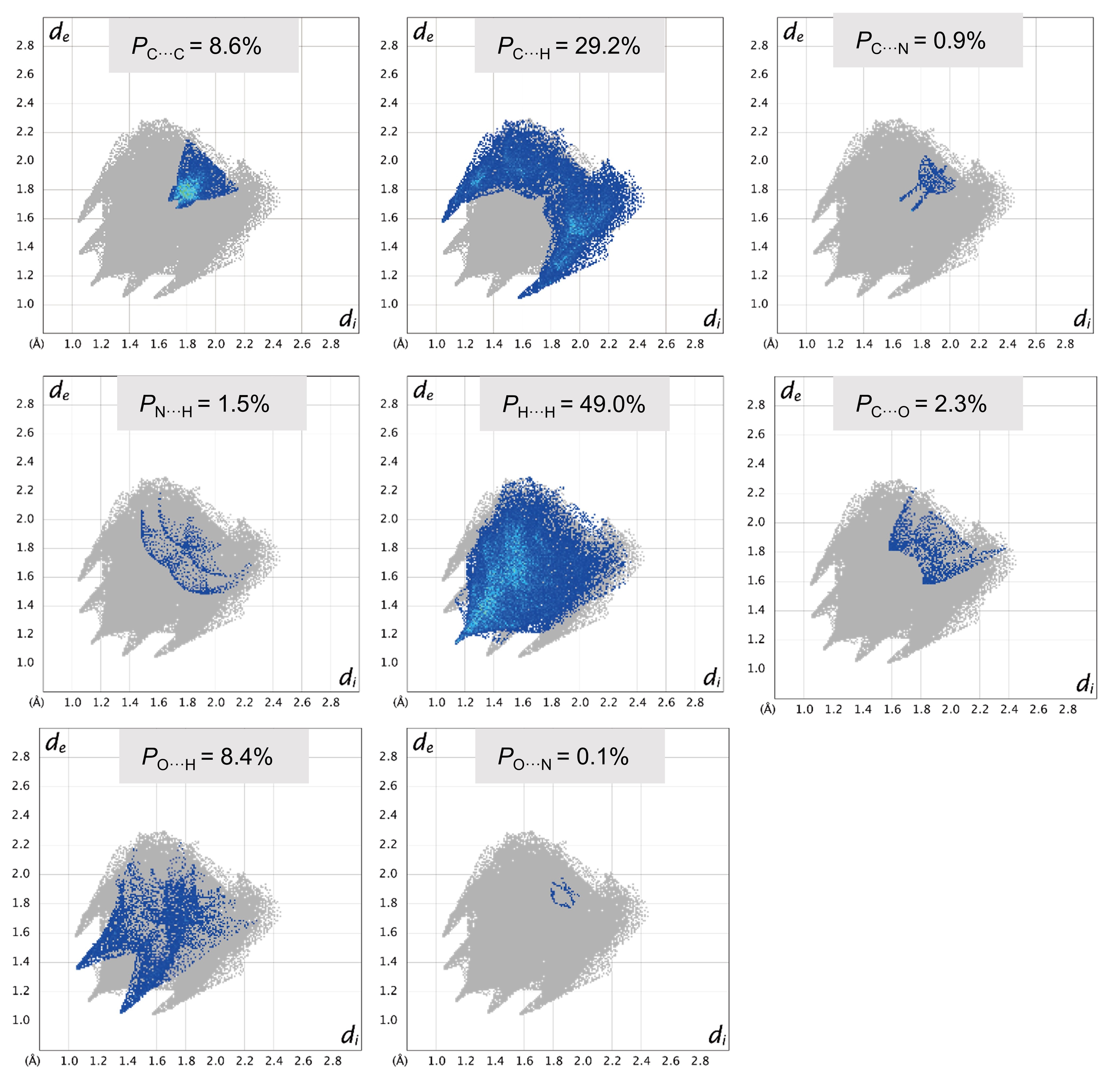


Figure S12. Hirshfeld surfaces and decomposed fingerprint plots of HNAO. Full fingerprints appeared as grey shadows underneath decomposed plots, and selected intermolecular interactions were shown as a blue shadow. The proportions (P) of different kinds of intermolecular interaction to total intermolecular interaction were also indicated.


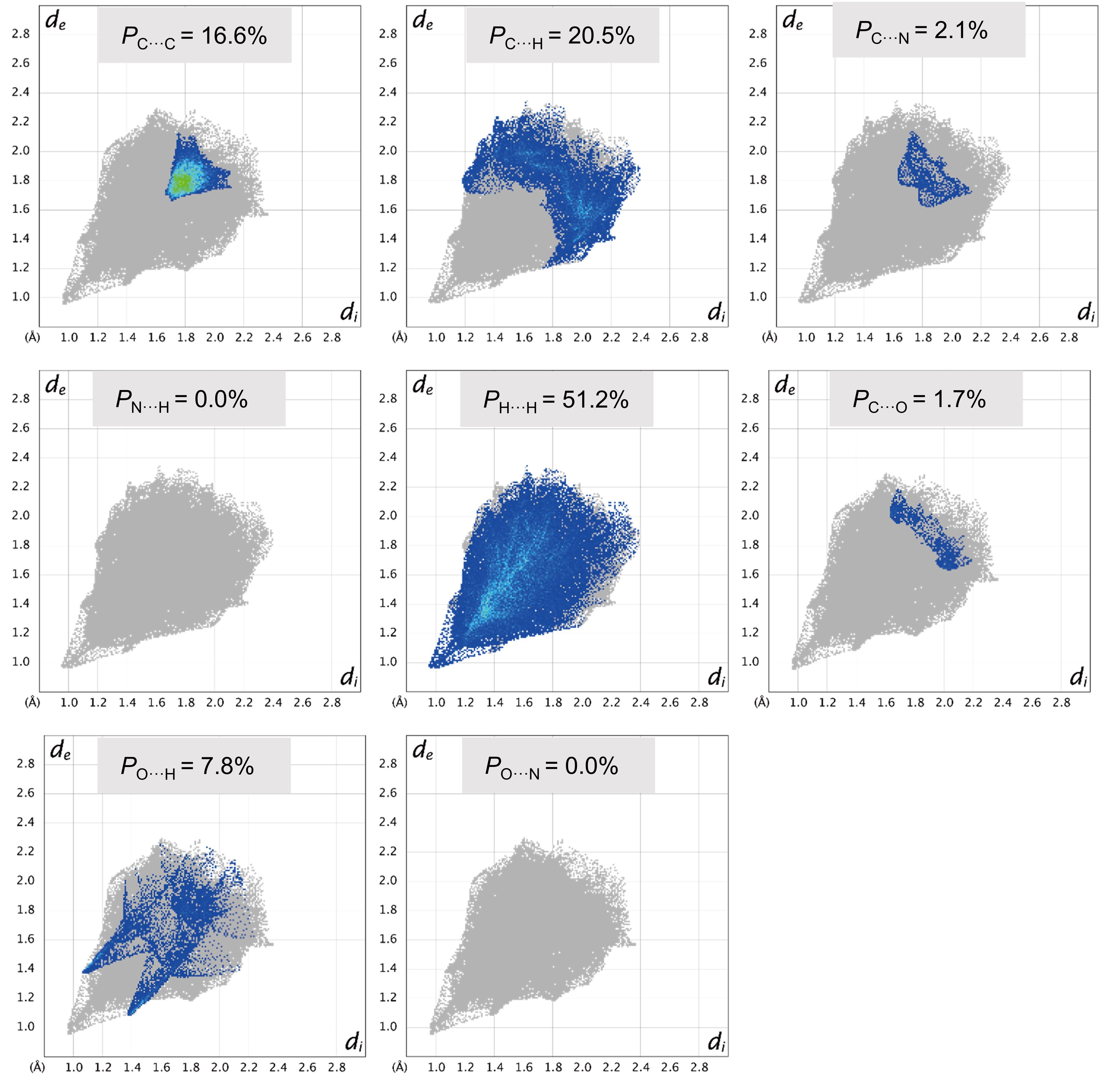


Figure S13. Hirshfeld surfaces and decomposed fingerprint plots of HPAO.


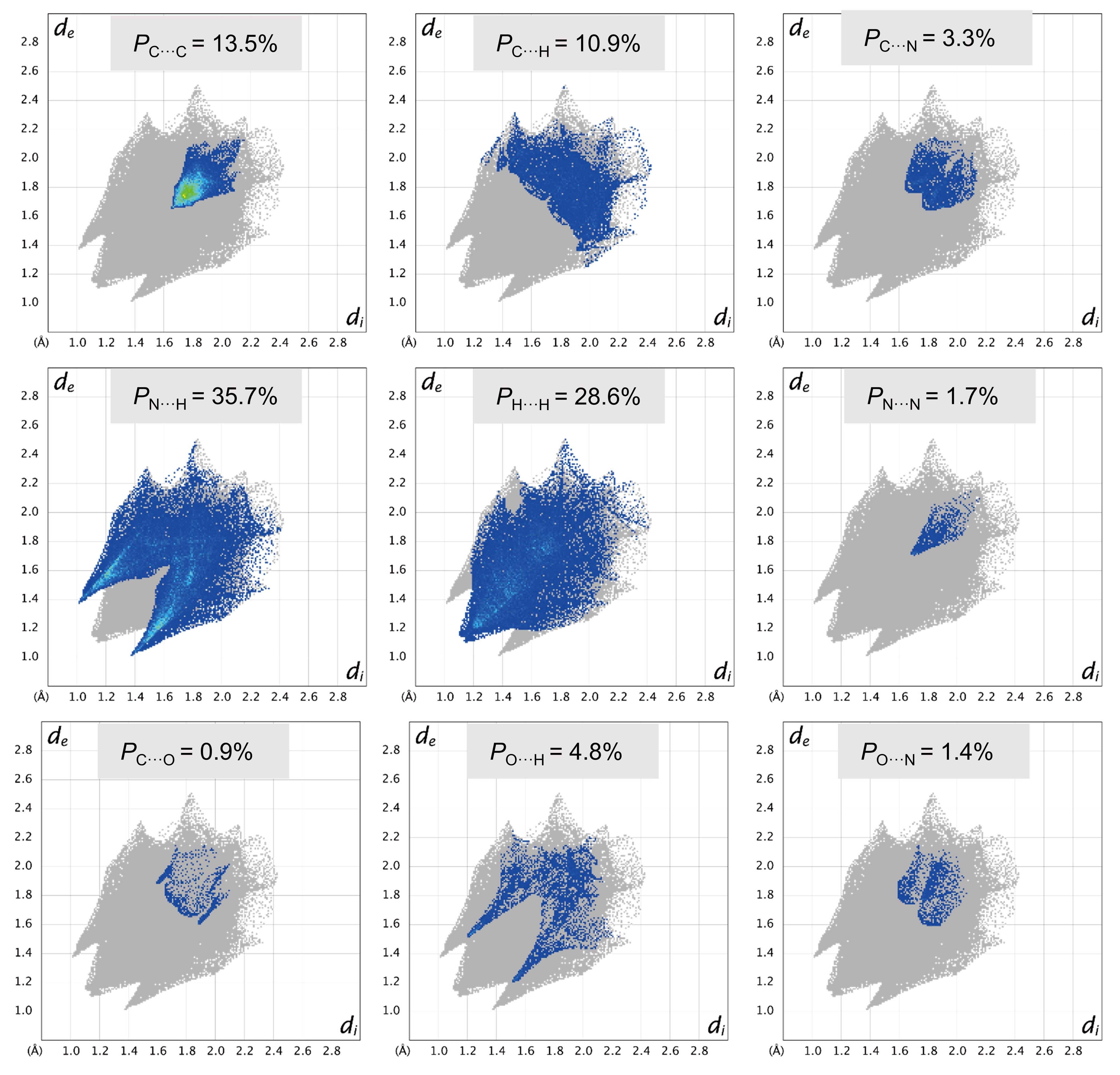


Figure S14. Hirshfeld surfaces and decomposed fingerprint plots of NTC.


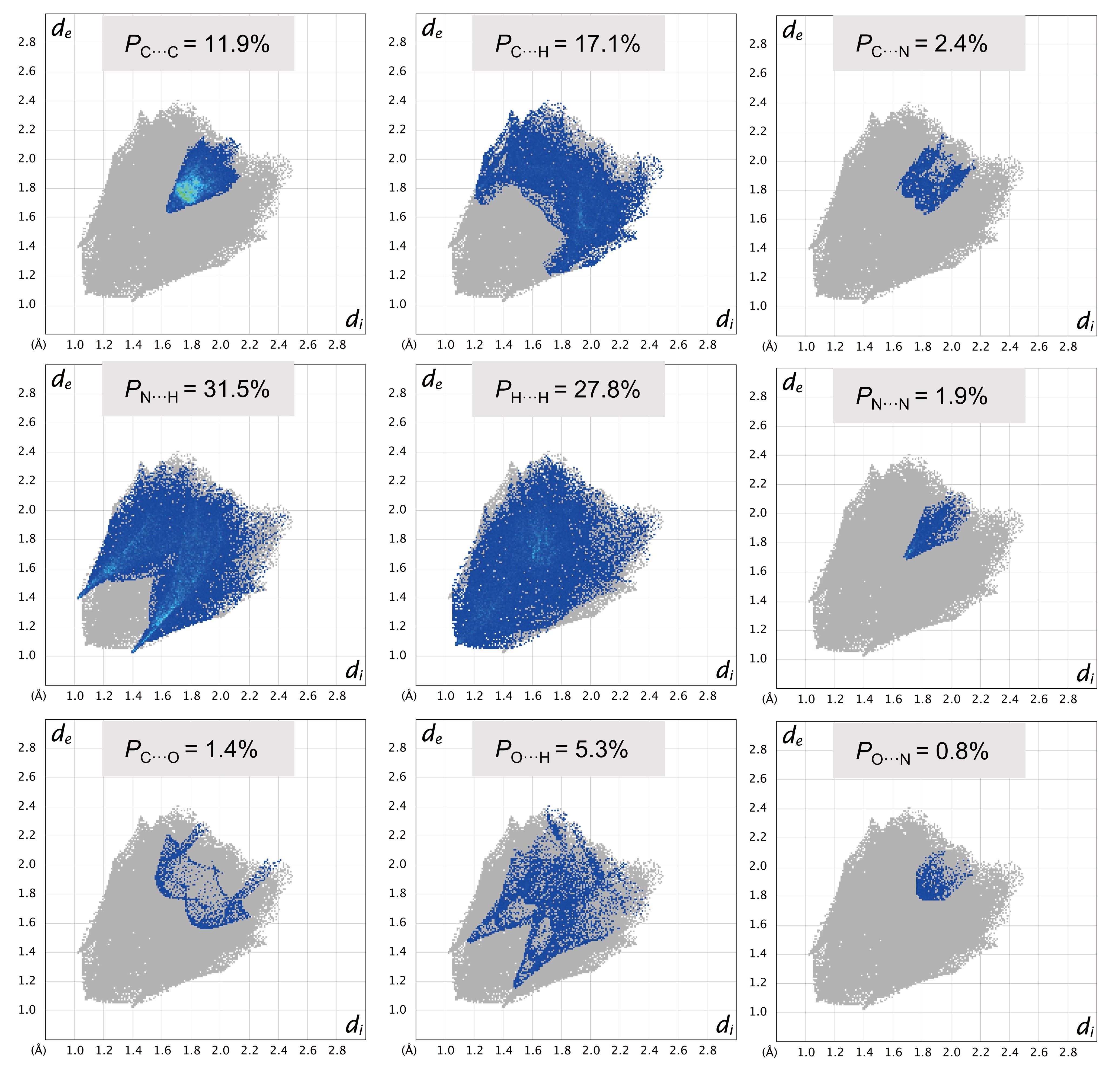


Figure S15. Hirshfeld surfaces and decomposed fingerprint plots of PTC.


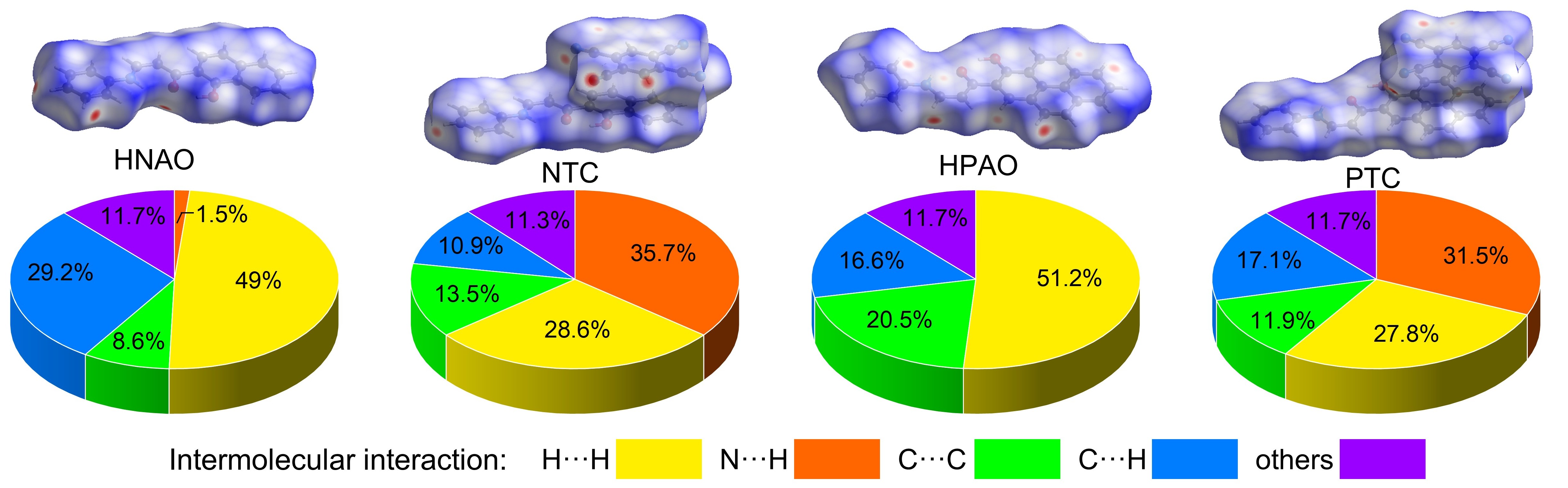


Figure S16. Hirshfeld surface analysis plots (mapped over *d*_norm_) of HNAO, NTC, HPAO and PTC (from left to right).


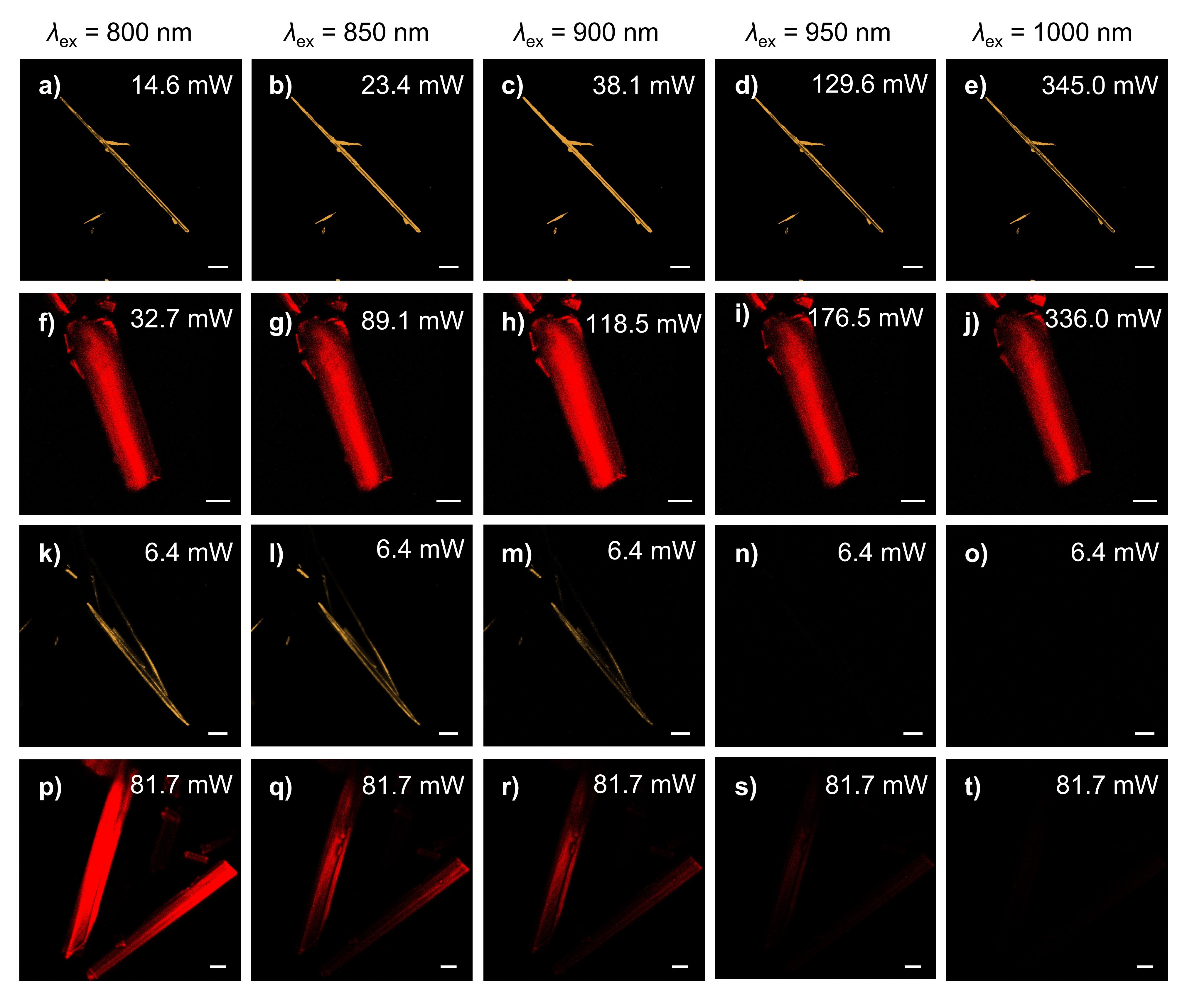


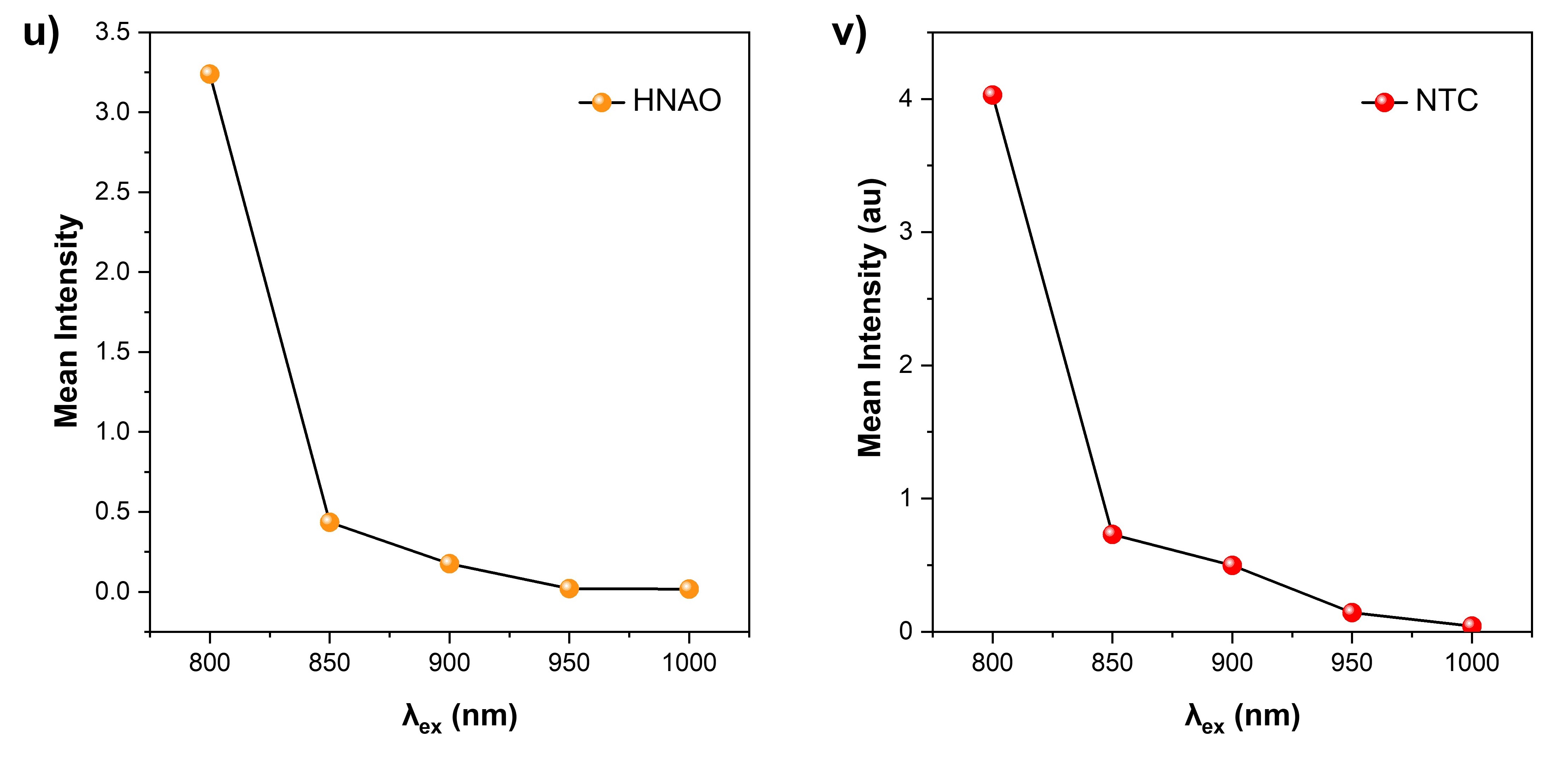


Figure S17. Two-photon excitation microscopy images of a) − e) the excitation laser powers for HNAO crystal were 14.6 mW, 23.4 mW, 38.1 mW, 129.6 mW, 345.0 mW, respectively. Scal bar: 50 μm. f) − g) the excitation laser powers for NTC were 32.7 mW, 89.1 mW, 118.5 mW, 176.5 mW, 336.0 mW, respectively. Scal bar: 10 μm. k)-o) and p)-t) the laser power to the spectra set to be 6.36 mW and 81.7 mW, respectively. Scal bar 50 μm (k − o), 5 μm (p − t). u) Excitation wavelength dependence of upconversion fluorescence intensity of u) HNAO and v) NTC.


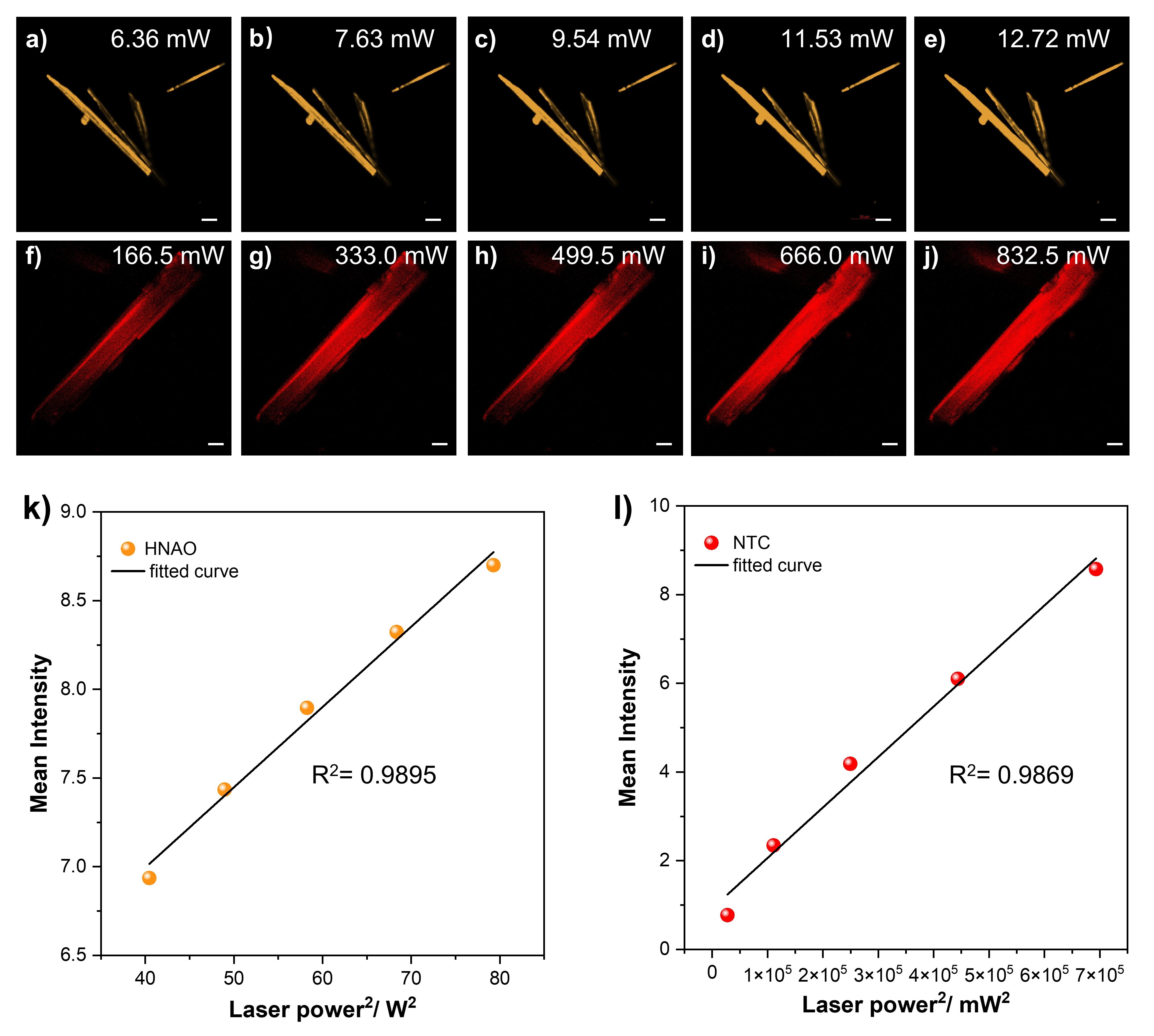


Figure S18. Two-photon excitation microscopy images at a fixed excitation wavelength of 800 nm of a) − e) the excitation laser powers for HNAO crystal were 6.36 mW, 7.63 mW, 9.54 mW, 11.53 mW, 12.72 mW, respectively. Scal bar: 50 μm. f) − g) the excitation laser powers for NTC were 166.5 mW, 333.0 mW, 499.5 mW, 666.0 mW, 832.5 mW, respectively. Scal bar: 10 μm. Excitation power dependence of upconversion fluorescence intensity of u) HNAO and v) NTC.


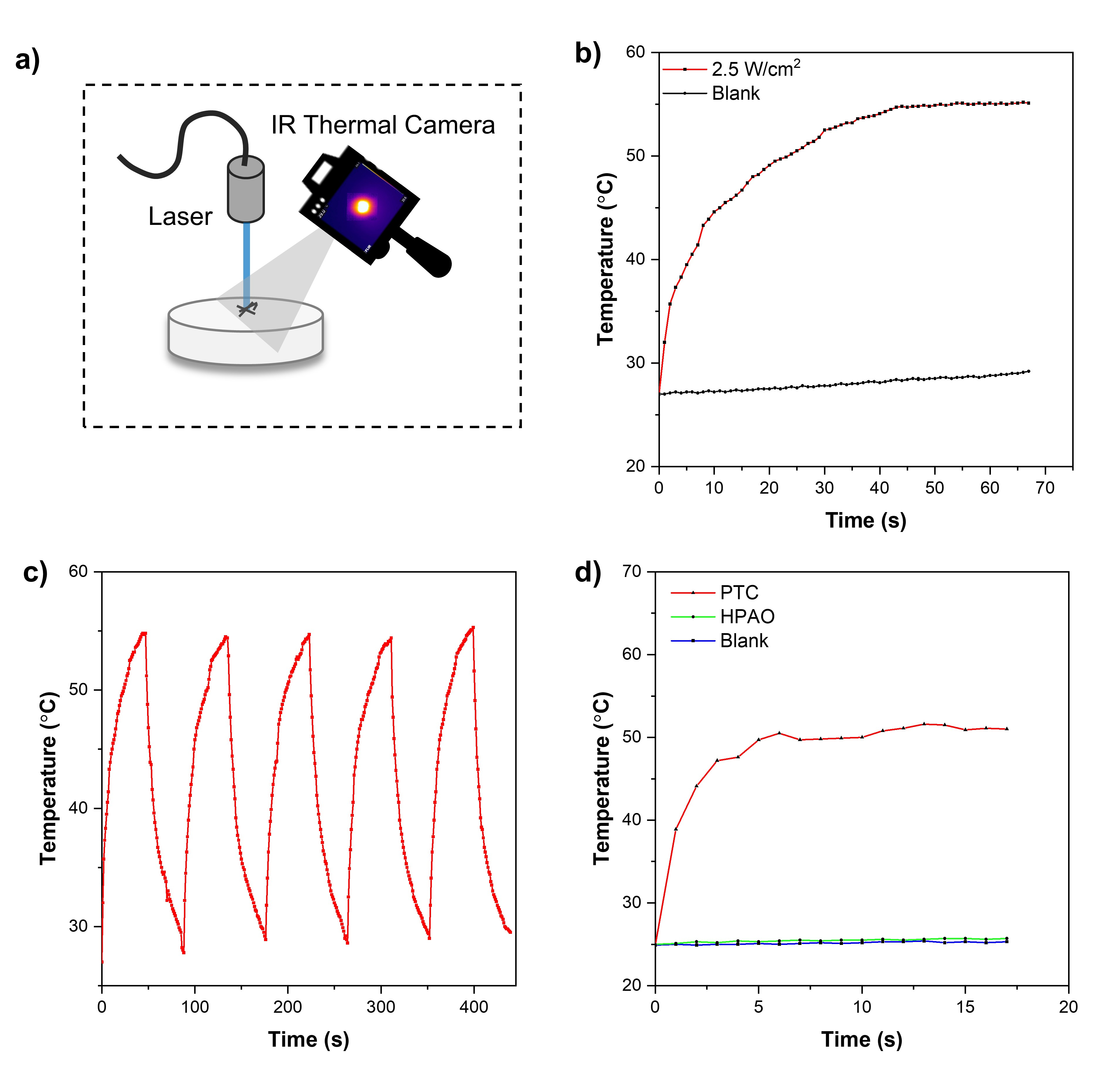


Figure S19. a) Diagram of the photothermal conversion measurement. b) Photothermal conversion curves of cocrystals under 405 nm laser irradiation with power densities of 2.5 W/cm^2^. c) temperature evolutions of HNAO before and after 5 heating/cooling cycles under 405 nm laser irradiation (2.5 W/cm^2^). d) Photothermal conversion curves of cocrystals under 660 nm laser irradiation with power densities of 0.5 W/cm^2^.


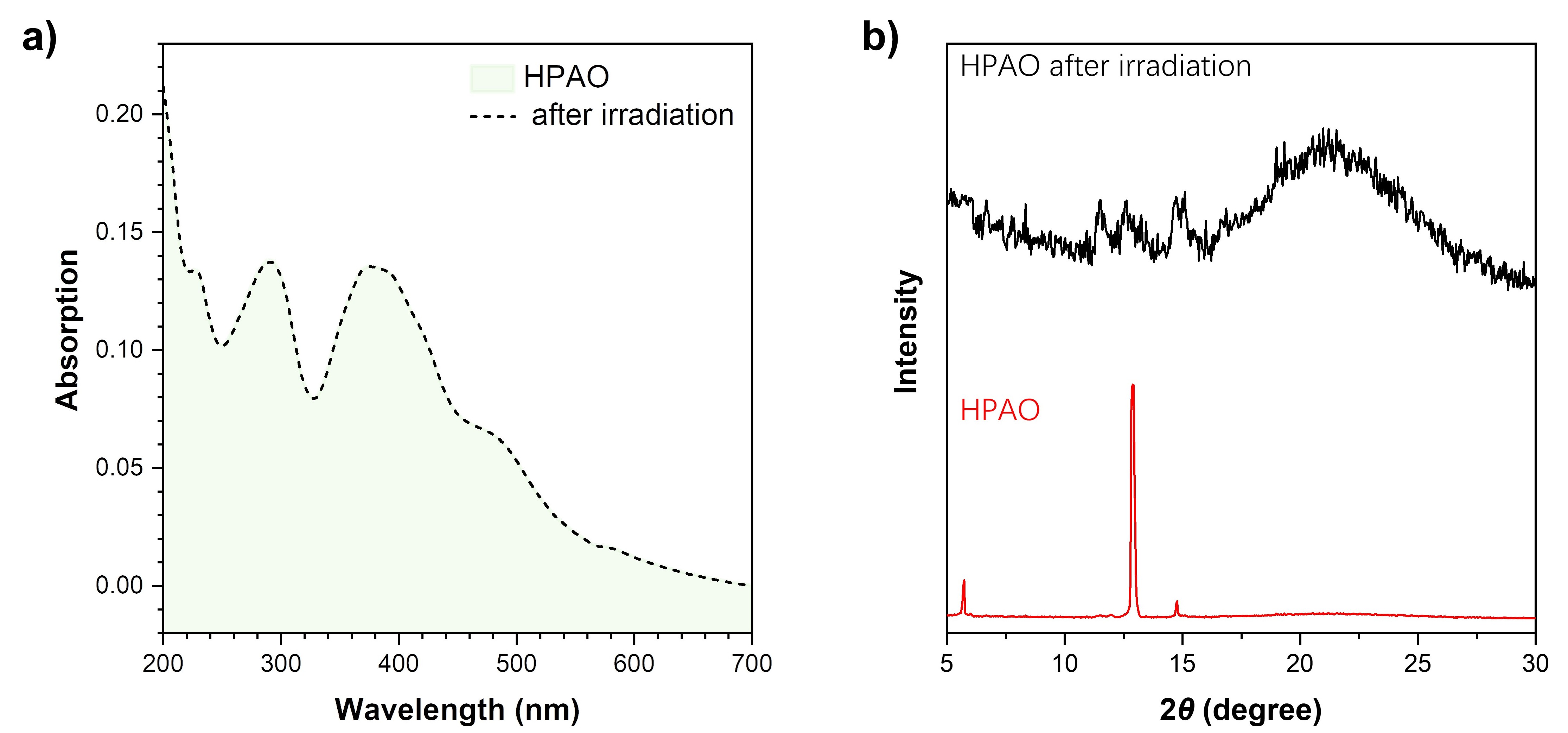


Figure S20. The PT stability of the cocrystal. a) Absorption spectra of HPAO. b) PXRD spectra of HPAO and after irradiation.


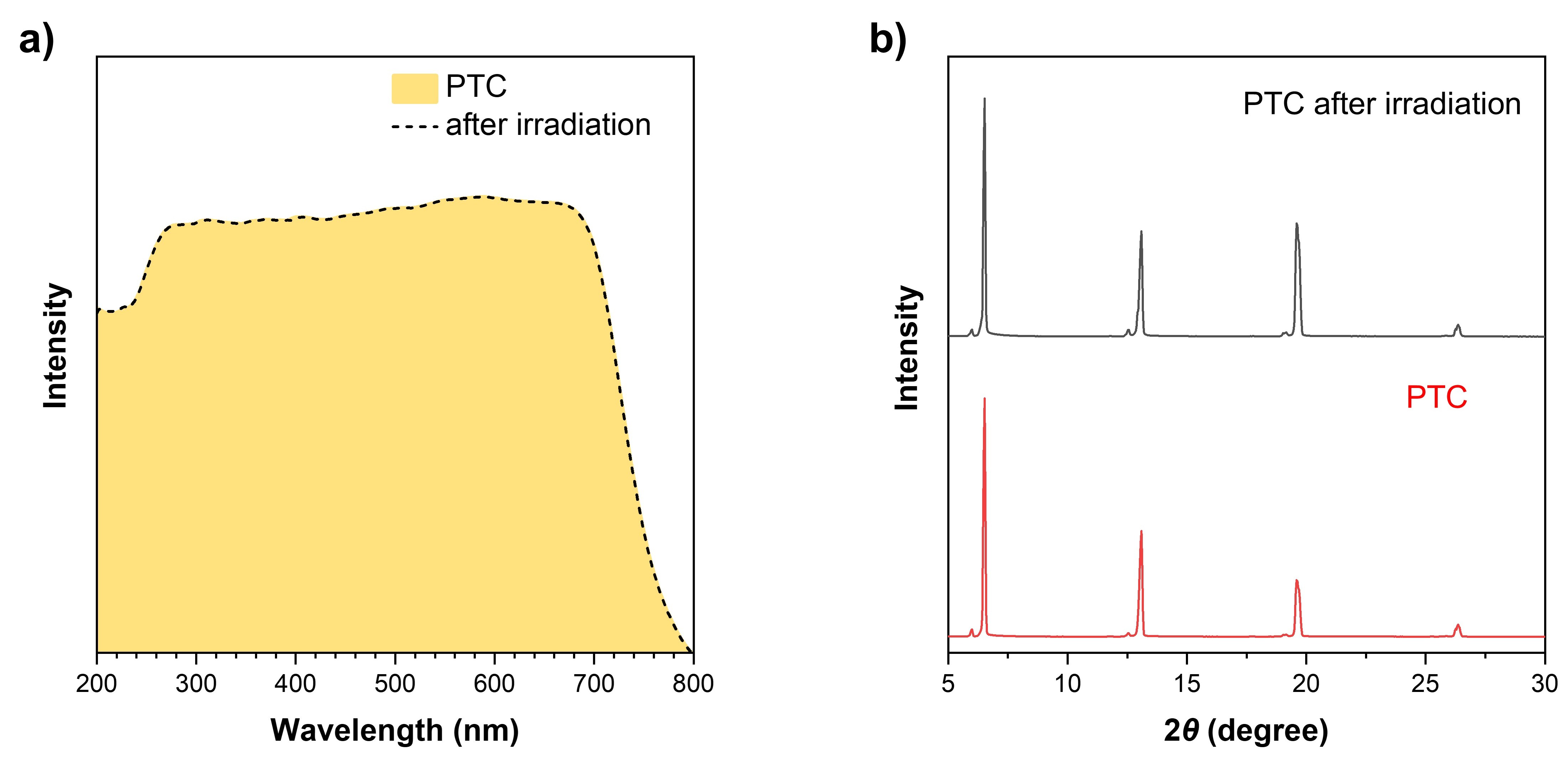


Figure S21. The PT stability of the cocrystal. a) Absorption spectra of PTC. b) PXRD spectra of PTC and after irradiation.


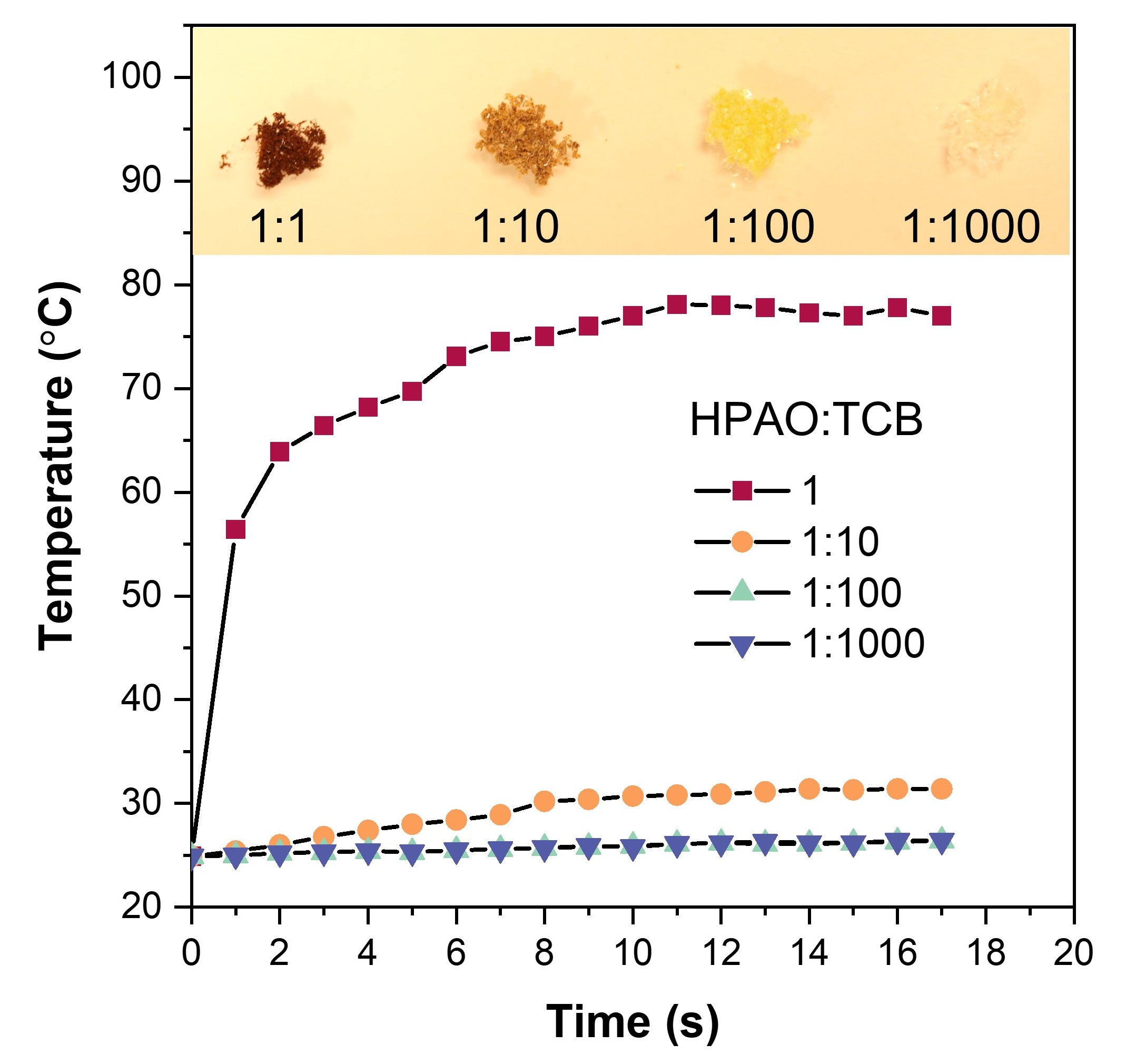


**Figure S22**. Photothermal conversion curves of different ratios of HPAO to TCB under 660 nm laser irradiation with power densities of 0.9 W/cm^2^.


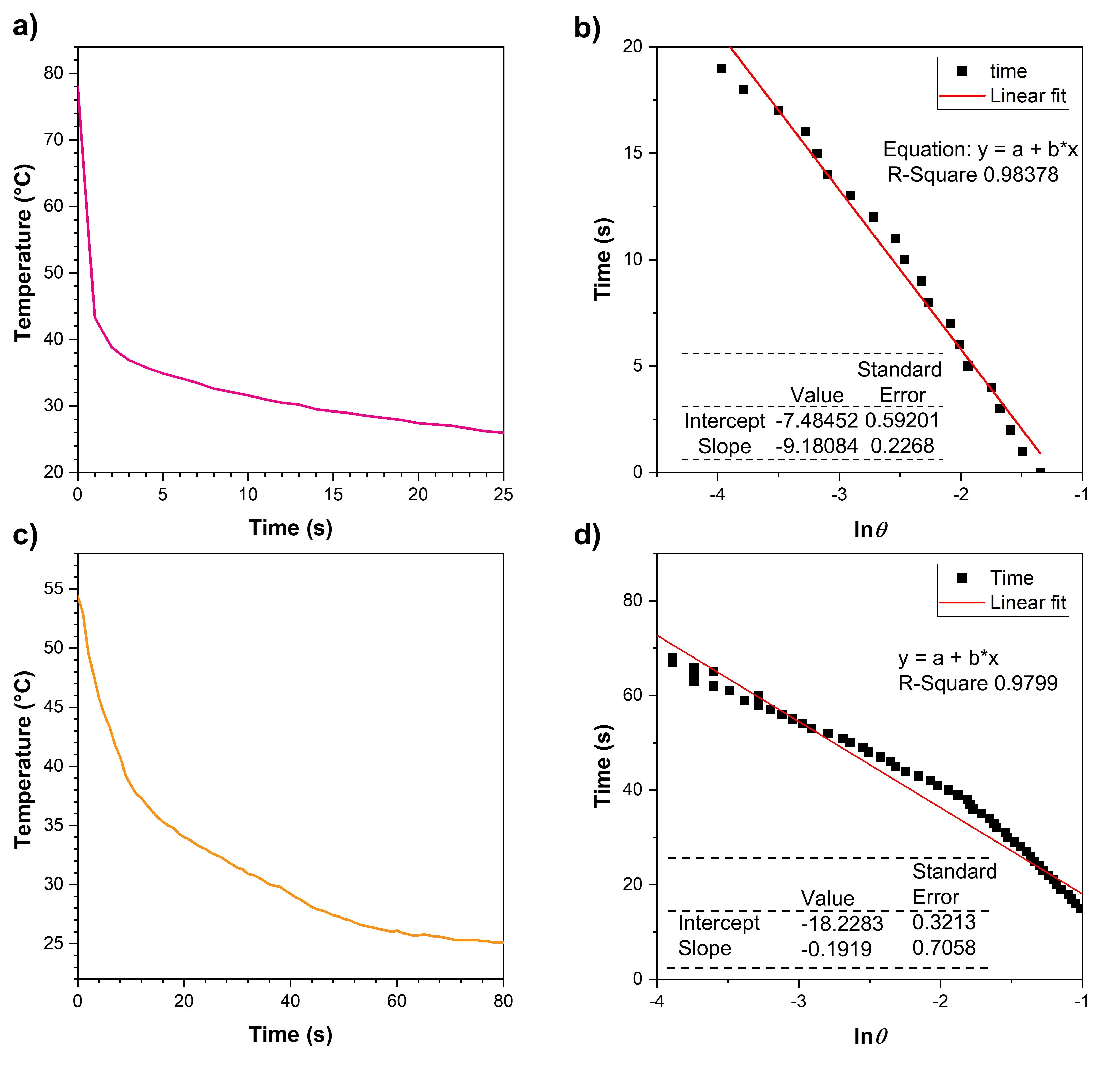


Figure S23. a) The cooling curve of PTC samples after the irradiation of 660 nm laser (0.9 W/cm^2^) and b) its corresponding time-In *θ* linear curve. c) The cooling curve of PTC samples after the irradiation of 405 nm laser (2.5 W/cm^2^) and d) its corresponding time-In *θ* linear curve.


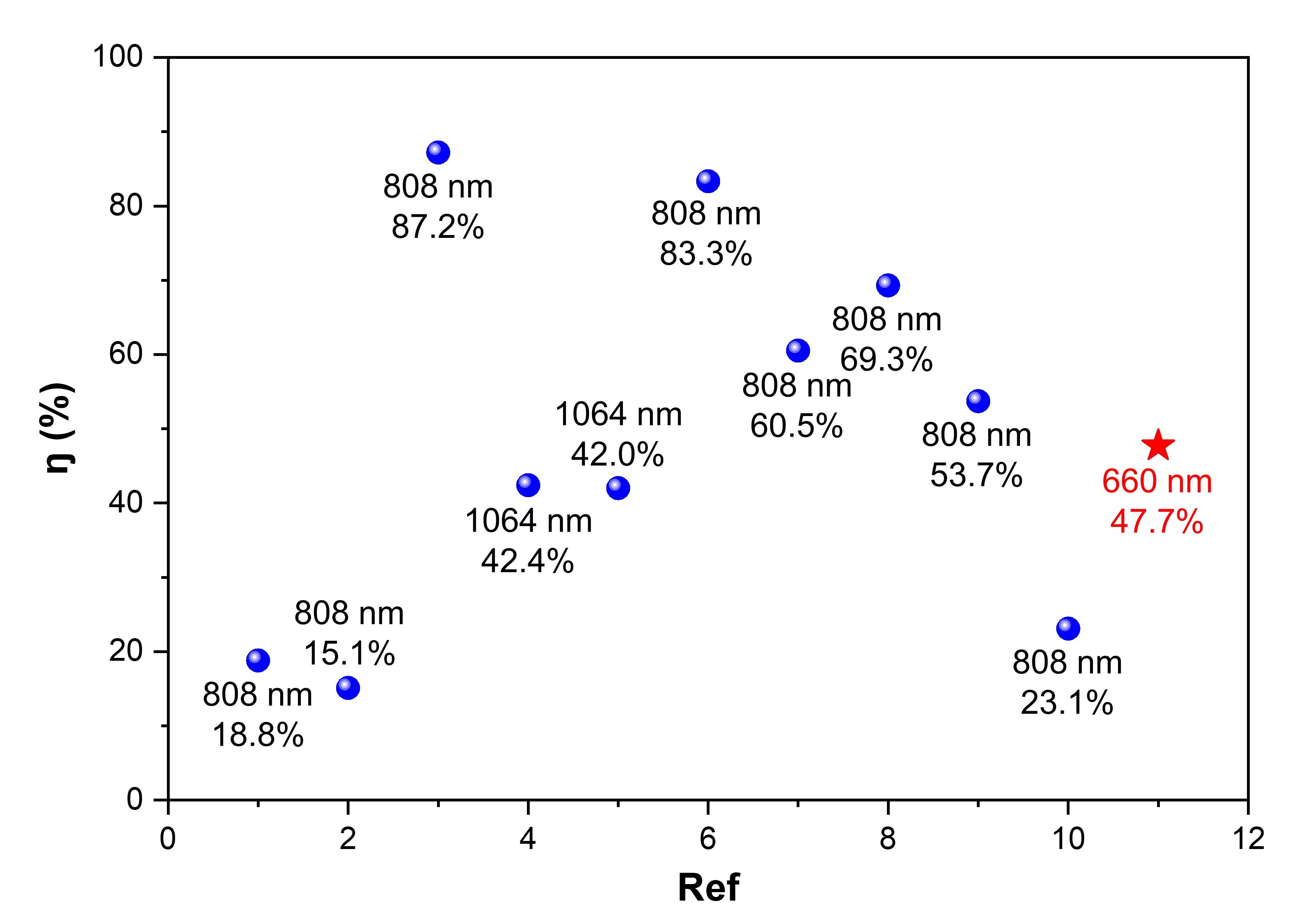


**Figure S24**. The irradiation power density and corresponding photothermal efficiency for reported organic photothermal materials^S10^.

**Table S5**. TCB bond length at 100 K^S11,S12^.

|  | α (Å) | b (Å) | c (Å) | d (Å) | e (Å) |
| --- | --- | --- | --- | --- | --- |
| TCB | 1.4009 | 1.3924 | 1.4432 | 1.1382 | 1.0214 |
| TCB^− S11^ | 1.4356 | 1.3872 | 1.4310 | 1.1521 | 0.9152 |
| TCB in NTC | 1.4043 | 1.3914 | 1.4405 | 1.1469 | 0.9501 |
| TCB in PTC | 1.4059 | 1.3963 | 1.4437 | 1.1419 | 0.9492 |


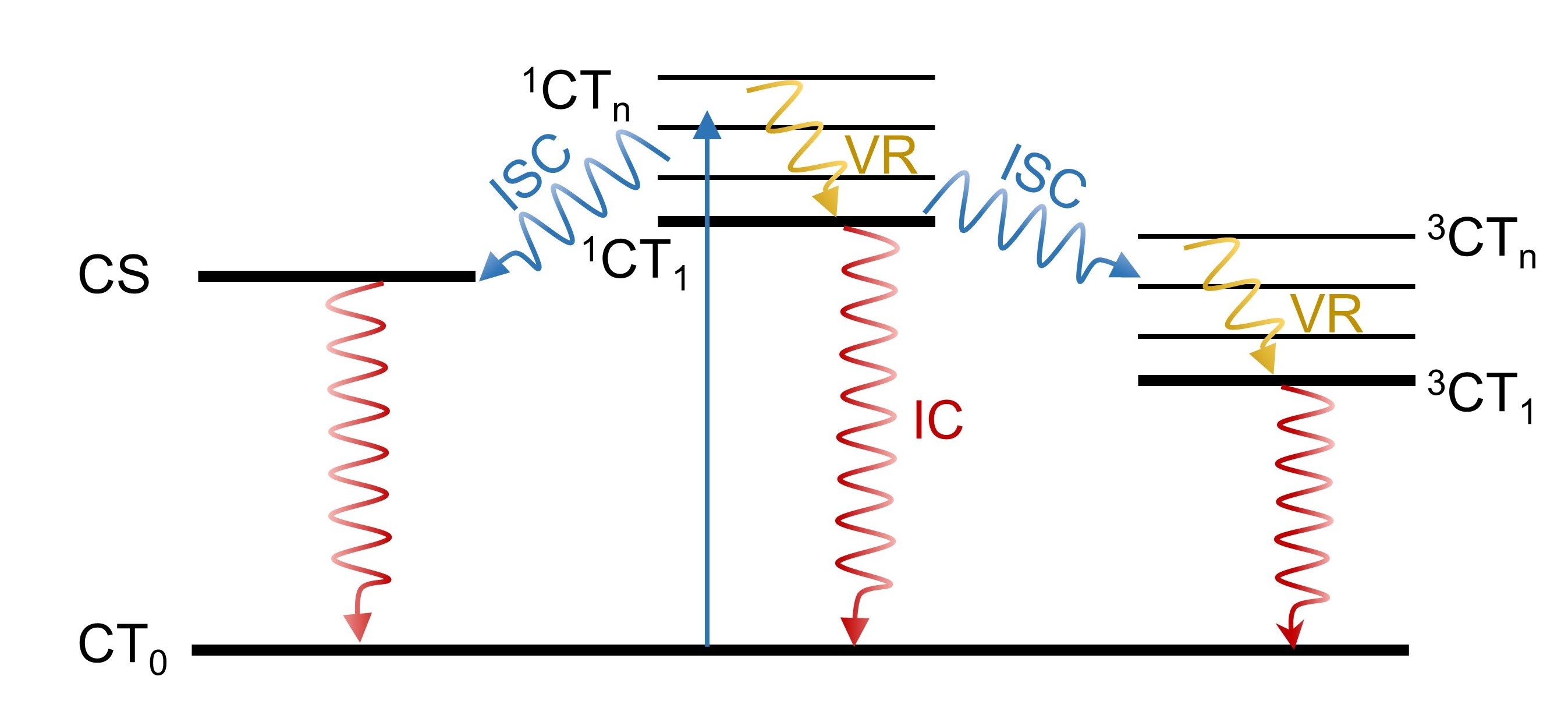


Figure S25. The TGA The Jablonski diagram of excited-state evolution for the PTC cocrystals under 660 nm pump laser. Here, IC: internal conversion; VR: vibrational relaxation; ISC: intersystem crossing.


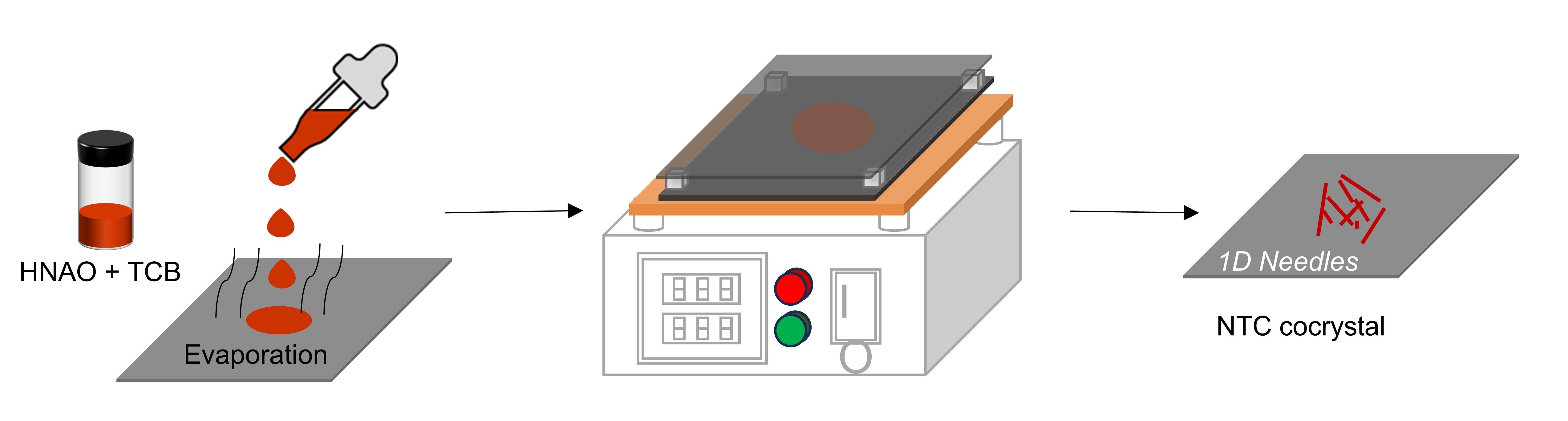


Figure S26. Diagram of microspacing in-air sublimation method growth of NTCs.


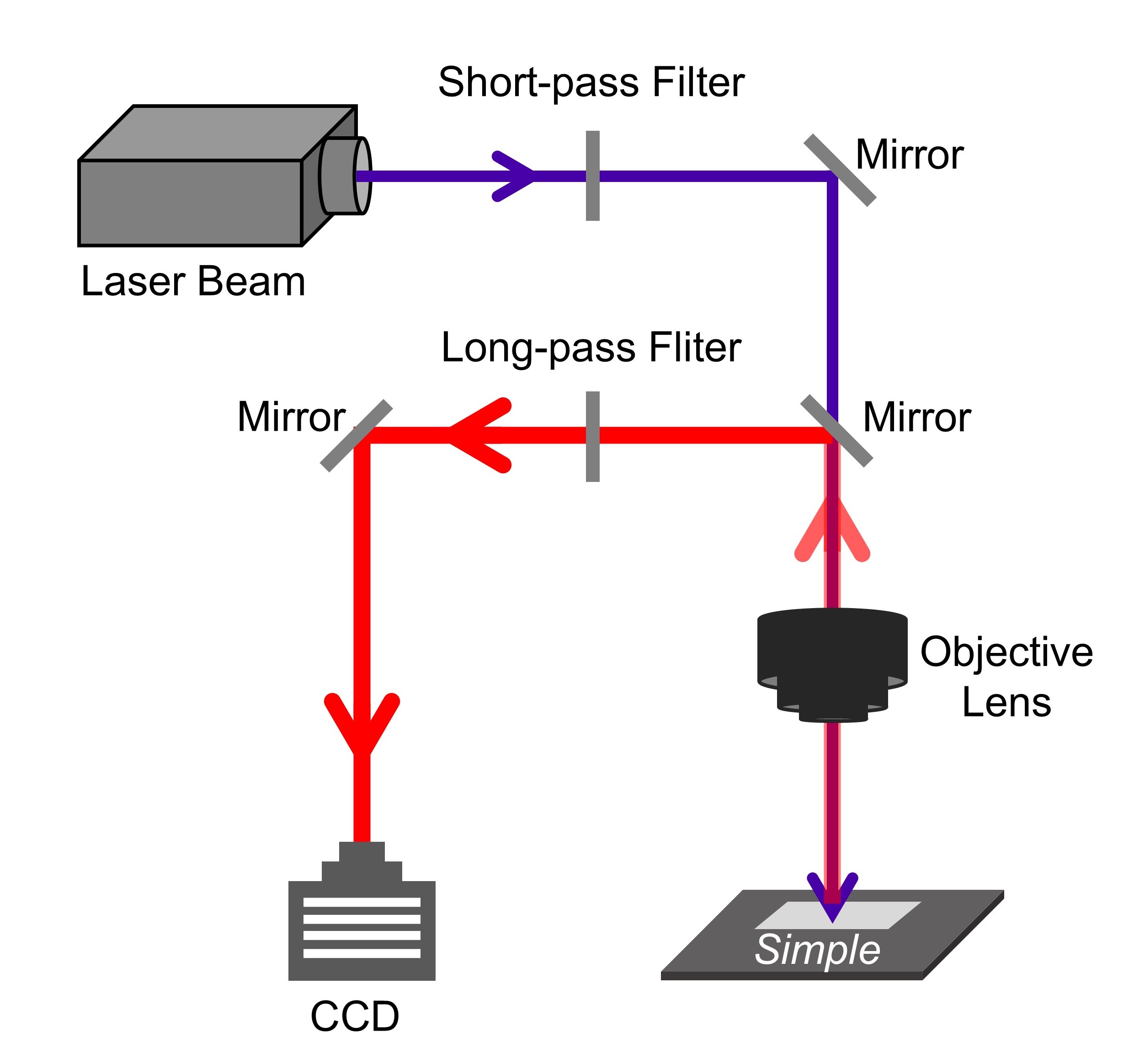


Figure S27. PL microscopy images were taken with an inverted microscope (Olympus, BX43). To measure the PL spectra of the nanowires, the samples were excited locally with a 532 nm laser with a focused beam through an objective (Nikon CFLU Plan, 50×, N.A. = 0.8). The power at the input was altered by the neutral density filters. The emission was dispersed with a grating (150 G/mm) and recorded with a thermal-electrically cooled CCD (Princeton Instruments, PIX-256E).


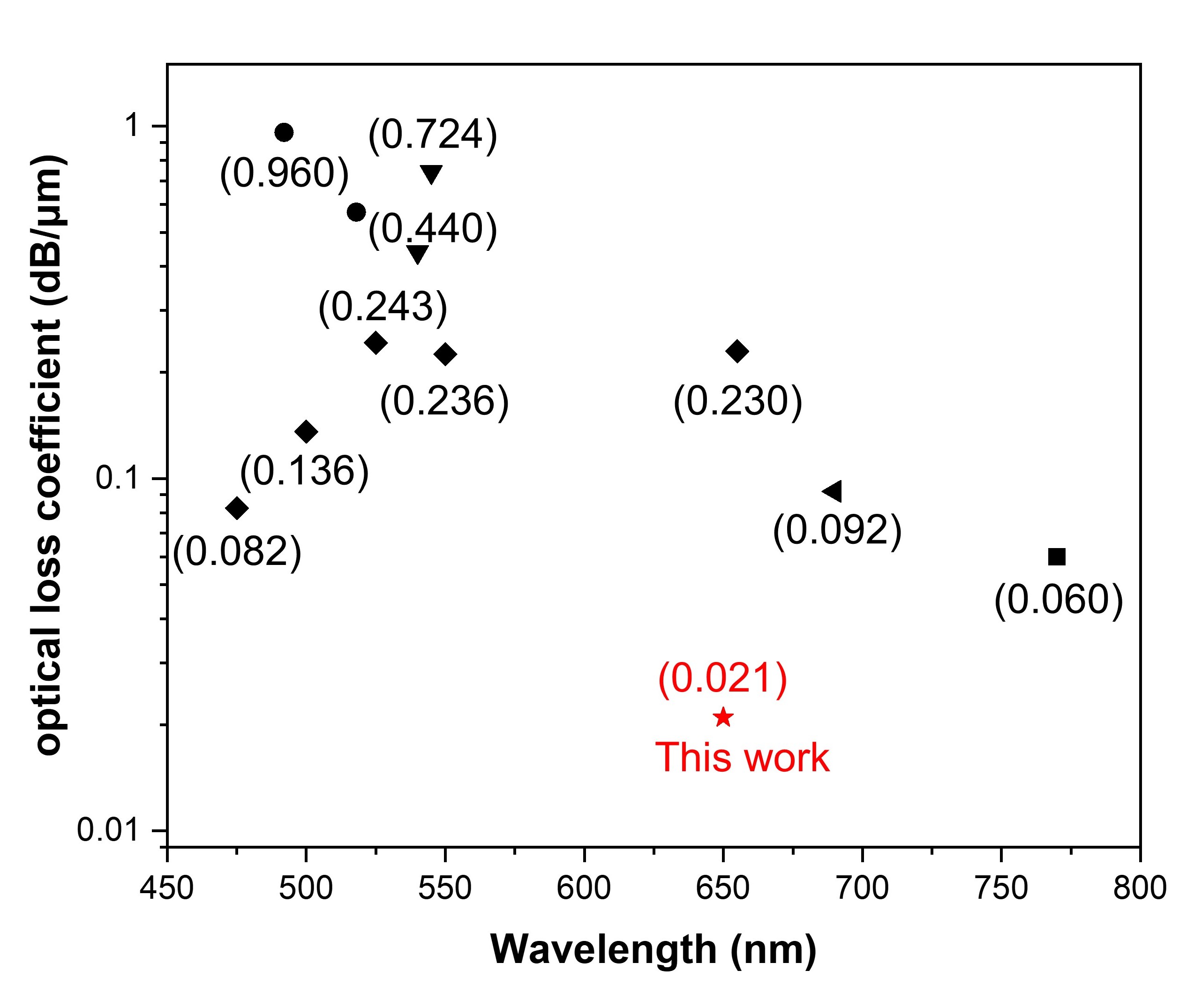


Figure S28. Reported optical waveguide properties of the organic nano-cocrystals.^[S13-S17]^


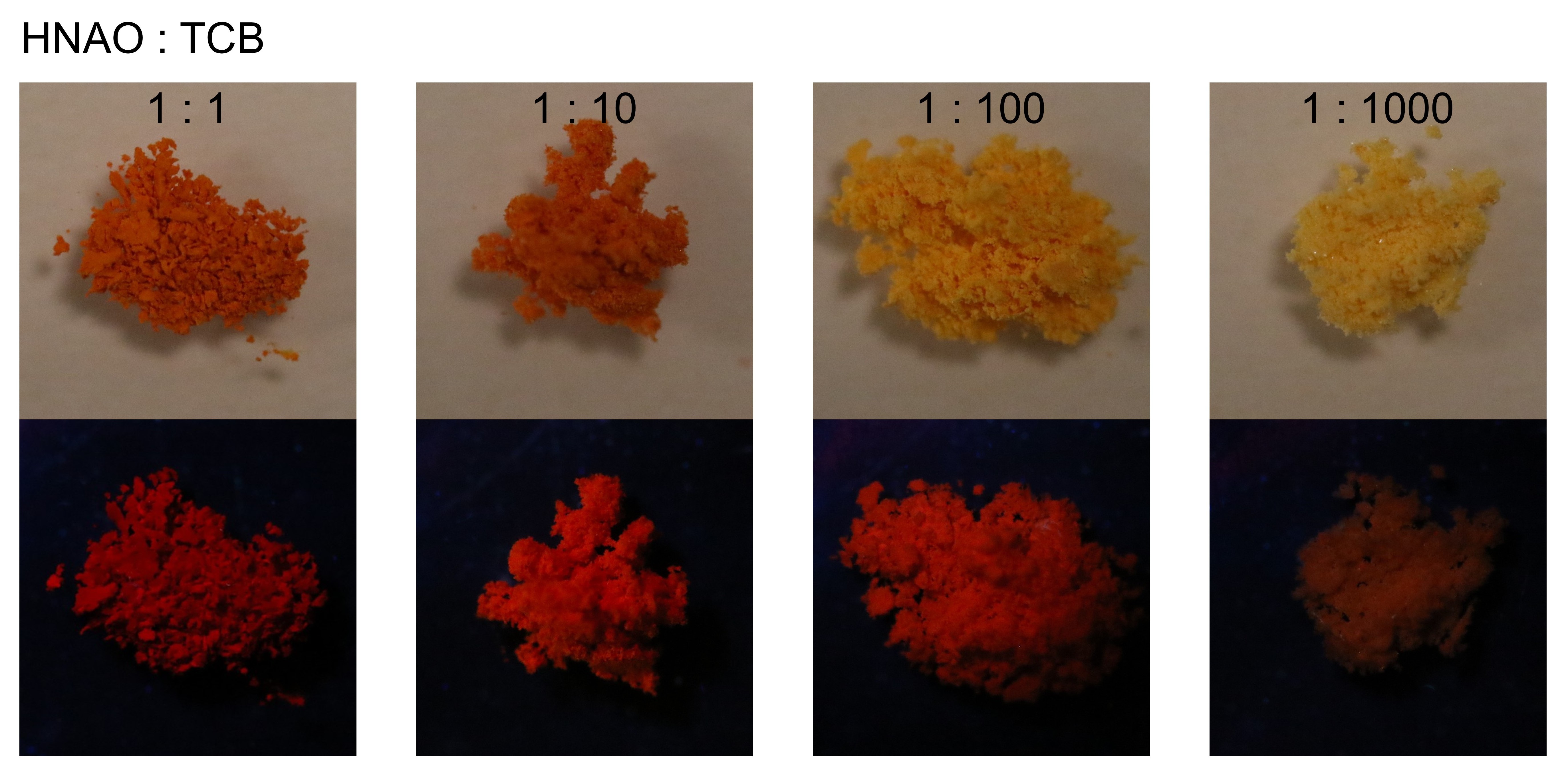


Figure S28. Photographs of HNAO and TCB mixtures at different molar ratios


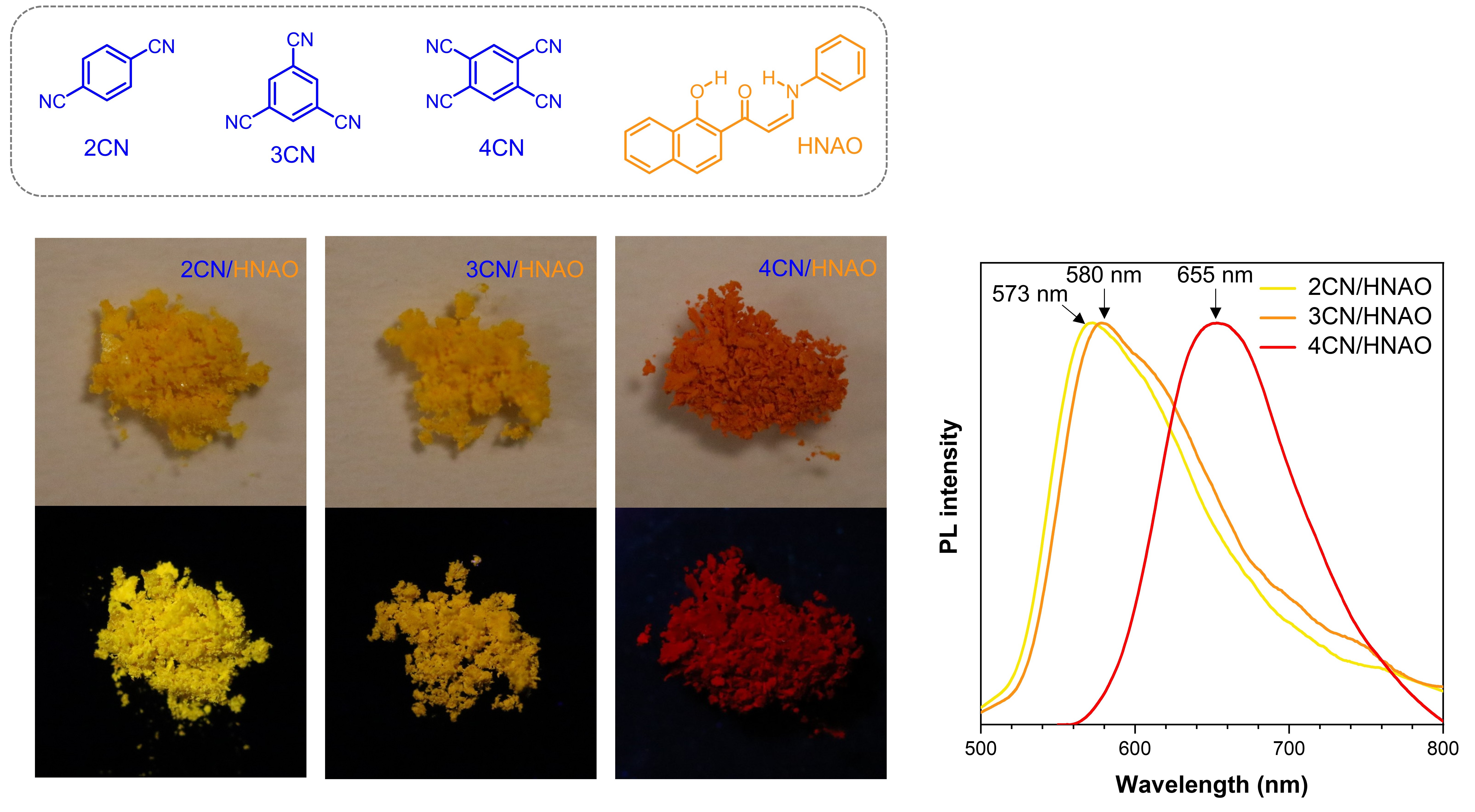


Figure S29. Photographs of 1:1 mixtures of various acceptors with the donor, along with their corresponding solid state PL spectra (λex = 405 nm).


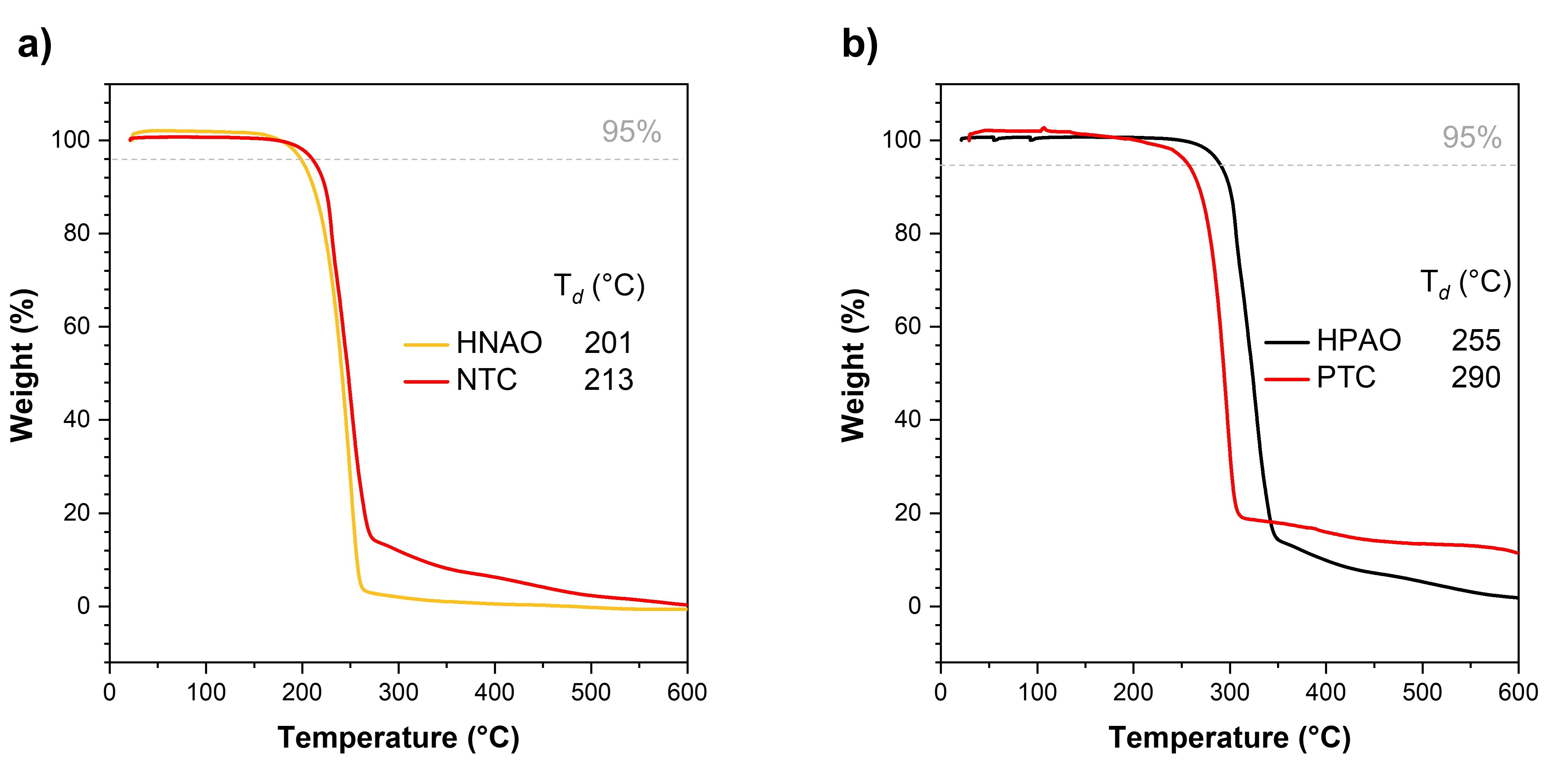


Figure S30. The TGA thermogram of a) HNAO and NTC, and b) HPAO and PTC recorded under nitrogen at a heating rate of 10 ℃/min.

# 3. Reference

[S1] Liu, Y.; Zhou, R.; Wan, J. *Synthetic Commun.* **2013**, *43*, 2475−2483,

[S2] David, A. H. G.; Casares, R. Cuerva, J. M.; Campaña, A. G.; Blanco, V. *J. Am. Chem. Soc.* **2019**, *141*, 18064−18074

[S3] Frisch, M. J.; Trucks, G. W.; Schlegel, H. B.; Scuseria, G. E.; Robb, M. A.; Cheeseman, J. R.; Scalmani, G.; Barone, V.; Petersson, G. A.; Nakatsuji, H.; Li, X.; Caricato, M.; Marenich, A. V.; Bloino, J.; Janesko, B. G.; Gomperts, R.; Mennucci, B.; Hratchian, H. P.; Ortiz, J. V.; Izmaylov, A. F.; Sonnenberg, J. L.; Williams-Young, D.; Ding, F.; Lipparini, F.; Egidi, F.; Goings, J.; Peng, B.; Petrone, A.; Henderson, T.; Ranasinghe, D.; Zakrzewski, V. G.; Gao, J.; Rega, N.; Zheng, G.; Liang, W.; Hada, M.; Ehara, M.; Toyota, K.; Fukuda, R.; Hasegawa, J.; Ishida, M.; Nakajima, T.; Honda, Y.; Kitao, O.; Nakai, H.; Vreven, T.; Throssell, K.; Montgomery, J. A., Jr.; Peralta, J. E.; Ogliaro, F.; Bearpark, M. J.; Heyd, J. J.; Brothers, E. N.; Kudin, K. N.; Staroverov, V. N.; Keith, T. A.; Kobayashi, R.; Normand, J.; Raghavachari, K.; Rendell, A. P.; Burant, J. C.; Iyengar, S. S.; Tomasi, J.; Cossi, M.; Millam, J. M.; Klene, M.; Adamo, C.; Cammi, R.; Ochterski, J. W.; Martin, R. L.; Morokuma, K.; Farkas, O.; Foresman, J. B.; Fox, D. J. Gaussian, Inc., Wallingford CT, **2016**.

[S4] Yanai, T.; Tew, D. P.; Handy, N. C. A New Hybrid Exchange–Correlation Functional Using the Coulomb-Attenuating Method (CAM-B3LYP). *Chem. Phys. Lett.* **2004**, *393*, 51−57.

[S5] Grimme, S.; Antony, J.; Ehrlich, S.; Krieg, H. A Consistent and Accurate ab Initio Parametrization of Density Functional Dispersion Correction (DFT-D) for the 94 Elements H-Pu. *J. Chem. Phys.* **2010**, *132*, 154104.

[S6] Grimme, S.; Ehrlich, S.; Goerigk, L. Effect of the Damping Function in Dispersion Corrected Density Functional Theory. *J. Compt. Chem.* **2011**, *32*, 1456–1465.

[S7] Marques, M. A. L.; Gross, E. K. U. Time-Dependent Density Functional Theory. *Annu. Rev. Phys. Chem.* **2004**, *55*, 427-455.

[S8] Lu, T.; Chen, F. Multiwfn: A Multifunctional Wavefunction Analyzer. *J. Comput. Chem.*, **2012**, *33*, 580–592.

[S9] Humphrey, W.; Dalke, A.; Schulten, K. VMD: Visual molecular dynamics. *J. Mol. Graphics*, **1996**, *14*, 33–38.

[S10] Chen, Y. T.; Zhuo, M. P.; Wen, X.; Chen, W.; Zhang, K. Q.; Li, M. D. Organic Photothermal Cocrystals: Rational Design, Controlled Synthesis, and Advanced Application. *Adv. Sci.* **2023,** *10*, e2206830

[S11] Yao, W.; Wu, P.; Xie, X.; Shen, S.; Yu, L. Synthesis and photophysical properties of charge transfer cocrystals based on TCNB and fluorene and its derivatives. [*CrystEngComm*](https://doi.org/10.1039/1466-8033/1999), **2024**, *26*, 2155−2165

[S12] Bagnato, J. D.; Shum, W. W.; Strohmeier, M.; Grant, D. M.; Arif, A. M.; Miller, J. S. The Structure of Fractionally Charged Tetracyanobenzene*^n-^* Present in [TCNB$]_{3}^{2-}$ *Angew. Chem., Int. Ed.,* **2006**, *45*, 5322−5326

[S13] Zhuo, M. P.; Yuan, Y.; Su, Y.; Chen, S.; Chen, Y. T.; Feng, Z. Q.; Qu, Y. K.; Li, M. D.; Li, Y.; Hu, B. W.; Wang, X. D.; Liao, L. S. Segregated Array Tailoring Charge-Transfer Degree of Organic Cocrystal for the Efficient Near-Infrared Emission beyond 760 nm. *Adv. Mater.* **2022,** *34*, e2107169

[S14] Barman, D.; Annadhasan, M.; Bidkar, A. P.; Rajamalli, P.; Barman, D.; Ghosh, S. S.; Chandrasekar, R.; Iyer, P. K. Highly efficient color-tunable organic co-crystals unveiling polymorphism, isomerism, delayed fluorescence for optical waveguides and cell-imaging. *Nat. Commun.* **2023**, *14*, 6648

[S15] Zhang, J.; Zhao. S.; Jiang, J.; Lv, Z.; Luo, J.; Shi, Y.; Lu, Z.; Wang, X. Organic cocrystal alloys: from three primary colors to continuously tunable emission and applications on optical waveguides and displays. *Small* **2024**, 2400313

[S16] Jiang, J.; Zhao, S.; Zhang, J.; Lv, Z.; Song, J.; Sun, Y.; Liao, L.; Wang, X. Scalable synthesis of organic core/shell architectures toward dual-wavelength optical waveguides. *Nano Lett.* **2024**, *24*, 12921−12927.

[S17] Navarro-Huerta, A.; Matsuo, T.; Mikherdov, A. S.; Blahut, J.; Bartůňkova, E.; Jiang, P.; Dračinský, M.; Teat, S.; Jin, M.; Hayashi, S.; Rodriguez-Molina, B. Optical waveguiding charge-transfer cocrystals: examining the impact of molecular rotations on their photoluminescence. *J. Am. Chem. Soc.* **2025**, *147*, 8343–8349
